# Unlocking vinpocetine’s oncostatic potential in early-stage hepatocellular carcinoma: A new approach to oncogenic modulation by a nootropic drug

**DOI:** 10.1371/journal.pone.0312572

**Published:** 2024-10-31

**Authors:** Osama A. Mohammed, Mahmoud E. Youssef, Rabab S. Hamad, Mustafa Ahmed Abdel-Reheim, Lobna A. Saleh, Mohannad Mohammad S. Alamri, Muffarah Hamid Alharthi, Jaber Alfaifi, Masoud I. E. Adam, Ali M. S. Eleragi, Ahmed Senbel, Alshaimaa A. Farrag, Assad Ali Rezigalla, Hend S. El-wakeel, Mohammed A. Attia, Hussein M. El-Husseiny, Tohada M. AL-Noshokaty, Ahmed S. Doghish, Ahmed Gaafar Ahmed Gaafar, Sameh Saber

**Affiliations:** 1 Department of Pharmacology, College of Medicine, University of Bisha, Bisha, Saudi Arabia; 2 Department of Pharmacology, Faculty of Pharmacy, Delta University for Science and Technology, Gamasa, Egypt; 3 Biological Sciences Department, College of Science, King Faisal University, Al Ahsa, Saudi Arabia; 4 Central Laboratory, Theodor Bilharz Research Institute, Giza, Egypt; 5 Department of Pharmaceutical Sciences, College of Pharmacy, Shaqra University, Shaqra, Saudi Arabia; 6 Department of Pharmacology and Toxicology, Faculty of Pharmacy, Beni-Suef University, Beni Suef, Egypt; 7 Department of Clinical Pharmacology, Faculty of Medicine, Ain Shams University, Cairo, Egypt; 8 Department of Pharmacology and Toxicology, College of Pharmacy, Taif University, Taif, Saudi Arabia; 9 Department of Family and Community Medicine, College of Medicine, University of Bisha, Bisha, Saudi Arabia; 10 Department of Child Health, College of Medicine, University of Bisha, Bisha, Saudi Arabia; 11 Department of Medical Education and Internal Medicine, College of Medicine, University of Bisha, Bisha, Saudi Arabia; 12 Department of Microorganisms and Clinical Parasitology, College of Medicine, University of Bisha, Bisha, Saudi Arabia; 13 Department of Surgical Oncology, Oncology Center, Faculty of Medicine, Mansoura University, Mansoura, Egypt; 14 Department of Histology and Cell Biology, Faculty of Medicine, Assiut University, Assiut, Egypt; 15 Department of Anatomy, College of Medicine, University of Bisha, Bisha, Saudi Arabia; 16 Physiology Department, Benha Faculty of Medicine, Benha University, Qalubyia, Egypt; 17 Physiology Department, Al-Baha Faculty of Medicine, Al-Baha University, Al-Baha, Saudi Arabia; 18 Department of Clinical Pharmacology, Faculty of Medicine, Mansoura University, Mansoura, Egypt; 19 Department of Basic Medical Sciences, College of Medicine, AlMaarefa University, Riyadh, Saudi Arabia; 20 Cooperative Department of Veterinary Medicine, Faculty of Agriculture, Tokyo University of Agriculture and Technology, Tokyo, Japan; 21 Department of Surgery, Anesthesiology, and Radiology, Faculty of Veterinary Medicine, Benha University, Benha, Egypt; 22 Biochemistry Department, Faculty of Pharmacy, Heliopolis University, Cairo, Egypt; 23 Department of Biochemistry, Faculty of Pharmacy, Badr University in Cairo, Cairo, Egypt; 24 Department of Biochemistry and Molecular Biology, Faculty of Pharmacy (Boys), Al-Azhar University, Cairo, Egypt; 25 Department of Pharmacology and Toxicology, Faculty of Pharmacy, Port Said University, Port Said, Egypt; Kwame Nkrumah University of Science and Technology, GHANA

## Abstract

The development of new drugs for the inhibition of hepatocellular carcinoma (HCC) development and progression is a critical and urgent need. The median survival rate for HCC patients remains disappointingly low. Vinpocetine is a safe nootropic agent that is often used to enhance cognitive function. The impact of vinpocetine on HCC development and progression has not been fully explored. Our main objective was to investigate the possible inhibitory role of vinpocetine in rats exposed to diethylnitrosamine. We observed that vinpocetine increased the survival rate of these rats and improved the ultrastructure of their livers. Additionally, vinpocetine reduced the liver weight index, mitigated liver oxidative stress, and improved liver function. In both in vitro and in vivo settings, vinpocetine demonstrated antiproliferative and apoptotic properties. It downregulated the expression of CCND1 and Ki-67 while exhibiting anti-BCL-2 effects and enhancing the levels of Bax and cleaved caspase-3. Vinpocetine also successfully deactivated NF-κB, STAT3, and HIF-1α, along with their associated transcription proteins, thereby exerting anti-inflammatory and anti-angiogenic role. Furthermore, vinpocetine showed promise in reducing the levels of ICAM-1 and TGF-β1 indicating its potential role in tissue remodeling. These findings strongly suggest that vinpocetine holds promise as a hepatoprotective agent by targeting a range of oncogenic proteins simultaneously. However, further approaches are needed to validate and establish causal links between our observed effects allowing for a more in-depth exploration of the mechanisms underlying vinpocetine’s effects and identifying pivotal determinants of outcomes.

## Introduction

Hepatocellular carcinoma (HCC) is frequently observed in individuals who have chronic liver conditions such as cirrhosis, and it stands as the prevailing type of liver cancer [1]. The early stages of the disease do not present any symptoms, but the later stages may lead to liver failure [2, 3]. The exact cause of this condition is not clear [4] but proposed causes include chronic infection with certain hepatitis viruses and DNA mutation of the liver cells [5, 6]. Treatment choices for advanced HCC are constrained particularly conventional cytotoxic medications, often proving ineffective [7–9]. Sorafenib has stood as the solitary systemic drug demonstrating clinical efficacy for advanced HCC for over a decade [10]. However, a limited increase in patient survival rate has been associated with its use [11, 12].

Vinpocetine is a synthetic chemical substance derived from vincamine, a substance found in the periwinkle plant (Vinca minor) [13]. Vinpocetine might increase blood flow to the brain and protect brain neurons against injury [14]. Additionally, it is used to enhance memory and increase brain metabolism [15]. The proposed mechanism of action for vinpocetine encompasses various potential effects, including anti-inflammatory and antioxidant activity [16]. Studies have suggested that vinpocetine may inhibit IκB kinase β (IKKβ) [17], thereby preventing the degradation of the inhibitor of nuclear factor kappa B (IκB) and subsequent translocation of nuclear transcription factor kappa B (NF-κB) to the cell nucleus [18].

NF-κB inhibition emerges as a promising avenue in the quest for antitumor therapies. This inhibition holds the potential to exert formidable antitumor effects by preventing the expression of genes that underpin tumor survival, proliferation, and their notorious resistance to therapeutic drugs [19, 20]. The multifaceted ways in which NF-κB inhibition can bolster the antitumor response are noteworthy. *NF-κB* inhibition can downregulate B-cell lymphoma 2 (*BCL-2*), thus enhancing the susceptibility of tumor cells to programmed cell death and rendering them more receptive to the potent effects of chemotherapy drugs [21].

Laboratory experiments conducted both in vitro and in vivo have revealed that vinpocetine exhibits antitumor activity against human breast cancer cells by inducing cell cycle arrest in the G0/G1 phase and by influencing mitochondrial pathways involved in apoptosis [22]. Moreover, it showed an enhanced anticancer activity when combined with sorafenib via modulation of phosphoinositide 3-kinase (*PI3K*)/protein kinase B (*AKT*)/glycogen synthase kinase 3 beta (*GSK-3β*) signaling axis [23]. Vinpocetine may also increase the effects of radiation by increasing tumor oxygenation [24].

It is proposed that vinpocetine has been observed to directly inhibit the activity of IKK, exerting control over *NF-κB* signaling, thereby exerting anti-inflammatory effects and potentially influencing cell fate [17]. Additionally, vinpocetine stimulates the mitochondrial pathway of apoptosis, wherein cytochrome c is released from the mitochondria into the cytosol, thereby instigating the activation of caspases, the enzymes responsible for orchestrating apoptosis [22]. Furthermore, vinpocetine regulates the expression of genes associated with apoptosis, favoring the augmentation of pro-apoptotic genes such as BCL-2-associated X protein (*Bax*) and *BCL-2* homologous antagonist/killer (Bak) while simultaneously diminishing the presence of anti-apoptotic genes like *BCL-2* and B-cell lymphoma-extra-large (*Bcl-xL*) [22].

A previous study has indicated the potential anti-HCC effects of vinpocetine in HCC cells [23]. One of the key limitations of this study lies in its focus on a narrow aspect of molecular pathways, such as the modulation of the *PI3K/AKT/GSK-3β* axis. Additionally, the authors did not explore other critical molecular pathways involved in HCC progression. Furthermore, the study was limited to in vitro experiments, and the in vivo relevance of the findings remains uncertain. The potential impact of vinpocetine on the tumor microenvironment, immune response, and angiogenesis, all of which are pivotal in HCC development, were also not addressed.

The impact of vinpocetine on HCC development and progression in a diethylnitrosamine (DENA)-induced HCC rat model has not been fully explored. Consequently, our objective was to investigate the potential oncostatic properties of vinpocetine using both in vitro and in vivo models, specifically focusing on rat livers intoxicated with DENA. We employed methodologies including animal modeling, histopathological examination, and molecular analysis of key signaling pathways to evaluate vinpocetine’s anti-inflammatory, antiangiogenic, antiproliferative, and apoptotic effects to investigate the oncostatic effects of vinpocetine on the early stages of HCC. Our results demonstrated that vinpocetine significantly increased survival rates, reduced liver weight index, mitigated liver oxidative stress, and improved liver function in DENA-intoxicated rats. Specifically, vinpocetine significantly decreased the levels of inflammatory markers such as NF-κB p65, IL-6, MCP-1, ICAM-1, and TGF-β1, and reduced pro-angiogenic markers like VEGF and HIF-1α. It also lowered the expression of oncogenic proteins, including *STAT3* and MMP-9, while promoting apoptosis through increased levels of pro-apoptotic markers such as Bax and cleaved caspase-3 and decreased levels of anti-apoptotic protein BCL-2. These findings suggest that vinpocetine, a safe nootropic drug, holds promise as a hepatoprotective agent and a potential option for the prevention of HCC development by targeting multiple oncogenic pathways simultaneously.

## Materials and methods

### Assay of cell proliferation and inhibition rate

The HepG2 cell line, identified as HB-8065 and obtained from the American Type Culture Collection (ATCC), was acquired for the study. These cells were cultured in a cell incubator with a 5% CO_2_ atmosphere at a temperature of 37°C. The culture medium consisted of Dulbecco’s modified Eagle medium (DMEM) (Thermo Fisher Scientific, Waltham, MA, USA) supplemented with 10% fetal bovine serum (FBS) (Gibco, Thermo Fisher Scientific, Waltham, MA, USA), 100 μg/ml of streptomycin (Sigma-Aldrich, St. Louis, MO, USA), 100 IU/ml of penicillin (Sigma-Aldrich), and 2 mM of glutamine (Sigma-Aldrich). Passaging was carried out every 2 days, and only cells in the exponential growth phase were used. Following this, the cells were subcultured into a 96-well plate, with each well containing 2 × 10^4^ cells in 100 μl of culture medium supplemented with 0.5% dimethyl sulfoxide (DMSO) (Sigma-Aldrich). The cells were maintained in a controlled environment at 37°C with a specific humidity level and a gas mixture of 5% CO_2_, 74% N_2_, and 21% O_2_. After allowing the cells to adhere for 24 hours, the existing culture medium in each well was replaced with fresh medium containing varying concentrations of vinpocetine (0, 5, 10, 20, 40, or 80 μM). The cells were then further incubated for 24, 48, or 72 hours. To assess the cytotoxicity, an assay was performed by adding 20 μl of 3-(4,5-Dimethylthiazol-2-yl)-2,5-diphenyltetrazolium bromide (MTT) solution (5 mg/ml) (Sigma-Aldrich) to each well and maintaining the cells at 37°C for an additional 4 hours. Subsequently, the culture medium was replaced with 50 μl of DMSO to aid in dissolving the purple formazan crystals that were otherwise insoluble. To ensure complete dissolution, the cells were then subjected to an additional 30-minute incubation with agitation on a shaker. To evaluate cell proliferation, optical density (OD) measurements were carried out at a wavelength of 490 nm using a Biotek plate reader sourced from Winooski, Vermont, USA. The percentage of viable cells was determined by comparing them to the untreated group and adjusting the results accordingly. All experiments were repeated in triplicates. It is worth noting that 15, 30, and 60 μM of vinpocetine were examined previously in MDA-MB-231 cell line [22]. Additionally, vinpocetine (5–50 μM; 7 hours; VSMCs, HUVECs, A549 cells and RAW264.7 cells) potently inhibited TNF-α-induced NF-κB-dependent transcriptional activity in a dose-dependent manner with an approximate IC_50_ value of 25 μM [25].

### Determination of the LDH release

The evaluation of LDH activity in HepG2 cells exposed to different concentrations of vinpocetine (0, 5, 10, 20, 40, or 80 μM) for a 24-hour duration was carried out using a commercially available kit obtained from Sigma-Aldrich; MAK066. After the incubation period, the culture media were collected and then subjected to centrifugation at 300 × g for 5 minutes. Supernatant was used to detect LDH levels in the media. To determine LDH levels in the entire cell lysate, the rest of cells were treated with a 10 mM phosphate buffer at pH 7.4, containing 1% Triton X-100 (w/v). The cell suspension was homogenized by pushing it through a 27-gauge needle, followed by centrifugation at 800 x g and 4°C for 10 minutes. The resulting supernatant, which represented the entire cell lysate, was transferred to a fresh tube. The levels of LDH in the culture medium and the complete cell lysate were assessed at a wavelength of 450 nm. To calculate the percentage of LDH released into the culture medium, the following formula was applied: Media Released LDH % = (LDH in the culture medium) / (LDH in the culture medium + LDH in the whole-cell lysate) x 100. Each concentration was tested in triplicate for accuracy and consistency.

### Determination of CCND1, cleaved caspase-3, and caspase-1 activity in HepG2 cells

HepG2 cells were seeded into individual wells of a 6-well plate at a concentration of 5 x 10^5^ cells per well. They were cultured in DMEM containing 0.5% DMSO and subsequently exposed to vinpocetine at concentrations of 10 and 20 μM, following the conditions mentioned previously. After a 24-hour incubation period, the cells were harvested and lysed using a cell lysis buffer that included protease inhibitors. The lysates were then centrifuged according to the manufacturer’s instructions, and the resulting supernatants were collected. To determine the protein concentrations in these supernatants, a BCA assay kit was utilized. For assessing the protein levels of CCND1, an ELISA kit from abcam located in Cambridge, UK, was employed (ab214571). The OD was measured at 450 nm, with an intra-assay CV% of ˂ 5% and an inter-assay CV of ˂ 7%. The protein levels of cleaved caspase-3 (KM300) and the activity of caspase-1 (K111-25) were assessed using ELISA kits sourced from R&D Systems in Minneapolis, MN, USA. The OD was measured at 450 nm, with an intra-assay CV of ˂ 4.16% and an inter-assay CV of ˂ 6.16% for cleaved caspase-3. For caspase-1 activity, the OD was measured at 405 nm, with both intra-assay and inter-assay CVs of ˂ 10%. The levels of expression for cleaved caspase-3 and the levels of caspase-1 activity were quantified in terms of fold changes relative to the group treated with 0 μM of vinpocetine. Each experiment was conducted in triplicate to ensure consistency of the results.

### Animals

The study involved male adult Sprague-Dawley rats procured from Theodor Bilharz Research Institute, Egypt. These rats were 8 weeks old and had an average weight of 200 ± 15 g. They were housed under standard environmental conditions, which included maintaining a relative humidity of 50 ± 10%, a temperature of 22 ± 2°C, and a 12-hour light/dark cycle. Before commencing any experimental procedures, the rats were given a two-week period to acclimate to their new environment.

### Study protocol

In this study, chemicals and medications were prepared for administration, with DENA sourced from Sigma-Aldrich and diluted in saline for intraperitoneal injection at a dosage of 100 mg/kg/week for 12 weeks, with slight modifications to previously established protocols [26, 27]. vinpocetine, obtained from Sigma-Aldrich, was freshly prepared and administered via gavage in a 0.5% carboxymethyl cellulose (CMC) solution at a dosage of 20 mg/kg once daily. The rats were randomly categorized into four groups: a normal control group, a vinpocetine (VPCTN) control group, an DENA group exposed to DENA, and an DENA/VPCTN group receiving both DENA and vinpocetine treatment. After twelve weeks of treatment, the rats were euthanized through decapitation following anesthesia induction, which consisted of a mixture of 12.5 mg/kg Xylazine and 87.5 mg/kg Ketamine. The treatment of the rats adhered to the guidelines established by the Research Ethics Committee at Delta University, and it was carried out with approval under the reference number FPDUST23121/4. Including a vinpocetine control group in this study is essential to accurately assess the effects of vinpocetine itself. It serves as a crucial drug-only control, enhancing the reliability of the study’s findings. The key role of the normal group is to provide baseline, untreated data that all other treatment groups can be compared to. Both the control and drug control groups received the vehicles of both vinpocetine and DENA following the same regimen. In the DENA/VPCTN group, rats were administered vinpocetine on the day of DENA administration, with approximately one hour separating the two regimens. On subsequent days, vinpocetine was administered regularly at the same time.

### Rationale for selecting the vinpocetine dosage

The vinpocetine dosage regimen in our study was chosen based on a comprehensive review of previously published studies [28–39], which demonstrated the effective use of vinpocetine in various models, including liver fibrosis, neuroprotection, and anti-inflammatory effects. These studies provided a solid foundation for selecting an appropriate dose for our HCC model. The dosage aligns closely with the established therapeutic ranges from the literature, ensuring safety and efficacy.

### Histological study

The liver tissue samples were initially fixed in formalin for preservation. Afterward, they were embedded in paraffin blocks and then sliced into sections that were 4 micrometers thick. These sections were then subjected to conventional procedures for H&E staining [40, 41] or Sirius red staining. After completing the staining procedure, we used a microscope to examine the liver sections. To evaluate inflammation, we employed the modified-Ishak necro-inflammation index for quantification purposes [42]. The level of inflammation was assessed by summing the scores obtained from four categories of microscopic features, with higher scores indicating a more substantial deviation from the normal control condition. For the assessment of fibrosis area in Sirius red stained sections, we utilized ImageJ software version 1.54f.

### Calculation of liver weight index

To determine the proportion of liver weight relative to the body weight of the animals, we calculated the liver weight index using the following formula: Liver weight index = Liver weight (g) / Body weight (g).

### Determination of oxidative stress parameters

The tissue homogenate was prepared by first perfusing the liver tissue with a PBS solution (pH 7.4) containing 0.16 mg/ml heparin to remove any red blood cells and clots. The tissue was then homogenized in 5–10 ml of cold buffer (50 mM potassium phosphate, pH 7.5) per g of liver, followed by centrifugation at 4000 rpm for 15 minutes. The supernatant was removed for malondialdehyde (MDA), super oxide dismutase (SOD), and glutathione (GSH) spectrophotometric assay using Biodiagnostic kits sourced from Giza, Egypt; MD2529, SD2521, and GR2511, respectively. To detect the presence of reactive oxygen species (ROS) in the tissues, we employed a method that had been previously described in other sources [43]. Briefly, liver tissue samples (200 mg each) were homogenized in ice-cold 40 mM Tris-HCl buffer (pH 7.4) at a tissue-to-buffer ratio of 1:10 (w/v). From each homogenate, 100 μL aliquots were taken and mixed with 1 mL of Tris-HCl buffer. Next, 5 μL of 20,70-dichlorofluorescein diacetate (10 μM) was added as a fluorescent probe. The mixtures were incubated at 37°C for 30 minutes. After incubation, fluorescence intensity was measured using a fluorescence microplate reader (Tecan, Mainz, Germany), with excitation and emission wavelengths set to 485 nm and 525 nm, respectively. The experiments were conducted in triplicates, ensuring reliability.

### Determination of liver function enzymes

We measured the serum activity of alanine transaminase (ALT), aspartate aminotransferase (AST), and gamma-glutamyl transferase (γGT) enzymes using assays and kits acquired from Biodiagnostic. These measurements were carried out in accordance with the manufacturer’s instructions.

### Immunolabeling of Ki-67, VEGF, and caspase-3

Sections measuring 4 μm-thick were obtained from tissue blocks that had been fixed in formalin and embedded in paraffin. These sections were then attached to microscope slides coated with poly-L-lysine. The paraffin-embedded sections underwent a deparaffinization process using xylene and were gradually rehydrated by passing through a gradient of ethanol to water. Following this, antigen retrieval was performed in a sodium citrate buffer with a pH of 6.0, at which point the slides were allowed to cool for 15 minutes. Subsequently, the slides were subjected to three 5-minute washes with Tris-buffered saline (TBS) to remove excess materials. To reduce any inherent peroxidase activity, the slides were immersed in a 3% H_2_O_2_ solution in methanol for 10 minutes. Following this step, a power block was applied for 10 minutes using the Ultra Vision Plus Detection System from Thermo Scientific to prevent nonspecific binding. The slides were then rinsed with TBS. Following this, the slides were placed in a humidified chamber and allowed to incubate overnight at a temperature of 4°C with the primary antibody. After incubation, the slides were washed once again with TBS. The sections were then subjected to a 20-minute incubation with a biotinylated goat antipolyvalent secondary antibody, which was provided by the Ultra Vision Plus Detection System by Thermo Scientific. After this incubation, the sections were rinsed with TBS once more. Subsequently, the sections underwent a 30-minute incubation with streptavidin peroxidase plus, also from the Ultra Vision Plus Detection System by Thermo Scientific. Following another wash with TBS, the sections were developed using a 3,30-diaminobenzidine (DAB) solution provided by the Ultra Vision Plus Detection System until they exhibited a brown coloration. Finally, Mayer’s hematoxylin was used to counterstain the sections, and they were mounted with mounting media for observation under a light microscope (Olympus CX23, Tokyo, Japan).

In this study, primary antibodies were utilized, which included an anti-rat rabbit polyclonal Ki-67 antibody (diluted at 1:100; ABclonal, Inc., Woburn, MA, USA), an anti-rat rabbit polyclonal VEGF antibody (diluted at 1:300, Neomarkers, Fremont CA, USA), and an anti-rat rabbit polyclonal caspase-3 antibody (diluted at 1:100, ABclonal, Inc.). To evaluate the expression of these target proteins in the tissue, the percentage of positive cells was determined by counting a total of 1000 cells across 10 different high-power fields (HPF).

### ELISA measurements of the parameters assessed in the *in vivo* protocol

The evaluation of p65 DNA binding activity was conducted using a kit from Abcam (ab133112). To perform this assay, a specific double-stranded DNA sequence containing the consensus binding site for NF-κB p65 (5′-GGGACTTTCC-3′) was immobilized onto a 96-well plate. Active NF-κB p65 present in the nuclear extract specifically binds to this oligonucleotide. Detection of NF-κB p65 was achieved using a primary antibody that recognizes the specific epitope of NF-κB p65, which is accessible only when the protein is activated and bound to its target DNA. A secondary antibody conjugated with HRP provided a sensitive colorimetric readout, which was measured at an OD of 450 nm, with both intra-assay and inter-assay CVs of ˂ 10%. To prepare the nuclear extracts, a nuclear extraction kit (ab113474) was used as per the provided instructions. The protein concentration of the extract was determined before dividing it into aliquots, and these extracts were subsequently stored at -80°C.

The measurement of IL-6 in liver homogenates followed the instructions provided by R&D Systems, Minneapolis, MN, USA (R6000B). The OD was measured at 450 nm, with an intra-assay CV of ˂ 4.5% and an inter-assay CV of ˂ 8.3%. Levels of monocyte chemoattractant protein-1 (MCP-1), intercellular adhesion molecule-1 (ICAM-1) and transforming growth factor-beta (TGF-β) were assessed in liver homogenates using kits supplied by MyBioSource Inc. (San Diego, CA, USA); MBS9501937, MBS451470, and MBS824788, respectively. The OD was measured at 450 nm, with both intra-assay and inter-assay CVs of ˂ 10% for MCP-1. For ICAM-1, the OD was measured at 450 nm, with an intra-assay CV of ˂ 10% and an inter-assay CV of ˂ 12%. For TGF-β, the OD was measured at 450 nm, with both intra-assay and inter-assay CVs of ˂ 10%.

For the measurement of VEGF and matrix metalloproteinase-9 (MMP-9) levels in liver tissue homogenates, kits from CUSABIO (Wuhan, China) were utilized; CSB-E04757r and CSB-E08008r, respectively. The OD was measured at 450 nm, with an intra-assay CV of ˂ 8% and an inter-assay CV of ˂ 10% for both VEGF and MMP-9. The assessment of hypoxia-inducible factor 1-alpha (HIF-1α) in tissue homogenate was performed following the instructions provided by MyBioSource; MBS028091. The OD was measured at 450 nm, with both intra-assay and inter-assay CVs of ˂ 15%.

The quantification of CCND1 levels was conducted using a kit provided by Novus Biologicals, located in Colorado, USA; NBP2-75102. The OD was measured at 450 nm, with an intra-assay CV of ˂ 4.77% and an inter-assay CV of ˂ 4.84%. Similarly, levels of BCL-2 were assessed using a kit also supplied by Novus Biologicals; NBP2-69947. The OD was measured at 450 nm, with an intra-assay CV of ˂ 4.92% and an inter-assay CV of ˂ 5.33%.

For the determination of Bax and cleaved caspase-3 levels, kits were furnished by MyBioSource; MBS935667 and MBS7244630, respectively. The OD was measured at 450 nm, with an intra-assay CV of ˂ 8% and an inter-assay CV of ˂ 10% for Bax. For cleaved caspase-3, the OD was measured at 450 nm, with both intra-assay and inter-assay CVs of ˂ 10%. P-STAT3 was assessed following instructions by RayBiotech (Norcross, GA); PEL-Stat3-Y705. The serum levels of AFP were quantified through an ELISA (R&D Systems; AF1369). The OD was measured at 450 nm, with both intra-assay and inter-assay CVs of ˂ 10% for both P-STAT3 and AFP.

The measurements were carried out following the instructions provided by each manufacturer. To determine the protein concentration in the extracted samples, a BCA (bicinchoninic acid) protein assay reagent kit was employed, and this kit was purchased from Thermo Fisher Scientific Inc. (Rockford, USA).

### Quantitative reverse transcription polymerase chain reaction (qRT-PCR)

To isolate total RNA, we used the RNeasy Mini kit provided by Qiagen, located in Hilden, Germany. This RNA extraction was performed under conditions free from RNase contamination, following the manufacturer’s instructions. To assess the concentration and purity of the extracted RNA, we measured its absorbance at 260 nm using a NanoDrop 2000 spectrophotometer from Thermo Fisher Scientific. For the reverse transcription of 2 μg of total RNA into complementary DNA (cDNA), we employed the Quantiscript reverse transcriptase kit from Qiagen in a 20 μl reaction volume. In the subsequent quantitative reverse transcription-polymerase chain reaction (qRT-PCR), we utilized a Rotor-Gene Q thermocycler from Qiagen, along with SYBR Green PCR Master Mix, also provided by Qiagen. Each reaction was carried out in triplicate, and the expression levels were normalized to GAPDH. The primer sequences utilized for RT-PCR are detailed in Table 1. The relative gene expression was determined using the comparative cycle threshold (Ct) method (2^(-ΔΔCT)^).

**Table 1:** Primer sequences.

Gene	GenBank accession number	Forward primer	Reverse Primer	Amplicon size (bp)
*VEGF*	NM_031836.3	5′-GCTCTCTTGGGTGCACTGGAC-3′	5′-ACGGCAATAGCTGCGCTGGTA-3′	145
*STAT3*	NM_012747.3	5′-AGAGGCGGCAGCAGATAGC-3′	5′-TTGTTGGCGGGTCTGAAGTT-3′	110
*GAPDH*	NM_017008.4	5′-GGTCATCCCTGAGCTGAACG-3′	5′-TCCGTTGTCATACCAGGAAAT-3′	295

### Survival analysis and statistical methods

We used Kaplan-Meier survival plots to compare the survival rates between the rats in the DENA/VPCTN group and those in the DENA group. Statistical analysis was performed using GraphPad Prism version 9. Group comparisons were conducted using one-way ANOVA, followed by Tukey’s post hoc test for datasets with a normal distribution. For datasets that did not follow a normal distribution, we applied the Mann-Whitney test for comparisons between two independent non-parametric data groups (histological score). The data are presented as either mean ± standard deviation (SD) for parametric data or median ± interquartile range (IQR) for non-parametric data. We considered statistical significance to be reached when p-values were equal or below 0.05, with values below this threshold considered statistically significant.

## Results

### Effect of vinpocetine on HepG2 cell proliferation and LDH activity

**Fig 1:**
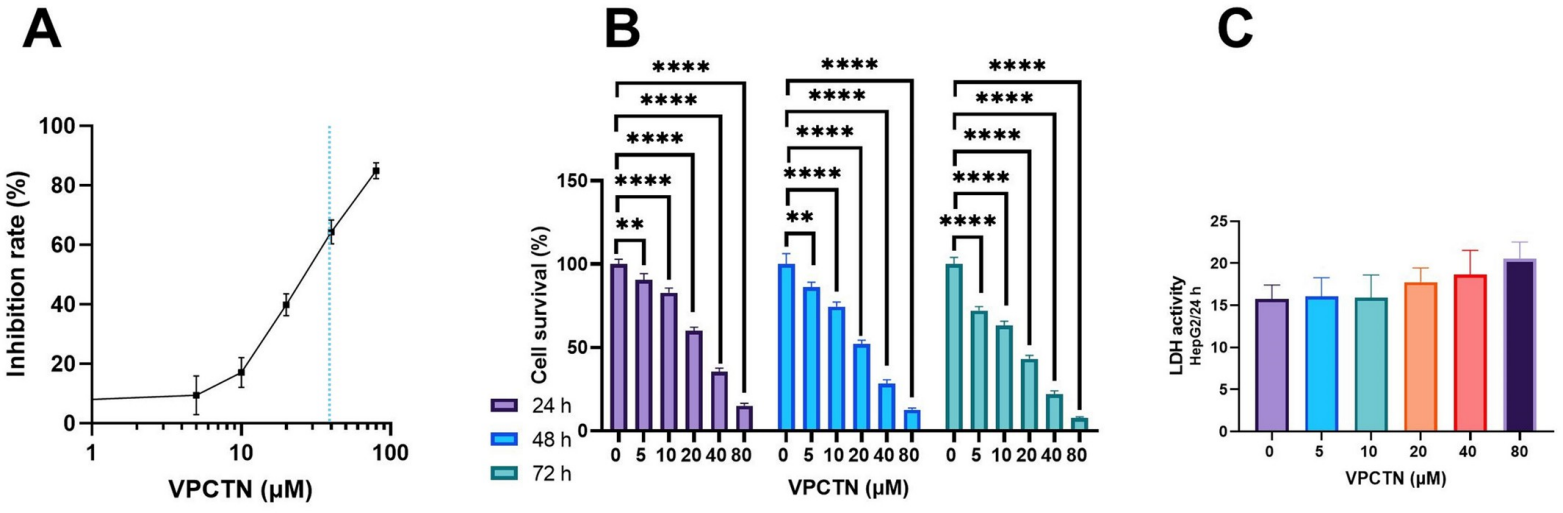
Impact of progressive vinpocetine dosage escalation on HepG2 cell viability. (A) a decline in HepG2 cell viability with escalating vinpocetine dosage and consequential augmentation in cell inhibition rates. (B) cellular proliferation at varying vinpocetine concentrations over 24, 48, 72-hour intervals. (C) LDH Activity at a gradual elevation of vinpocetine concentration. Data are presented as mean ± SD (n = 3). Statistical analysis was performed using ordinary one-way ANOVA, followed by Tukey’s posthoc test. ****p < 0.0001; **p < 0.01.

Vinpocetine dosage escalation has demonstrated a consequential augmentation in the inhibition rate of HepG2 cells (Fig 1A). Using simple linear regression analysis, vinpocetine displayed an IC_50_ value of 39 μM in the experimental set of HepG2 cells conducted for 24 hours. The IC_50_ value is indicated by the dashed line. When administered in increasing concentrations over distinct time intervals, specifically 24, 48, and 72 hours, the cell survival rate consistently declined (Fig 1B). In addition, although there was a slight rise in LDH activity in response to the gradual elevation of vinpocetine concentration, it is important to note that no statistically significant differences were observed across the various concentrations of vinpocetine (Fig 1C).

### Effect of vinpocetine on CCND1, cleaved caspase-3, and caspase-1 activity in HepG2 cells

**Fig 2:**
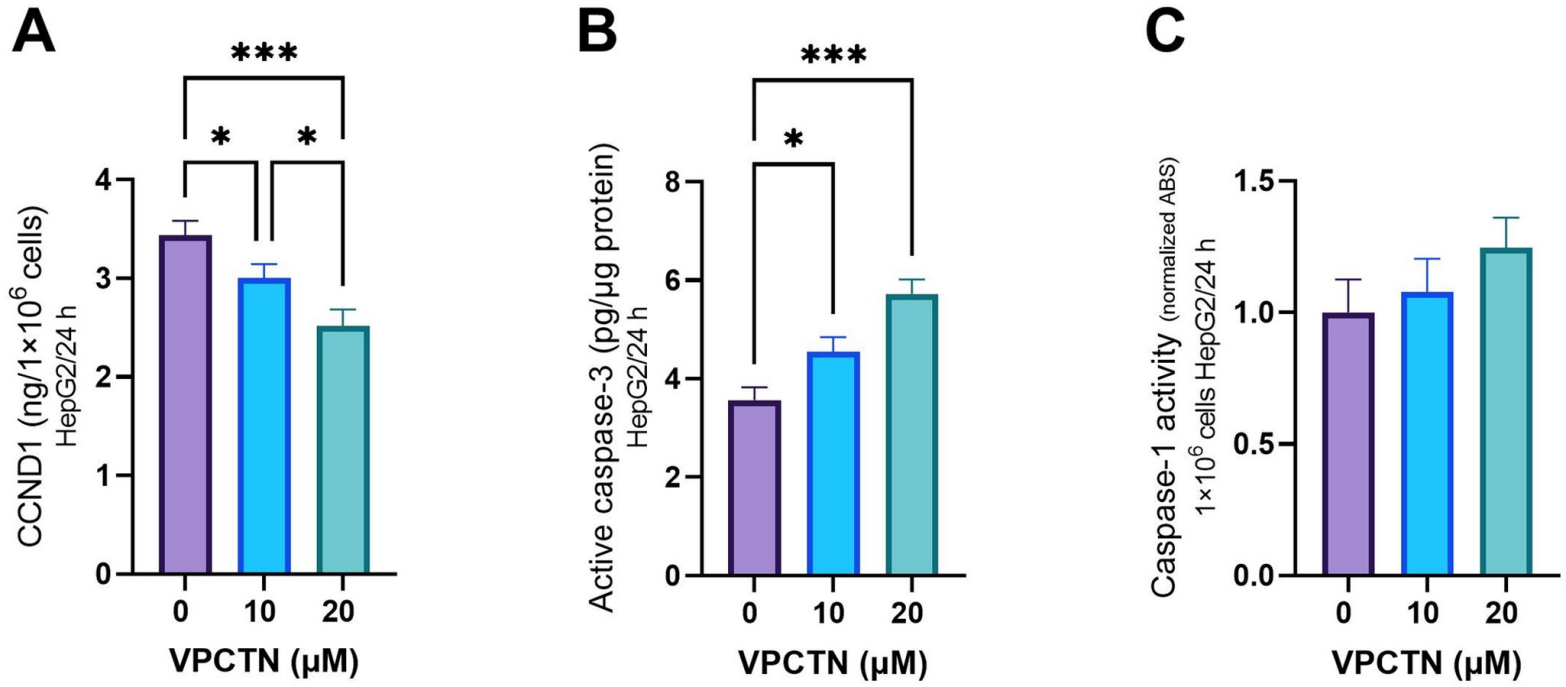
Effects of vinpocetine on CCND1 (A), Cleaved Caspase-3 (B), and Caspase-1 Activity (C) Levels in HepG2 Cells. Data are presented as mean ± SD (n = 6). Statistical analysis was performed using ordinary one-way ANOVA, followed by Tukey’s posthoc test. ***p < 0.001; *p < 0.05.

Upon the introduction of varying concentrations of vinpocetine (10 and 20 μM), a notable and statistically significant reduction in CCND1 levels was observed in HepG2 cells within a 24-hour timeframe, as illustrated in Fig 2A. This compelling finding underscores the potential of vinpocetine to influence the expression of CCND1. Furthermore, when examining the impact of vinpocetine at 10 and 20 μM concentrations within the same cellular context, a significant increase in cleaved caspase-3 levels was observed in HepG2 cells within a span of 24 hours compared to their untreated counterparts (Fig 2B). Meanwhile, the concentrations of vinpocetine at 10 and 20 μM did not yield any statistically significant increases in caspase-1 activity levels within the 24-hour window in HepG2 cells, as demonstrated in Fig 2C.

### Histopathological evaluation

**Fig 3:**
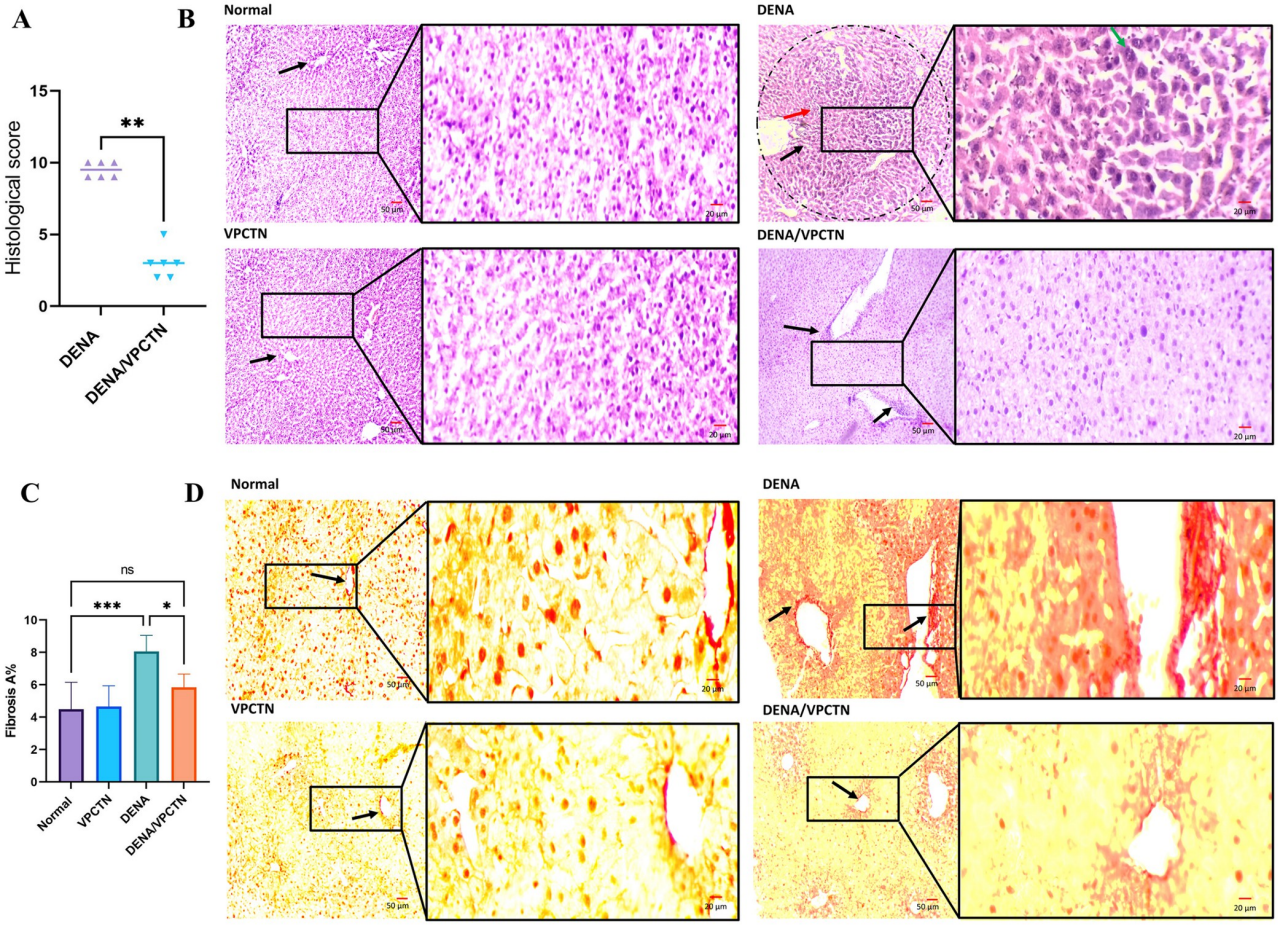
Histological score (A); liver ultrastructure examination in different experimental groups stained with H&E stain (B); Fibrosis area % as quantified using ImageJ software (C); Sirius Red-stained liver sections (D). scale bar = 50 μm. Data were analyzed with the Mann-Whitney test and are expressed as the median ± IQR for histological score (n = 6). Data were analyzed using ordinary one-way ANOVA, followed by Tukey’s posthoc test and are presented as mean ± SD for fibrosis area % (n = 6). ****, p < 0.0001; ***, p < 0.001; **, p < 0.01; *, p < 0.05.

In **panel B**, as depicted in Fig 3, the liver sections from both the Normal and VPCTN groups show a typical liver structure (black arrows). In contrast, the section from the DENA group exhibits area of high cellular proliferation (encircled) that disrupt the normal liver structure. This area is marked by irregularly enlarged nuclei (green arrow, high magnification inset), nuclei with increased chromatin content, and the presence of inflammatory cells (red arrow) and vacuolations (black arrow). On the other hand, the sections from the DENA/VPCTN group do not display these neoplastic changes. The hepatocytes and their nuclei maintain a more regular and characteristic appearance, although there is still some inflammatory cell infiltration around veins in this group (black arrows). Additionally, there is a significant reduction in the inflammation score in the DENA/VPCTN group when compared to the DENA group (**panel A**). On the other hand, as depicted in **panel D**, the liver sections from both the Normal and VPCTN groups show normal collagen deposition (black arrows). In contrast, the sections from the DENA group exhibit high fibrotic tissue deposition (black arrows). Conversely, the sections from the DENA/VPCTN group display attenuated fibrotic tissue deposition (black arrow). Additionally, there is a significant reduction in the fibrosis A% in the DENA/VPCTN group when compared to the DENA group as calculated by the ImageJ software (**panel C**).

### Vinpocetine mitigated liver weight index and oxidative stress markers, and improved antioxidant defense and liver function in DENA-intoxicated rats

**Fig 4:**
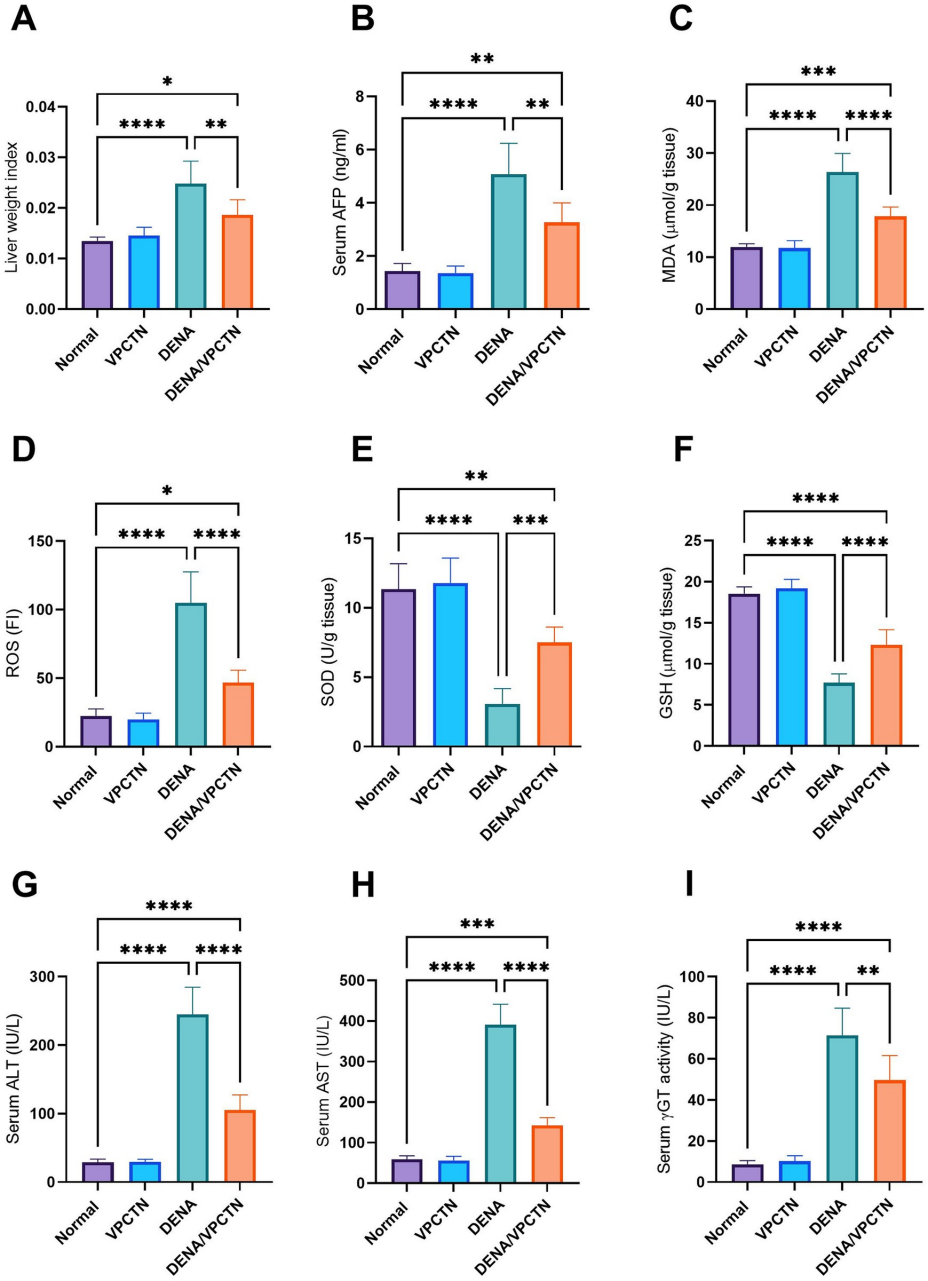
Vinpocetine mitigated liver weight index (A), AFP (B), MDA (C) and ROS (D) and improved SOD (E) and GSH (F). It also improved liver function by mitigating ALT (G), AST (H), γGT (I) in DENA-intoxicated rats. Data are presented as mean ± SD (n = 6). Statistical analysis was performed using ordinary one-way ANOVA, followed by Tukey’s posthoc test. ****, p < 0.0001; ***, p < 0.001; **, p < 0.01; *, p < 0.05.

The administration of DENA resulted in a statistically significant elevation in the liver weight index compared to untreated, healthy rats. However, when vinpocetine was administered to DENA-treated rats, a significant decrease in the liver weight index was observed (Fig 4A). DENA administration caused a significant increase in the levels of AFP, MDA, and ROS compared to the untreated, normal group (Fig 4B–4D, respectively). Remarkably, administering vinpocetine to DENA-treated rats resulted in a significant reduction in these levels. Furthermore, DENA administration resulted in a notable decline in the levels of SOD and GSH. However, vinpocetine treatment of DENA-treated rats led to a marked increase in SOD and GSH levels (Fig 4E and 4F, respectively). DENA treatment significantly increased the levels of ALT, AST, and γGT compared to untreated healthy control rats (Fig 4G–4I, respectively). Interestingly, vinpocetine administration to DENA-treated rats resulted in a marked reduction in the levels of ALT, AST, and γGT.

### Vinpocetine reduced Ki-67 immunoexpression and modulated angiogenic process induced by DENA exposure in rats

**Fig 5:**
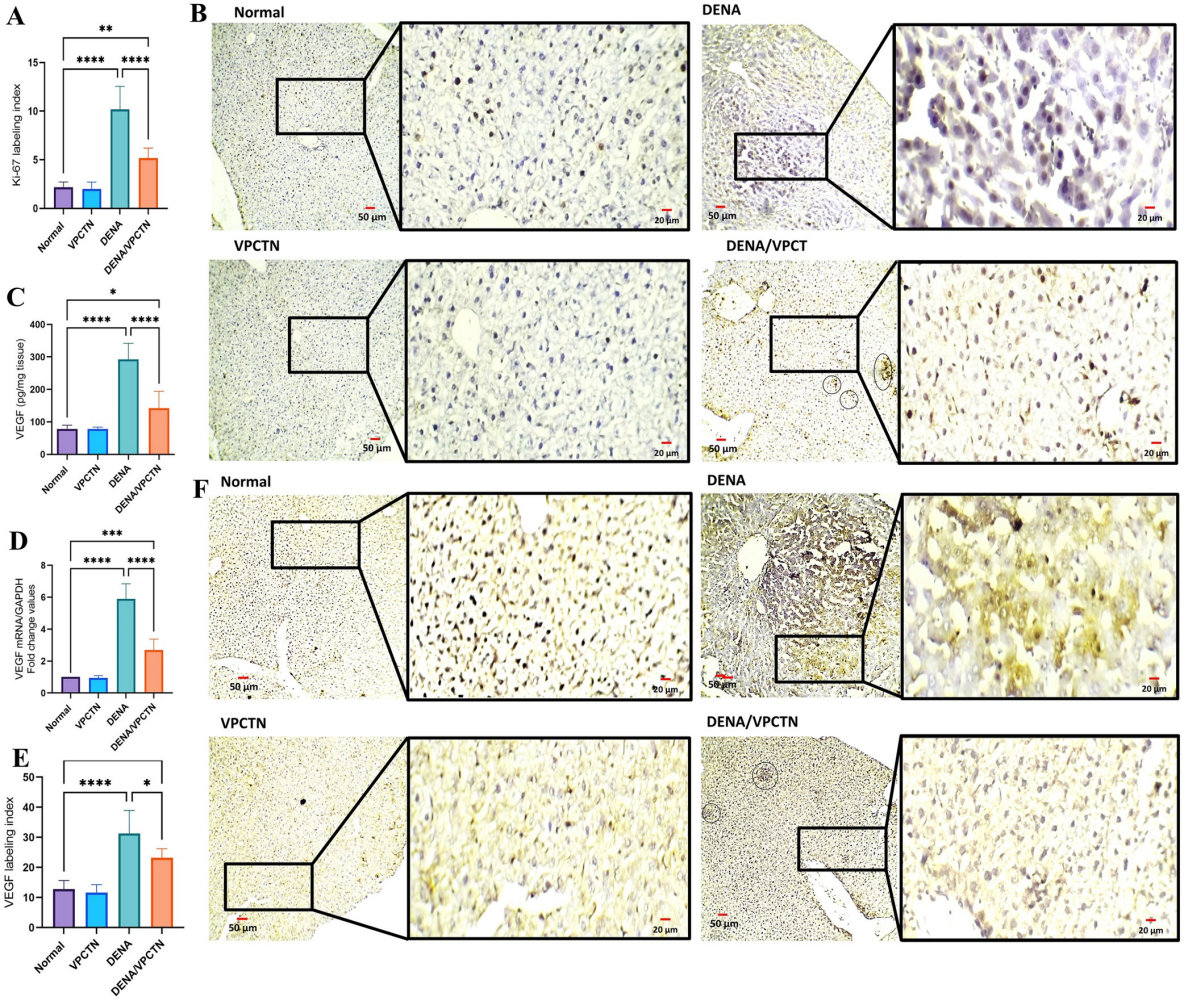
Impact of vinpocetine on Ki-67 labeling index (A) and tissue expression (B). VEGF hepatic level (C) and mRNA expression (D) confirm the findings of VEGF labeling index (E) and immunohistochemical labeling (F). Data are presented as mean ± SD (n = 6). Statistical analysis was performed using ordinary one-way ANOVA, followed by Tukey’s posthoc test. ****, p < 0.0001; ***, p < 0.001; **, p < 0.01; *, p < 0.05.

As shown in Fig 5, DENA administration caused a marked increase in the expression of Ki-67, a well-known cellular nuclear marker for proliferation (**B**). Quantification of its labeling index confirmed the immunohistochemical examination (**A**). On the other hand, when vinpocetine was added, we noticed a significant decrease in Ki-67 expression in comparison to the DENA group. In response to DENA exposure, both mRNA expression and cytoplasmic protein expression VEGF exhibited a marked elevation compared to healthy, untreated rats (**C and D**, respectively). Remarkably, the intervention with vinpocetine, when administered to rats treated with DENA, resulted in a significant reduction in both the protein and mRNA expression of VEGF. These findings align with those revealed after the assessment of the VEGF labeling index (**E**) subsequent to immunohistochemical staining of liver tissue (**F**).

### Impact of vinpocetine on DENA-induced oncogenic molecular alterations in rats

**Fig 6:**
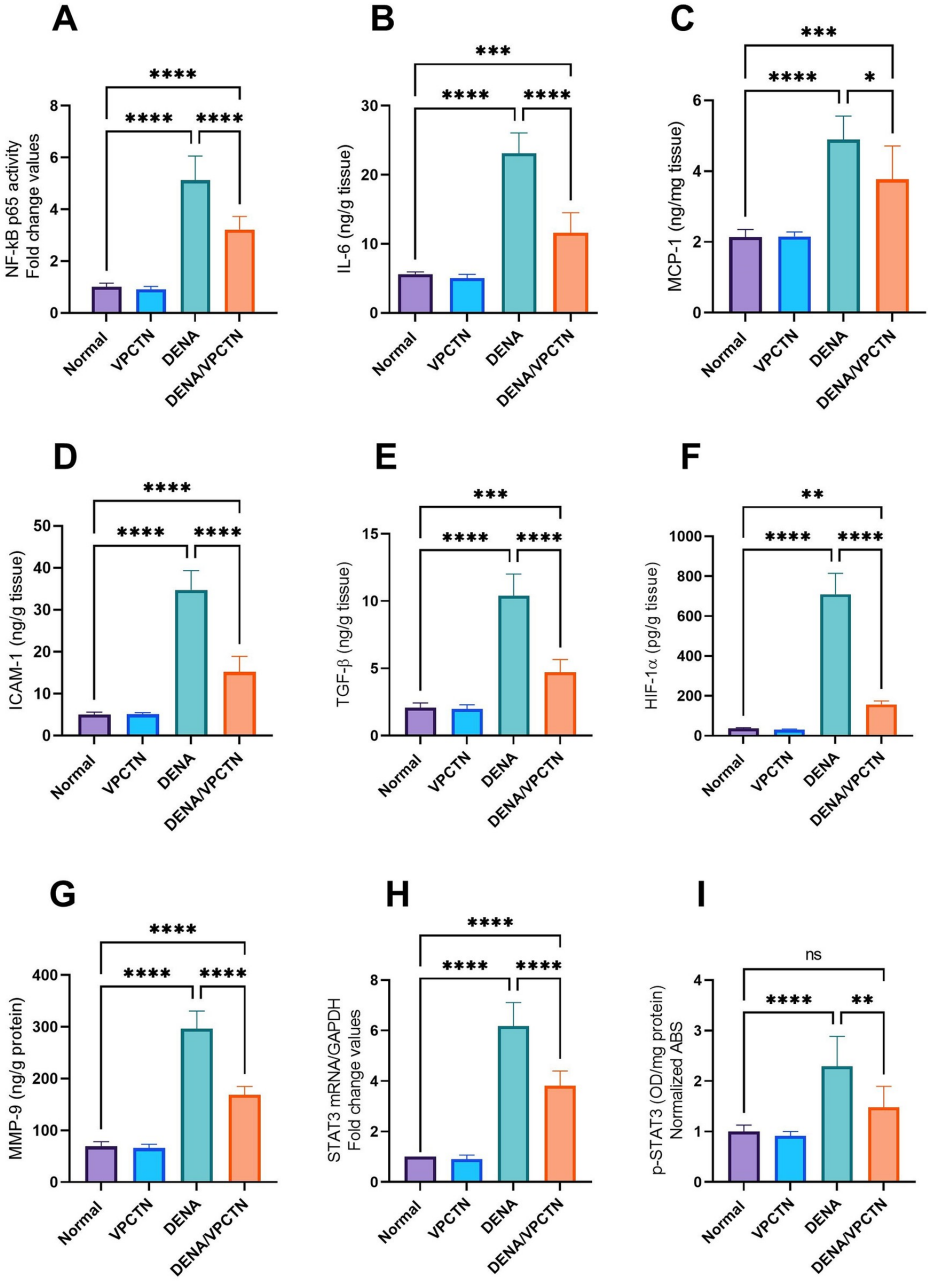
Effects of vinpocetine on DENA-induced oncogenic molecular alterations in rats. Panels (A) through (I) depict changes in NF-κB p65 DNA-binding activity, IL-6 levels, MCP-1 expression, ICAM-1 expression, TGF-β expression, HIF-1α levels, MMP-9 levels, *STAT3* mRNA levels, and p-STAT3 levels, respectively. Data are presented as mean ± SD (n = 6). Statistical analysis was performed using ordinary one-way ANOVA, followed by Tukey’s posthoc test. ****, p < 0.0001; ***, p < 0.001; **, p < 0.01; *, p < 0.05.

The administration of DENA significantly increased nuclear NF-κB p65 DNA-binding activity and IL-6 levels, as illustrated in Fig 6A and 6B. These elevations were significantly reduced in response to vinpocetine treatment. Additionally, DENA elevated the hepatic expression of MCP-1, ICAM-1, and TGF-β, as depicted in Fig 6C–6E, respectively. Rats exposed to DENA showed a substantial rise in these molecules compared to healthy, untreated counterparts. Conversely, vinpocetine intervention notably reduced the levels of MCP-1, ICAM-1, and TGF-β in DENA-treated rats. Furthermore, DENA administration led to significant increases in HIF-1α and MMP-9 levels (Fig 6F and 6G), which were significantly decreased following vinpocetine treatment. Similarly, DENA exposure resulted in elevated STAT3 mRNA and p-STAT3 levels (Fig 6H and 6I), which were significantly reduced with vinpocetine administration in DENA-treated rats.

### Effects of vinpocetine on DENA-induced alterations in CCND1, BCL-2, and Bax expression

**Fig 7:**
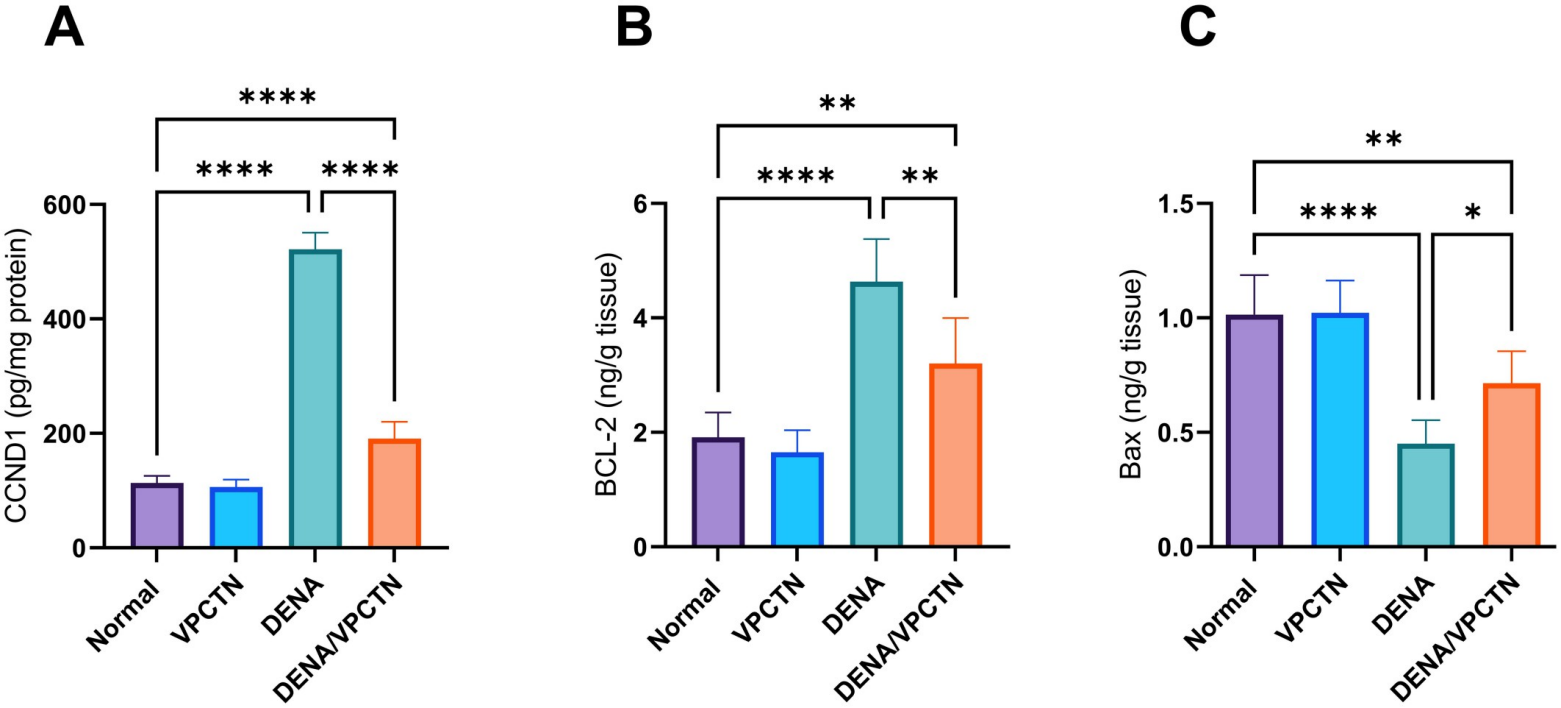
This figure demonstrates the changes in expression levels of CCND1 (A), BCL-2 (B), and Bax (C) following DENA administration and subsequent vinpocetine treatment in rat liver tissues. Data are presented as mean ± SD (n = 6). Statistical analysis was performed using ordinary one-way ANOVA, followed by Tukey’s posthoc test. ****p < 0.0001; **p < 0.01; *p < 0.05.

The expression of CCND1 was significantly affected by the administration of DENA (Fig 7A). Treatment with vinpocetine resulted in a substantial decrease in CCND1 levels compared to the DENA-exposed group. Additionally, DENA administration led to a statistically significant upregulation of BCL-2 levels and downregulation of Bax (Fig 7B and 7C, respectively). However, introducing vinpocetine into the experimental paradigm significantly reduced BCL-2 levels and significantly increased Bax levels compared to the DENA group.

### Effect of vinpocetine on caspase-3 immunoexpression and the levels of cleaved caspase-3 in DENA-intoxicated rats

**Fig 8:**
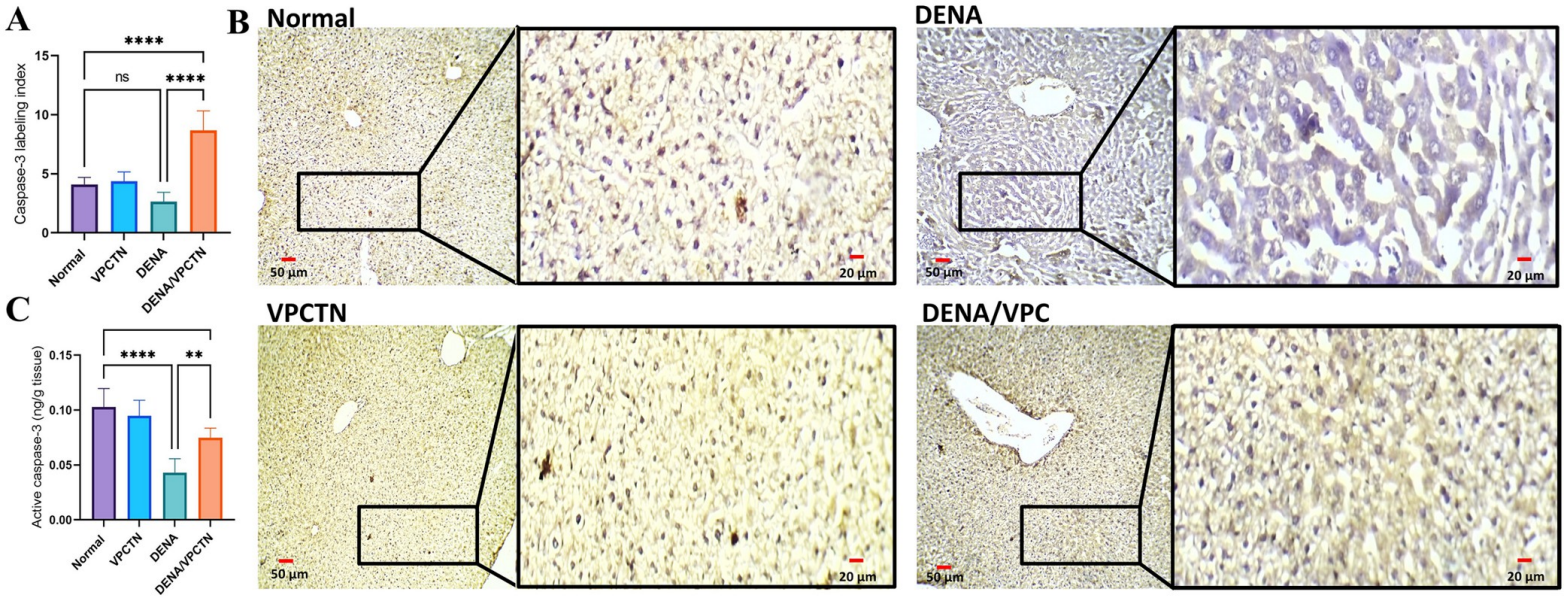
This figure illustrates the impact of vinpocetine treatment on caspase-3 labeling index (A) as determined by immunostaining (B) and cleaved caspase-3 levels (C) in DENA-exposed rats. Data are presented as mean ± SD (n = 6). Statistical analysis was performed using ordinary one-way ANOVA, followed by Tukey’s posthoc test. ****p < 0.0001; **p < 0.01.

As shown in Fig 8, Our findings revealed that the administration of DENA was associated with a reduction in the labeling index of caspase-3 (**A**), as observed through immunostaining and examination of stained liver specimens (**B**). Although this reduction did not reach statistical significance compared to the Normal group, it exhibited a notable trend toward significance. On the other hand, DENA administration led to a significant decrease in the levels of cleaved caspase-3 compared to those in the Normal group (**C**). Furthermore, the introduction of vinpocetine into the experimental setup resulted in a significant increase in cleaved caspase-3 levels in the cytoplasm.

### Effect on survival rate of DENA-exposed rats

**Fig 9:**
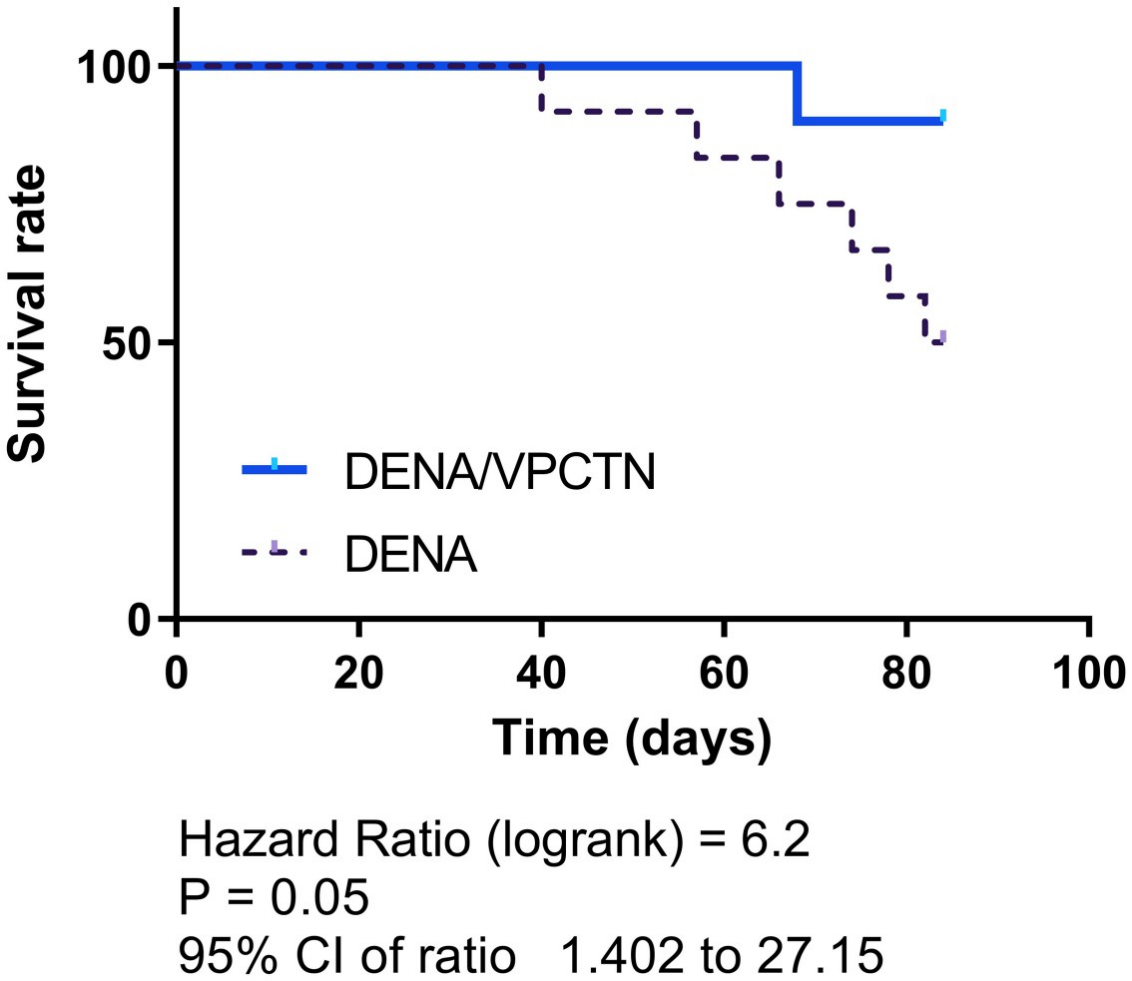
Vinpocetine improves survival rate in DENA-exposed rats. The administration of DENA led to a gradual decrease in rats’ survival over the experimental period. However, treatment with vinpocetine significantly enhanced the survival rate of rats exposed to DENA.

As depicted in Fig 9, the administration of DENA resulted in a gradual decrease in the survival rate of rats throughout the experimental period. However, when rats subjected to DENA were treated with vinpocetine, a significant enhancement in their survival rate was observed in comparison with that of the DENA group (p = 0.05; Hazard Ratio (log rank) = 6.2; 95% CI of ratio = 1.402 to 27.15).

### Summary of the relative effects of vinpocetine on various markers with implications

The relative proportional effects of vinpocetine on various markers assayed in our study are described in Table 2. The significant decrease in oncogenic markers, alongside an increase in pro-apoptotic markers, underscores vinpocetine’s multifaceted oncostatic potential.

**Table 2:** Summary of the relative proportional effects of vinpocetine on various markers with implications.

Marker	Tested In	Assay Method	Effect of DENA	Effect of Vinpocetine	Implications
HepG2 cell proliferation	In vitro	MTT assay	-	Significant decrease	Reduces cancer cell proliferation
LDH activity	In vitro	ELISA	-	No significant change	Indicates non-cytotoxicity through membrane damage
CCND1	In vitro	ELISA	-	Significant decrease	Inhibits cell cycle progression
Cleaved caspase-3	In vitro/In vivo	ELISA	Significant decrease	Significant increase	Promotes apoptosis
Caspase-1	In vitro	ELISA	-	No significant change	Indicates non-involvement in pyroptosis
Histopathological evaluation	In vivo	H&E staining	Significant abnormalities	Improvement	Indicates reduced neoplastic changes and inflammation
Fibrosis	In vivo	Sirius Red staining	Significant increase	Significant decrease	Reduces fibrotic tissue deposition
Liver weight index	In vivo	Measurement	Significant increase	Significant decrease	Indicates reduced liver hypertrophy and potential tumor size
AFP	In vivo	ELISA	Significant increase	Significant decrease	Indicates potential reduction in liver cancer markers
MDA	In vivo	Spectrophotometry	Significant increase	Significant decrease	Reduces oxidative stress
ROS	In vivo	Fluorimetry	Significant increase	Significant decrease	Reduces oxidative stress
SOD	In vivo	Spectrophotometry	Significant decrease	Significant increase	Enhances antioxidant defense
GSH	In vivo	Spectrophotometry	Significant decrease	Significant increase	Enhances antioxidant defense
ALT	In vivo	Spectrophotometry	Significant increase	Significant decrease	Indicates improved liver function
AST	In vivo	Spectrophotometry	Significant increase	Significant decrease	Indicates improved liver function
γGT	In vivo	Spectrophotometry	Significant increase	Significant decrease	Indicates improved liver function
Ki-67	In vivo	Immunohistochemistry	Significant increase	Significant decrease	Reduces cell proliferation
NF-κB p65	In vivo	ELISA	Significant increase	Significant decrease	Reduces inflammation and potentially inhibits tumor growth
IL-6	In vivo	ELISA	Significant increase	Significant decrease	Decreases inflammatory response
MCP-1	In vivo	ELISA	Significant increase	Significant decrease	Reduces inflammatory response
ICAM-1	In vivo	ELISA	Significant increase	Significant decrease	Reduces inflammatory response and potential metastasis
TGF-β1	In vivo	ELISA	Significant increase	Significant decrease	Reduces fibrosis
*VEGF*	In vivo	ELISA/qRT-PCR	Significant increase	Significant decrease	Reduces angiogenesis and tumor vascularization
HIF-1α	In vivo	ELISA	Significant increase	Significant decrease	Mitigates oncogenic conditions including angiogenesis
MMP-9	In vivo	ELISA	Significant increase	Significant decrease	Reduces tissue remodeling and invasiveness
*STAT3*	In vivo	qRT-PCR/ELISA	Significant increase	Significant decrease	Modulates signaling pathways related to tumor growth and metastasis
BCL-2	In vivo	ELISA	Significant increase	Significant decrease	Promotes apoptosis by reducing anti-apoptotic signals
Bax	In vivo	ELISA	Significant decrease	Significant increase	Enhances pro-apoptotic signals
Caspase-3 labeling index	In vivo	Immunohistochemistry	Significant decrease	Significant increase	Enhances apoptosis
Survival rate	In vivo	Kaplan-Meier analysis	Significant decrease	Significant increase	Indicates overall improved survival probability

## Discussion

The incidence of hepatocarcinogenesis is on the rise globally, primarily due to the escalating rates of chronic liver diseases such as cirrhosis and viral hepatitis [11, 44–46]. Existing treatments for HCC, a primary malignancy of the liver, while offering some benefits, are far from ideal in terms of efficacy and tolerability [47]. Patients often experience severe side effects, and the median survival rate remains disappointingly low [48]. Thus, there is a compelling need for novel drugs that not only improve survival rates but also enhance the quality of life. One effective strategy is the discovery of novel oncostatic agents to inhibit the development and progression of liver cancer.

The DENA model stands as a widely employed animal model for simulating HCC in rats. Researchers utilize this model to assess the effectiveness of anti-cancer drugs in countering liver malignant transformations [49]. The intricate molecular mechanisms underpinning DENA-induced hepatocarcinogenesis involve diverse cellular processes. DENA initiates DNA damage and mutations in liver cells, potentially activating oncogenes while hampering tumor suppressor genes, thereby promoting liver cancer development [50]. Additionally, DENA can induce hepatic inflammation, fostering a conducive microenvironment for liver cancer initiation and progression. Oxidative stress induced by DENA generates ROS in liver cells, further contributing to DNA damage [51]. Furthermore, DENA can stimulate liver cells proliferation and inhibit cellular death pathways, augmenting cancer cell survival and growth [52].

In the current study, our findings suggested a potential role of vinpocetine in the inactivation of *NF-κB* signaling. This effect is indicated by the vinpocetine’s capability to hinder the nuclear translocation of NF-κB, as evidenced by a decrease in DNA-binding activity detected in the nuclear extracts. It was previously reported that in the DENA-induced HCC animal model, there is a connection between NF-κB and cancer [53–55]. These studies demonstrated that *NF-κB* is intricately involved in HCC development within this model. Another study examined the impact of IKKβ deficiency, leading to the loss of *NF-κB* activity, in a DENA tumor model [56]. Considering these findings, it is reasonable to infer that NF-κB plays a significant role in the development of DENA-induced neoplastic changes. Additionally, the potential of vinpocetine to mitigate the effects of *NF-κB* and modulate its target genes suggests a promising avenue for intervention and regulation in the context of liver cancer.

In this current study, the administration of vinpocetine demonstrated antiangiogenic properties. These effects can be attributed, at least in part, to vinpocetine’s ability to inhibit the activity of *NF-κB*. In the context of cancer, the expression of NF-κB has been observed to exhibit a correlation with angiogenesis. This suggests that *NF-κB*’s activity plays a role in promoting cell survival by facilitating the formation of new blood vessels, which are characteristic features of this type of cancer. *NF-κB* exerts a significant influence on angiogenesis through various mechanisms including stimulating the expression of *VEGF* [57]. Moreover, it was proved that pyruvate kinase M2 (PKM2), a key player in pancreatic cancer cells, contributes to VEGF secretion by activating *NF-κB* transcription factors [58].

In addition to its role in angiogenesis, NF-κB activation and translocation plays a critical role in the regulation of IL-1β [20, 40, 59, 60] which has been linked to the activation of ICAM-1 [61]. ICAM-1 expression on host cells, such as liver cells, has been implicated in the formation of metastases in various organs [62]. Additionally, enhanced expression of TGF-β in cancer stimulates the activation of NF-κB [63] and promotes epithelial-mesenchymal transition (EMT) [64, 65]. Moreover, MCP-1, one of the key chemokines that regulate migration and infiltration of monocytes/macrophages plays a significant role in various hepatic disorders, including liver cancer [60]. Its influence extends to several critical pathways such as *NF-κB* with a particular relevance in the context of cancer [66]. Our study’s results highlight the potential of vinpocetine in influencing the mediators mentioned, especially those in which *NF-κB* signaling plays a pivotal role.

Another role of *NF-κB* is to suppress apoptosis by promoting the expression of genes that encode inhibitors of apoptosis. *Bax*, a pro-apoptotic protein within the *BCL-2* family, facilitates apoptosis by inducing mitochondrial outer membrane permeabilization [67]. Notably, *NF-κB* can regulate the activity of *Bax*, highlighting the intricate interplay between pro- and anti-apoptotic factors in determining the fate of a cell. BCL-2, an anti-apoptotic protein, acts by inhibiting apoptosis through the prevention of mitochondrial outer membrane permeabilization and the release of cytochrome c. The interaction between BCL-2 and Bax is central to the finely tuned regulation of apoptosis, wherein the balance between these two factors determines the cell’s fate [68, 69]. Caspase-3, on the other hand, serves as an executioner caspase, holding a pivotal role in the execution phase of apoptosis [70, 71]. Its primary responsibility lies in cleaving various cellular substrates, leading to programmed cell death [72]. Our findings revealed the vinpocetine potential to increase the levels of cleaved caspase-3 signifying its role in halting tumorigenesis. Coincident with apoptosis induction, vinpocetine exhibited proliferation suppression as revealed by immunostaining of Ki-67 and the levels of CCND1. These effects could also be attributed at least in part to the inhibition of the cell survival promoter *NF-κB*.

It was reported that zolmitriptan attenuates HCC via activation of caspase-mediated apoptosis [73]. This study on zolmitriptan primarily highlights its ability to activate caspase-mediated apoptosis as the central anti-HCC mechanism. It investigated the modulation of apoptosis-related proteins like BCL2, caspase-3, and caspase-9. In contrast, our study on vinpocetine demonstrates its multifaceted oncostatic effects by modulating several key oncogenic pathways involved in inflammation, angiogenesis, proliferation, and apoptosis. Notably, our study offers a more general view of vinpocetine’s hepatoprotective functions by also assessing oxidative stress markers, liver function and survival impact in rats. This distinction highlights that our research provides a broader analysis of vinpocetine’s multifaceted preventive actions, whereas zolmitriptan’s study centers on apoptosis as a curative measure.

Furthermore, our study unveiled that vinpocetine’s effects extend to encompassing *STAT3* inhibitory role. It has been acknowledged that Macrophage-derived IL-6 activates STAT3 signaling and promotes EMT [74, 75]. STAT3, a transcription factor, holds a pivotal role in the intricate regulation of *MMP9* expression [76]. *MMP9* is a key player in various critical processes such as tissue disruption, tumor neovascularization, and subsequent metastasis. In these processes, MMPs like MMP9 act by degrading the extracellular matrix [77]. In our study, we revealed that vinpocetine exhibited inactivation of *STAT3* signaling as indicated by the low hepatic levels of p-STAT3 in DENA-exposed rats treated with the test drug. Therefore, one potential mechanism of action for vinpocetine involves the modulation of *STAT3*. Consistent with our findings, research has shown that vinpocetine inhibits the phosphorylation and nuclear translocation of STAT3 which might contribute to reducing *MMP9* expression [78, 79].

Hypoxia is a prevalent characteristic of solid tumors. This oxygen deficiency arises due to the rapid expansion of tumors, which outpaces the available oxygen, and the compromised blood flow caused by the development of abnormal blood vessels that supply the tumor [80]. Hypoxia serves as a trigger for angiogenesis during tumor growth [81]. This migration is orchestrated by many factors including *HIF-1α* and *VEGF* [82, 83]. Importantly, hypoxia’s activation of angiogenesis increases the invasiveness of tumors and elevates the risk of metastasis. In the present study, vinpocetine’s ability to decrease the levels of HIF-1α is presumably associated with the observed antiangiogenic effects. Moreover, it has been postulated that *STAT3* is a potential modulator of *HIF-1*-mediated *VEGF* expression [84]. Furthermore, it has been documented that the level of HIF-1α was up-regulated by *STAT3* activation [85]. Hence, we can postulate that the vinpocetine’s effect on *HIF-1α* and *VEGF* is *STAT3*-mediated. In this context, a combined regulatory effect of vinpocetine on both *STAT3* and *NF-κB* might contribute to observed oncostatic potential.

Our study involved exposing rats to DENA at 100 mg/kg/week for 12 weeks, a relatively short period that primarily induced liver damage, inflammation, angiogenesis, and proliferation, leading to preneoplastic changes without the development of advanced-stage solid tumors. This protocol aligns with our goal of investigating vinpocetine’s oncostatic potential during the early stages of hepatocarcinogenesis. AFP levels were significantly increased in the DENA group, indicating tumorigenesis, while vinpocetine treatment reduced AFP levels, consistent with histological improvements and reduced neoplastic changes. Histological analysis revealed that DENA induced areas of high cell proliferation, abnormal nuclei, and inflammation, which were absent in the DENA/VPCTN group. Markers of proliferation (Ki-67), angiogenesis (VEGF), and cell survival (CCND1) were elevated in the DENA group and reduced by vinpocetine treatment. Additionally, vinpocetine mitigated DENA-induced oxidative stress, restored antioxidant markers (SOD, GSH), improved liver function (ALT, AST, γGT), reduced inflammatory markers (NF-κB p65, IL-6, MCP-1, ICAM-1), and inhibited angiogenesis (VEGF, HIF-1α), tissue remodeling (MMP-9), and survival pathways (*STAT3*). Vinpocetine also promoted apoptosis by increasing pro-apoptotic markers (Bax, active caspase-3) and reducing anti-apoptotic BCL-2. These findings demonstrate vinpocetine’s protective and anti-tumorigenic effects in the early stages of HCC development.

Despite the promising findings, our study has limitations that should be acknowledged. The use of an animal model means that the results may not fully translate to human physiology and pathology. While we identified several molecular targets and pathways affected by vinpocetine, the precise mechanisms of action remain to be fully elucidated. The study focused primarily on the preventive effects of vinpocetine on the initiation and progression of HCC; its curative effects should be evaluated in a separate study. Additionally, we employed a relatively short administration period of DENA, resulting in observations limited to the early stages of hepatocarcinogenesis. The effects of vinpocetine should be evaluated in subsequent, more advanced stages of hepatocarcinogenesis in future studies. Furthermore, potential interactions of vinpocetine with other therapeutic agents were not explored, which could be crucial for its application in combination therapies.

## Conclusion

Vinpocetine increased the survival rate of DENA-intoxicated rats and improved the ultrastructure of their livers. Additionally, vinpocetine reduced the liver weight index, mitigated liver oxidative stress, and improved liver function. In both in vitro and in vivo settings, vinpocetine demonstrated oncostatic potential. Vinpocetine also successfully deactivated *NF-κB* and *STAT3* and downregulated HIF-1α, along with their associated transcription proteins. Our findings strongly suggest that vinpocetine holds promise as an option for the prevention of hepatocarcinogenesis by targeting a range of oncogenic proteins simultaneously. Nonetheless, the combined targeting of *NF-κB* and *STAT3* offers a promising approach for the management of liver cancer such as HCC, necessitating further compelling experiments to confirm the robustness of vinpocetine’s effects. Future studies are needed to validate and establish causal links between our observed effects, allowing for a more in-depth exploration of the mechanisms underlying vinpocetine’s effects and identifying pivotal determinants of outcomes.

## Supporting information

S1 File (PDF)

## Abbreviations

AFP: alpha fetoprotein
AKT: protein kinase B
ALT: alanine transaminase
AST: aspartate aminotransferase
Bak: Bcl-2 homologous antagonist/killer
Bax: BCL-2-associated X protein
BCL-2: B-cell lymphoma 2
Bcl-xL: B-cell lymphoma-extra-large
DAB: 3,30-diaminobenzidine
DENA: diethylnitrosamine
EMT: epithelial-mesenchymal transition
GSH: reduced glutathione
GSK-3β: glycogen synthase kinase 3 beta
HCC: hepatocellular carcinoma
HPF: high-power fields
ICAM-1: intercellular adhesion molecule-1
IKKβ: IκB kinase β
IκB: inhibitor of nuclear factor kappa B
LDH: lactate dehydrogenase
MCP-1: monocyte chemoattractant protein-1
MDA: malondialdehyde
MTT: 3-(4,5-Dimethylthiazol-2-yl)-2,5-diphenyltetrazolium bromide
NF-κB: nuclear transcription factor kappa B
NO: nitric oxide
PI3K: phosphoinositide 3-kinase
PKM2: pyruvate kinase M2
ROS: reactive oxygen species
SOD: superoxide dismutase
STAT3: signal transducer and activator of transcription 3
TBS: Tris-buffered saline
TGF-β: transforming growth factor-beta
TNF-α: tumor necrosis factor-alpha
VEGF: vascular endothelial growth factor
γGT: gamma-glutamyl transferase
