# Supplementary material for: Unlocking vinpocetine’s oncostatic potential in early-stage hepatocellular carcinoma: A new approach to oncogenic modulation by a nootropic drug
Source: PLoS One. 2024 Oct 31;19(10):e0312572. doi: 10.1371/journal.pone.0312572 (PMC11527275; doi:10.1371/journal.pone.0312572)
Supplement: S1 File — (PDF) [file pone.0312572.s001.pdf]

Table format: XY		X	Group A		
		Conc.	% growth inh.		
		X	Mean	SEM	N
1	Title	0.000	0.000000	2.877116	3
2	Title	5.000	9.480000	3.752300	3
3	Title	10.000	17.150000	2.887710	3
4	Title	20.000	39.880000	2.116000	3
5	Title	40.000	64.400000	2.309000	3
6	Title	80.000	84.980000	1.550000	3
7	Title				
8	Title				
9	Title				
10	Title		50.000000		

Table format: **XY**		X	Group A		
		Conc.	% growth inh.		
		X	Mean	SEM	N
1	Title	0.0001	100.000000	2.877116	3
2	Title	5.0000	90.527600	3.752300	3
3	Title	10.0000	82.850000	2.887710	3
4	Title	20.0000	60.120000	2.116000	3
5	Title	40.0000	35.400000	2.309000	3
6	Title	80.0000	15.020000	1.550000	3

Table format: **XY**		X	Group A		
		Conc.	% growth inh.		
		X	Mean	SEM	N
1	Title	0.0001	0.000000	2.877116	3
2	Title	5.0000	9.480000	3.752300	3
3	Title	10.0000	17.150000	2.887710	3
4	Title	20.0000	39.880000	2.116000	3
5	Title	40.0000	64.400000	2.309000	3
6	Title	80.0000	84.980000	1.550000	3

Table format: **Grouped**		Group A			Group B			Group C	
		0			5			10	
		Mean	SD	N	Mean	SD	N	Mean	SD
1	Title	100.000000	2.877116	3	90.527600	3.752300	3	82.850	2.888

		Group D			Group E			Group F	
		20			40			80	
	N	Mean	SD	N	Mean	SD	N	Mean	SD
1	3	60.120	2.116	3	35.400	2.309	3	15.02	1.55

	N
1	3

Table format: **Grouped**		Group A			Group B			Group C	
		0			5			10	
		Mean	SD	N	Mean	SD	N	Mean	SD
1	Title	100.00000	6.25600	3	86.1400	3.0500	3	74.61233	2.75200

		Group D			Group E			Group F	
		20			40			80	
	N	Mean	SD	N	Mean	SD	N	Mean	SD
1	3	52.199	2.210	3	28.488	2.151	3	12.63	1.11

	N
1	3

Table format: **Grouped**		Group A			Group B			Group C	
		0			5			10	
		Mean	SD	N	Mean	SD	N	Mean	SD
1	Title	100.000	4.096	3	72.140	2.450	3	63.32400	2.45200

		Group D			Group E			Group F	
		20			40			80	
	N	Mean	SD	N	Mean	SD	N	Mean	SD
1	3	43.1990000	2.1100000	3	22.0540000	2.0507000	3	7.6300	0.8090

	N
1	3

Table format: **Grouped**		Group A			Group B			Group C		
		24 h			48 h			72 h		
		Mean	SD	N	Mean	SD	N	Mean	SD	N
1	0	100.0000(	2.877116	3	100.0000(	6.256000(	3	100.0000(	4.095600(	3
2	5	90.52760(	3.752300(	3	86.14000(	3.050000(	3	72.14000(	2.450000(	3
3	10	82.85000(	2.887710(	3	74.61233	2.752000(	3	63.32400(	2.452000(	3
4	20	60.12000(	2.116000(	3	52.19900(	2.210000(	3	43.19900(	2.110000(	3
5	40	35.40000(	2.309000(	3	28.48800(	2.150700(	3	22.05400(	2.050700(	3
6	80	15.02000(	1.550000(	3	12.63000(	1.109000(	3	7.630000(	0.809000(	3

	Group A	Group B	Group C	Group D	Group E	Group F
	0	5	10	20	40	80
1	15.60000	15.70000	15.85500	17.3455000	21.5878750	20.6024000
2	17.52000	14.13333	13.16745	16.3054200	18.5573254	18.6005745
3	14.25333	18.45863	18.62578	19.6057520	15.8575745	22.5065450

	Group A	Group B	Group C
	0	10	20
1	3.28000	2.87600	2.45100
2	3.46000	2.98400	2.70640
3	3.57200	3.15400	2.39820

	Group A	Group B	Group C
	0	10	20
1	3.29000	4.369750	5.621000
2	3.80000	4.889500	5.497800
3	3.60000	4.391310	6.056050

	Group A	Group B	Group C
	0	10	20
1	0.863293	1.206310	1.357456
2	1.026417	1.072170	1.252881
3	1.110290	0.952360	1.129546

	Group A	Group B	Group C	Group D
	Normal	VPCTN	DENA	ENA/VPCT
1	3.841650	4.262850	6.95465	6.160850
2	4.156200	4.144050	6.80200	6.213600
3	4.187700	4.402800	5.03725	5.213600
4	4.523200	5.318450	7.63730	5.152950
5	4.660200	4.625700	5.85195	4.811850
6	5.267750	5.108950	7.83250	4.607450

	Group A	Group B	Group C	Group D
	Normal	VPCTN	DENA	DENA/VPCTN
1	298.4520	279.97970	291.65830	290.1478
2	317.1058	305.69220	257.53040	275.8019
3	326.4316	349.02230	278.60540	305.7927
4	335.7585	303.78700	246.04090	260.1278
5	335.7585	328.54690	261.78620	323.3381
6	354.4123	358.54500	288.22280	280.7824

	Group A	Group B	Group C	Group D
	Normal	VPCTN	DENA	DENA/VPCTN
1	0.012872000	0.015226	0.023845000	0.021233
2	0.013107000	0.013556	0.026412000	0.022529
3	0.012829000	0.012615	0.018080000	0.017049
4	0.013472000	0.017507	0.031041000	0.019809
5	0.013880000	0.014079	0.022354000	0.014882
6	0.014863000	0.014249	0.027175000	0.016409

	Group A	Group B	Group C	Group D
	Normal	VPCTN	DENA	DENA/VPCTN
1	1.438965	1.506033	4.491450	4.020950
2	1.414740	1.050397	4.201807	2.504411
3	1.059440	0.999744	5.712776	3.449186
4	1.191870	1.404742	5.882844	2.760366
5	1.721590	1.588832	6.616334	4.216460
6	1.787846	1.614662	3.595782	2.675168

	Group A	Group B
	DENA	DENA/VPCTN
1	9.00000	2.00000
2	10.00000	2.00000
3	10.00000	3.00000
4	9.00000	3.00000
5	10.00000	3.00000
6	9.00000	5.00000

	Group A	Group B	Group C	Group D
	Normal	VPCTN	DENA	DENA/VPCTN
1	27.75956000	26.69070000	277.1136000	111.03820000
2	36.73181000	28.03822000	213.7428000	66.33522000
3	26.28109000	31.52924000	301.8074000	105.12440000
4	26.70141000	32.76917000	241.8985000	121.89370000
5	23.99338000	33.98045000	193.3003000	95.97348000
6	32.45104000	24.89060000	241.0038000	129.80410000

	Group A	Group B	Group C	Group D
	Normal	VPCTN	DENA	DENA/VPCTN
1	62.23407000	56.95637000	484.0191000	143.1384000
2	42.23536000	40.25515000	348.8821000	112.5881000
3	68.46501000	59.90681000	380.6110000	157.4695000
4	59.97628000	61.75371000	411.4675000	168.1214000
5	60.85988000	49.30878000	363.9391000	139.9777000
6	59.25306000	69.54344000	356.9584000	136.2821000

	Group A	Group B	Group C	Group D
	Normal	VPCTN	DENA	DENA/VPCTN
1	9.45988200	10.45264000	76.6250400	51.48925000
2	6.21933300	7.75416500	50.3766100	40.42566000
3	8.85259700	8.60041800	64.1620300	57.54188000
4	11.04429000	15.28508000	89.4587100	41.78789000
5	7.36607500	8.66836000	71.8864700	37.68668000
6	9.42132400	10.33505000	76.3127700	68.78263000

	Group A	Group B	Group C	Group D
	Normal	VPCTN	DENA	DENA/VPCTN
1	21.86514	23.81794	74.21531	57.13030
2	23.43154	21.63258	137.59710	39.86310
3	15.17000	12.13950	89.19960	45.14000
4	27.79964	17.52951	96.73606	37.59930
5	27.88492	23.55664	112.36970	42.00984
6	18.44590	21.30197	119.15610	58.89180

	Group A	Group B	Group C	Group D
	Normal	VPCTN	DENA	DENA/VPCTN
1	19.2088	18.8741	7.3577	9.8163
2	17.1938	18.7318	7.9982	12.0431
3	19.3688	17.4774	8.2035	13.5219
4	18.7078	20.4841	5.6892	10.7618
5	17.8699	19.3427	8.2875	14.9305
6	18.8156	20.2271	8.6959	12.7605

	Group A	Group B	Group C	Group D
	Normal	VPCTN	DENA	DENA/VPCTN
1	10.30590	12.424100	3.1776	8.878305
2	10.58310	10.676560	4.5696	5.788956
3	12.55320	12.662890	2.3808	7.528286
4	14.25600	14.410440	3.7728	7.571637
5	9.10602	9.186804	1.3920	6.814473
6	11.33748	11.411930	3.1776	8.436965

	Group A	Group B	Group C	Group D
	Normal	VPCTN	DENA	DENA/VPCTN
1	11.2049	12.5565	20.6267	15.3637
2	11.9785	10.5261	30.0904	18.1171
3	11.2472	13.6782	27.8134	20.1048
4	12.3210	11.6544	29.4112	18.7893
5	12.5341	12.3287	26.2895	15.9812
6	12.5271	9.7213	24.1376	18.7356

	Group A	Group B	Group C	Group D
	Normal	VPCTN	DENA	ENA/VPCT
1	6.0120	6.006100	25.37185	10.3280
2	5.8365	4.993450	25.01969	16.8550
3	5.1480	4.512650	25.75754	9.4460
4	5.8365	5.283650	22.95708	11.5260
5	5.4810	4.872250	21.46462	12.6240
6	5.3820	4.558500	18.16108	8.9160

	Group A	Group B	Group C	Group D
	Normal	VPCTN	DENA	DENA/VPCTN
1	2.3853	2.2356	4.7119	4.3952
2	1.8706	2.0758	4.8157	3.4967
3	2.1162	1.9847	3.9466	3.1698
4	1.8902	2.3601	4.9498	3.0401
5	2.3209	2.1006	4.9205	5.3965
6	2.2317	2.1554	6.0222	3.0979

	Group A	Group B	Group C	Group D
	Normal	VPCTN	DENA	DENA/VPCTN
1	4.1225	4.6841	27.1285	10.7853
2	4.6471	5.8058	38.8012	15.0749
3	5.7246	5.0256	38.5634	21.0107
4	5.1597	5.1764	33.7148	17.8632
5	5.4853	5.0652	37.9276	12.9701
6	4.8959	4.8089	32.1257	13.4010

	Group A	Group B	Group C	Group D	Group E
	Normal	VPCTN	DENA	DENA/VPCTN	Data Set-E
1	2.457378	2.334509	12.286890	5.651969	
2	1.504165	1.465457	8.250825	3.065380	
3	2.375868	2.257075	11.879340	5.464496	
4	1.927574	1.831195	8.817870	4.433420	
5	2.190056	2.080553	10.950280	5.037129	
6	2.023186	1.922027	10.115930	4.653328	

Table format: **Grouped**		Group A			Group B			Group C			Group D		
		Normal			VPCTN			DENA			DENA/VPCTN		
		Mean	SD	N	Mean	SD	N	Mean	SD	N	Mean	SD	N
1	Title	1.00	0.17	6	0.93888	0.15320	6	5.893000	0.951022	6	2.690000	0.686900	6

	Group A	Group B	Group C	Group D
	Normal	VPCTN	DENA	ENA/VPCT
1	75.84516	79.85142	346.1101	216.3691
2	74.10632	74.08478	290.2554	193.5042
3	79.98075	76.72412	199.2191	143.2599
4	99.17613	88.99132	318.9525	98.78442
5	75.65238	76.72412	302.5692	96.95680
6	64.17279	71.67292	295.8299	103.6480

	Group A	Group B	Group C	Group D	Group E
	Normal	VPCTN	DENA	DENA/VPCTN	Data Set-E
1	12.80000	14.90000	33.70000	22.90000	
2	17.90000	14.60000	40.90000	24.70000	
3	11.80000	10.70000	28.70000	27.80000	
4	13.70000	9.70000	25.70000	23.40000	
5	10.70000	11.60000	37.90000	18.70000	
6	9.80000	8.30000	20.60000	21.70000	

	Group A	Group B	Group C	Group D
	Normal	VPCTN	DENA	DENA/VPCTN
1	45.1225	30.6841	786.1285	180.7853
2	33.6471	29.3058	551.8012	135.0749
3	32.7246	35.0256	854.5634	166.0107
4	34.1597	31.1764	657.7148	141.8632
5	32.4853	29.4652	724.9276	151.9701
6	36.8959	33.8089	682.1257	168.4010

	Group A	Group B	Group C	Group D	Group E
	Normal	VPCTN	DENA	DENA/VPCTN	Data Set-E
1	73.19565	69.53587	314.74130	168.35000	
2	60.57029	57.54178	260.45220	139.31170	
3	76.01154	72.21096	244.84960	174.82650	
4	55.44430	68.25209	308.93050	165.24190	
5	74.96522	54.81696	322.35040	188.82000	
6	75.65074	71.86820	325.29820	173.99670	

	Group A	Group B	Group C	Group D
	Normal	VPCTN	DENA	DENA/VPCTN
1	97.72650	91.24002	520.91530	180.6504
2	97.28681	90.49445	491.40450	146.4774
3	118.61540	115.79740	573.18540	201.9258
4	128.13320	121.57730	509.24790	218.0921
5	114.64580	106.80830	500.46650	224.0371
6	121.98650	112.38630	533.89930	172.3766

	Group A	Group B	Group C	Group D
	Normal	VPCTN	DENA	DENA/VPCTN
1	2.30	2.10	12.900	5.90
2	2.50	2.40	10.800	4.90
3	1.40	1.20	8.100	3.80
4	1.80	1.09	7.000	5.10
5	2.90	2.80	9.800	6.70
6	2.20	2.40	12.500	4.70

	Group A	Group B	Group C	Group D
	Normal	VPCTN	DENA	DENA/VPCTN
1	2.471521	1.596016	4.891613	3.441738
2	1.567287	1.798866	5.454022	3.020702
3	1.422678	0.926028	3.380615	4.692619
4	2.394566	1.806214	5.226680	2.705421
5	1.931264	1.753160	4.368960	2.537490
6	1.684280	2.036060	4.477610	2.829574

	Group A	Group B	Group C	Group D
	Normal	VPCTN	DENA	DENA/VPCTN
1	1.204146	1.1501190	0.410486	0.6781260
2	1.064267	1.0950310	0.452088	0.7000170
3	1.107738	1.1242210	0.314782	0.7557920
4	0.701510	0.7743340	0.388966	0.9647460
5	1.037710	1.0352500	0.539560	0.6275130
6	0.972110	0.9555050	0.595812	0.5620690

	Group A	Group B	Group C	Group D
	Normal	VPCTN	DENA	DENA/VPCTN
1	0.093150	0.0870940	0.0341820	0.0771320
2	0.075920	0.0917040	0.0468350	0.0595810
3	0.102500	0.1160630	0.0275930	0.0762600
4	0.106633	0.0747192	0.0635500	0.0718150
5	0.115210	0.1044440	0.0385400	0.0855830
6	0.123533	0.0942024	0.0476170	0.0788010

Table format: **Grouped**		Group A<br>Normal			Group B<br>VPCTN			Group C<br>DENA			Group D<br>DENA/VPCT	
		Mean	SD	N	Mean	SD	N	Mean	SD	N	Mean	SD
1	Title	1	1e-001	6	0.903	0.170	6	6.17	0.94	6	3.81373	0.58530

	N
	N
1	6

	Group A	Group B	Group C	Group D
	Normal	VPCTN	DENA	DENA/VPCTN
1	0.889223	0.989563	2.369613	1.420458
2	0.853964	0.829463	2.071430	0.711079
3	0.990771	0.949739	2.361338	1.586860
4	0.964251	0.845158	2.618038	1.481778
5	1.154399	1.034806	1.287002	1.896476
6	1.147393	0.832148	3.054378	1.771379

Table format: **Grouped**		Group A Normal			Group B VPCTN			Group C DENA			Group D DENA/VPCTN		
		Mean	SD	N	Mean	SD	N	Mean	SD	N	Mean	SD	N
1	Title	1.00	0.15	6	0.91100C	0.11500C	6	5.12000C	0.93580C	6	3.21650C	0.51000C	6

Table format: **Survival**		X	Group A	Group B
		Days	DENA	DENA/VPCTN
		X	Y	Y
1	Title	40	1	
2	Title	57	1	
3	Title	66	1	
4	Title	74	1	
5	Title	78	1	
6	Title	82	1	
7	Title	84	0	
8	Title	84	0	
9	Title	84	0	
10	Title	84	0	
11	Title	84	0	
12	Title	84	0	
13	Title	68		1
14	Title	84		0
15	Title	84		0
16	Title	84		0
17	Title	84		0
18	Title	84		0
19	Title	84		0
20	Title	84		0
21	Title	84		0
22	Title	84		0

	Group A	Group B	Group C	Group D
	Normal	VPCTN	DENA	DENA/VPCTN
1	4.30	5.10	2.400	5.90
2	3.50	4.40	2.800	7.90
3	4.60	3.20	4.100	8.80
4	3.80	5.30	2.300	9.10
5	4.90	3.80	1.800	10.70
6	3.50	4.40	2.500	9.70

	Group A	Group B	Group C	Group D
	Normal	VPCTN	DENA	DENA/VPCTN
1	3.150000	7.0940000	8.2000000	5.2713200
2	5.920000	4.2000000	8.3500000	5.9581000
3	2.500000	3.3000000	9.3000000	6.2600000
4	6.633000	4.7170000	6.3550000	7.1500000
5	5.210000	4.4200000	8.5400000	5.5830000
6	3.533000	4.2024000	7.6170000	4.8000000

Ordinary one-way ANOVA
ANOVA results

1	Table Analyzed	BCL-2			
2	Data sets analyzed	A-D			
3					
4	**ANOVA summary**				
5	F	29.63			
6	P value	<0.0001			
7	P value summary	****			
8	Significant diff. among means (P < 0.05)?	Yes			
9	R squared	0.8164			
10					
11	**Brown-Forsythe test**				
12	F (DFn, DFd)	0.7419 (3, 20)			
13	P value	0.5396			
14	P value summary	ns			
15	Are SDs significantly different (P < 0.05)?	No			
16					
17	**Bartlett's test**				
18	Bartlett's statistic (corrected)	3.491			
19	P value	0.3219			
20	P value summary	ns			
21	Are SDs significantly different (P < 0.05)?	No			
22					
23	**ANOVA table**	**SS**	**DF**	**MS**	**F (DFn, DFd)**
24	Treatment (between columns)	33.71	3	11.24	F (3, 20) = 29.63
25	Residual (within columns)	7.585	20	0.3792	
26	Total	41.30	23		
27					
28	**Data summary**				
29	Number of treatments (columns)	4			
30	Number of values (total)	24			

1	
2	
3	
4	
5	
6	
7	
8	
9	
10	
11	
12	
13	
14	
15	
16	
17	
18	
19	
20	
21	
22	
23	**P value**
24	P<0.0001
25	
26	
27	
28	
29	
30	

Ordinary one-way ANOVA<br>Multiple comparisons					
1	Number of families	1			
2	Number of comparisons per family	6			
3	Alpha	0.05			
4					
5	**Tukey's multiple comparisons test**	**Mean Diff.**	**95.00% CI of diff.**	**Below threshold?**	**Summary**
6	Normal vs. VPCTN	0.2592	-0.7359 to 1.254	No	ns
7	Normal vs. DENA	-2.721	-3.716 to -1.726	Yes	****
8	Normal vs. DENA/VPCTN	-1.293	-2.288 to -0.2975	Yes	**
9	VPCTN vs. DENA	-2.981	-3.976 to -1.985	Yes	****
10	VPCTN vs. DENA/VPCTN	-1.552	-2.547 to -0.5567	Yes	**
11	DENA vs. DENA/VPCTN	1.429	0.4335 to 2.424	Yes	**
12					
13	**Test details**	**Mean 1**	**Mean 2**	**Mean Diff.**	**SE of diff.**
14	Normal vs. VPCTN	1.912	1.653	0.2592	0.3555
15	Normal vs. DENA	1.912	4.633	-2.721	0.3555
16	Normal vs. DENA/VPCTN	1.912	3.205	-1.293	0.3555
17	VPCTN vs. DENA	1.653	4.633	-2.981	0.3555
18	VPCTN vs. DENA/VPCTN	1.653	3.205	-1.552	0.3555
19	DENA vs. DENA/VPCTN	4.633	3.205	1.429	0.3555

1				
2				
3				
4				
5	**Adjusted P Value**			
6	0.8843	A-B		
7	<0.0001	A-C		
8	0.0082	A-D		
9	<0.0001	B-C		
10	0.0016	B-D		
11	0.0035	C-D		
12				
13	**n1**	**n2**	**q**	**DF**
14	6	6	1.031	20
15	6	6	10.82	20
16	6	6	5.142	20
17	6	6	11.86	20
18	6	6	6.173	20
19	6	6	5.683	20

Ordinary one-way ANOVA<br>ANOVA results					
1	Table Analyzed	MMP-9			
2	Data sets analyzed	A-D			
3					
4	**ANOVA summary**				
5	F	176.9			
6	P value	<0.0001			
7	P value summary	****			
8	Significant diff. among means (P < 0.05)?	Yes			
9	R squared	0.9637			
10					
11	**Brown-Forsythe test**				
12	F (DFn, DFd)	1.926 (3, 20)			
13	P value	0.1579			
14	P value summary	ns			
15	Are SDs significantly different (P < 0.05)?	No			
16					
17	**Bartlett's test**				
18	Bartlett's statistic (corrected)	13.14			
19	P value	0.0043			
20	P value summary	**			
21	Are SDs significantly different (P < 0.05)?	Yes			
22					
23	**ANOVA table**	**SS**	**DF**	**MS**	**F (DFn, DFd)**
24	Treatment (between columns)	211818	3	70606	F (3, 20) = 176.9
25	Residual (within columns)	7984	20	399.2	
26	Total	219802	23		
27					
28	**Data summary**				
29	Number of treatments (columns)	4			
30	Number of values (total)	24			

1	
2	
3	
4	
5	
6	
7	
8	
9	
10	
11	
12	
13	
14	
15	
16	
17	
18	
19	
20	
21	
22	
23	**P value**
24	P<0.0001
25	
26	
27	
28	
29	
30	

Ordinary one-way ANOVA<br>Multiple comparisons					
1	Number of families	1			
2	Number of comparisons per family	6			
3	Alpha	0.05			
4					
5	**Tukey's multiple comparisons test**	**Mean Diff.**	**95.00% CI of diff.**	**Below threshold?**	**Summary**
6	Normal vs. VPCTN	3.602	-28.69 to 35.89	No	ns
7	Normal vs. DENA	-226.8	-259.1 to -194.5	Yes	****
8	Normal vs. DENA/VPCTN	-99.12	-131.4 to -66.83	Yes	****
9	VPCTN vs. DENA	-230.4	-262.7 to -198.1	Yes	****
10	VPCTN vs. DENA/VPCTN	-102.7	-135.0 to -70.43	Yes	****
11	DENA vs. DENA/VPCTN	127.7	95.39 to 160.0	Yes	****
12					
13	**Test details**	**Mean 1**	**Mean 2**	**Mean Diff.**	**SE of diff.**
14	Normal vs. VPCTN	69.31	65.70	3.602	11.54
15	Normal vs. DENA	69.31	296.1	-226.8	11.54
16	Normal vs. DENA/VPCTN	69.31	168.4	-99.12	11.54
17	VPCTN vs. DENA	65.70	296.1	-230.4	11.54
18	VPCTN vs. DENA/VPCTN	65.70	168.4	-102.7	11.54
19	DENA vs. DENA/VPCTN	296.1	168.4	127.7	11.54

1				
2				
3				
4				
5	**Adjusted P Value**			
6	0.9891	A-B		
7	<0.0001	A-C		
8	<0.0001	A-D		
9	<0.0001	B-C		
10	<0.0001	B-D		
11	<0.0001	C-D		
12				
13	**n1**	**n2**	**q**	**DF**
14	6	6	0.4416	20
15	6	6	27.80	20
16	6	6	12.15	20
17	6	6	28.25	20
18	6	6	12.59	20
19	6	6	15.65	20

**Ordinary one-way ANOVA**<br>ANOVA results					
1	Table Analyzed	TGF-b			
2	Data sets analyzed	A-D			
3					
4	**ANOVA summary**				
5	F	99.50			
6	P value	<0.0001			
7	P value summary	****			
8	Significant diff. among means (P < 0.05)?	Yes			
9	R squared	0.9372			
10					
11	**Brown-Forsythe test**				
12	F (DFn, DFd)	6.099 (3, 20)			
13	P value	0.0040			
14	P value summary	**			
15	Are SDs significantly different (P < 0.05)?	Yes			
16					
17	**Bartlett's test**				
18	Bartlett's statistic (corrected)	15.30			
19	P value	0.0016			
20	P value summary	**			
21	Are SDs significantly different (P < 0.05)?	Yes			
22					
23	**ANOVA table**	**SS**	**DF**	**MS**	**F (DFn, DFd)**
24	Treatment (between columns)	279.1	3	93.05	F (3, 20) = 99.50
25	Residual (within columns)	18.70	20	0.9352	
26	Total	297.8	23		
27					
28	**Data summary**				
29	Number of treatments (columns)	4			
30	Number of values (total)	24			

1	
2	
3	
4	
5	
6	
7	
8	
9	
10	
11	
12	
13	
14	
15	
16	
17	
18	
19	
20	
21	
22	
23	**P value**
24	P<0.0001
25	
26	
27	
28	
29	
30	

Ordinary one-way ANOVA<br>Multiple comparisons					
1	Number of families	1			
2	Number of comparisons per family	6			
3	Alpha	0.05			
4					
5	**Tukey's multiple comparisons test**	**Mean Diff.**	**95.00% CI of diff.**	**Below threshold?**	**Summary**
6	Normal vs. VPCTN	0.09790	-1.465 to 1.661	No	ns
7	Normal vs. DENA	-8.304	-9.867 to -6.741	Yes	****
8	Normal vs. DENA/VPCTN	-2.638	-4.201 to -1.075	Yes	***
9	VPCTN vs. DENA	-8.402	-9.964 to -6.839	Yes	****
10	VPCTN vs. DENA/VPCTN	-2.736	-4.299 to -1.173	Yes	***
11	DENA vs. DENA/VPCTN	5.666	4.103 to 7.229	Yes	****
12					
13	**Test details**	**Mean 1**	**Mean 2**	**Mean Diff.**	**SE of diff.**
14	Normal vs. VPCTN	2.080	1.982	0.09790	0.5583
15	Normal vs. DENA	2.080	10.38	-8.304	0.5583
16	Normal vs. DENA/VPCTN	2.080	4.718	-2.638	0.5583
17	VPCTN vs. DENA	1.982	10.38	-8.402	0.5583
18	VPCTN vs. DENA/VPCTN	1.982	4.718	-2.736	0.5583
19	DENA vs. DENA/VPCTN	10.38	4.718	5.666	0.5583

1				
2				
3				
4				
5	**Adjusted P Value**			
6	0.9980	A-B		
7	<0.0001	A-C		
8	0.0007	A-D		
9	<0.0001	B-C		
10	0.0005	B-D		
11	<0.0001	C-D		
12				
13	**n1**	**n2**	**q**	**DF**
14	6	6	0.2480	20
15	6	6	21.03	20
16	6	6	6.682	20
17	6	6	21.28	20
18	6	6	6.930	20
19	6	6	14.35	20

Ordinary one-way ANOVA<br>ANOVA results					
1	Table Analyzed	VEGF IHC			
2	Data sets analyzed	A-D			
3					
4	**ANOVA summary**				
5	F	24.83			
6	P value	<0.0001			
7	P value summary	****			
8	Significant diff. among means (P < 0.05)?	Yes			
9	R squared	0.7883			
10					
11	**Brown-Forsythe test**				
12	F (DFn, DFd)	4.853 (3, 20)			
13	P value	0.0107			
14	P value summary	*			
15	Are SDs significantly different (P < 0.05)?	Yes			
16					
17	**Bartlett's test**				
18	Bartlett's statistic (corrected)	8.259			
19	P value	0.0410			
20	P value summary	*			
21	Are SDs significantly different (P < 0.05)?	Yes			
22					
23	**ANOVA table**	**SS**	**DF**	**MS**	**F (DFn, DFd)**
24	Treatment (between columns)	1551	3	517.1	F (3, 20) = 24.83
25	Residual (within columns)	416.5	20	20.83	
26	Total	1968	23		
27					
28	**Data summary**				
29	Number of treatments (columns)	4			
30	Number of values (total)	24			

1	
2	
3	
4	
5	
6	
7	
8	
9	
10	
11	
12	
13	
14	
15	
16	
17	
18	
19	
20	
21	
22	
23	**P value**
24	P<0.0001
25	
26	
27	
28	
29	
30	

**Ordinary one-way ANOVA**<br>Multiple comparisons					
1	Number of families	1			
2	Number of comparisons per family	6			
3	Alpha	0.05			
4					
5	**Tukey's multiple comparisons test**	**Mean Diff.**	**95.00% CI of diff.**	**Below threshold?**	**Summary**
6	Normal vs. VPCTN	1.150	-6.225 to 8.525	No	ns
7	Normal vs. DENA	-18.47	-25.84 to -11.09	Yes	****
8	Normal vs. DENA/VPCTN	-10.42	-17.79 to -3.042	Yes	**
9	VPCTN vs. DENA	-19.62	-26.99 to -12.24	Yes	****
10	VPCTN vs. DENA/VPCTN	-11.57	-18.94 to -4.192	Yes	**
11	DENA vs. DENA/VPCTN	8.050	0.6753 to 15.42	Yes	*
12					
13	**Test details**	**Mean 1**	**Mean 2**	**Mean Diff.**	**SE of diff.**
14	Normal vs. VPCTN	12.78	11.63	1.150	2.635
15	Normal vs. DENA	12.78	31.25	-18.47	2.635
16	Normal vs. DENA/VPCTN	12.78	23.20	-10.42	2.635
17	VPCTN vs. DENA	11.63	31.25	-19.62	2.635
18	VPCTN vs. DENA/VPCTN	11.63	23.20	-11.57	2.635
19	DENA vs. DENA/VPCTN	31.25	23.20	8.050	2.635

1				
2				
3				
4				
5	**Adjusted P Value**			
6	0.9714	A-B		
7	<0.0001	A-C		
8	0.0040	A-D		
9	<0.0001	B-C		
10	0.0015	B-D		
11	0.0293	C-D		
12				
13	**n1**	**n2**	**q**	**DF**
14	6	6	0.6173	20
15	6	6	9.912	20
16	6	6	5.591	20
17	6	6	10.53	20
18	6	6	6.208	20
19	6	6	4.321	20

Ordinary one-way ANOVA<br>ANOVA results					
1	Table Analyzed	CCND1 liver			
2	Data sets analyzed	A-D			
3					
4	**ANOVA summary**				
5	F	445.6			
6	P value	<0.0001			
7	P value summary	****			
8	Significant diff. among means (P < 0.05)?	Yes			
9	R squared	0.9853			
10					
11	**Brown-Forsythe test**				
12	F (DFn, DFd)	1.927 (3, 20)			
13	P value	0.1577			
14	P value summary	ns			
15	Are SDs significantly different (P < 0.05)?	No			
16					
17	**Bartlett's test**				
18	Bartlett's statistic (corrected)	5.715			
19	P value	0.1263			
20	P value summary	ns			
21	Are SDs significantly different (P < 0.05)?	No			
22					
23	**ANOVA table**	**SS**	**DF**	**MS**	**F (DFn, DFd)**
24	Treatment (between columns)	692747	3	230916	F (3, 20) = 445.6
25	Residual (within columns)	10365	20	518.3	
26	Total	703112	23		
27					
28	**Data summary**				
29	Number of treatments (columns)	4			
30	Number of values (total)	24			

1	
2	
3	
4	
5	
6	
7	
8	
9	
10	
11	
12	
13	
14	
15	
16	
17	
18	
19	
20	
21	
22	
23	**P value**
24	P<0.0001
25	
26	
27	
28	
29	
30	

**Ordinary one-way ANOVA**<br>Multiple comparisons					
1	Number of families	1			
2	Number of comparisons per family	6			
3	Alpha	0.05			
4					
5	**Tukey's multiple comparisons test**	**Mean Diff.**	**95.00% CI of diff.**	**Below threshold?**	**Summary**
6	Normal vs. VPCTN	6.682	-30.11 to 43.47	No	ns
7	Normal vs. DENA	-408.5	-445.2 to -371.7	Yes	****
8	Normal vs. DENA/VPCTN	-77.53	-114.3 to -40.74	Yes	****
9	VPCTN vs. DENA	-415.1	-451.9 to -378.3	Yes	****
10	VPCTN vs. DENA/VPCTN	-84.21	-121.0 to -47.42	Yes	****
11	DENA vs. DENA/VPCTN	330.9	294.1 to 367.7	Yes	****
12					
13	**Test details**	**Mean 1**	**Mean 2**	**Mean Diff.**	**SE of diff.**
14	Normal vs. VPCTN	113.1	106.4	6.682	13.14
15	Normal vs. DENA	113.1	521.5	-408.5	13.14
16	Normal vs. DENA/VPCTN	113.1	190.6	-77.53	13.14
17	VPCTN vs. DENA	106.4	521.5	-415.1	13.14
18	VPCTN vs. DENA/VPCTN	106.4	190.6	-84.21	13.14
19	DENA vs. DENA/VPCTN	521.5	190.6	330.9	13.14

1				
2				
3				
4				
5	**Adjusted P Value**			
6	0.9561	A-B		
7	<0.0001	A-C		
8	<0.0001	A-D		
9	<0.0001	B-C		
10	<0.0001	B-D		
11	<0.0001	C-D		
12				
13	**n1**	**n2**	**q**	**DF**
14	6	6	0.7189	20
15	6	6	43.95	20
16	6	6	8.342	20
17	6	6	44.67	20
18	6	6	9.061	20
19	6	6	35.61	20

Ordinary one-way ANOVA
ANOVA results

1	Table Analyzed	Ki-67 IHC			
2	Data sets analyzed	A-D			
3					
4	**ANOVA summary**				
5	F	47.83			
6	P value	<0.0001			
7	P value summary	****			
8	Significant diff. among means (P < 0.05)?	Yes			
9	R squared	0.8777			
10					
11	**Brown-Forsythe test**				
12	F (DFn, DFd)	5.407 (3, 20)			
13	P value	0.0069			
14	P value summary	**			
15	Are SDs significantly different (P < 0.05)?	Yes			
16					
17	**Bartlett's test**				
18	Bartlett's statistic (corrected)	12.44			
19	P value	0.0060			
20	P value summary	**			
21	Are SDs significantly different (P < 0.05)?	Yes			
22					
23	**ANOVA table**	**SS**	**DF**	**MS**	**F (DFn, DFd)**
24	Treatment (between columns)	262.8	3	87.59	F (3, 20) = 47.83
25	Residual (within columns)	36.62	20	1.831	
26	Total	299.4	23		
27					
28	**Data summary**				
29	Number of treatments (columns)	4			
30	Number of values (total)	24			

1	
2	
3	
4	
5	
6	
7	
8	
9	
10	
11	
12	
13	
14	
15	
16	
17	
18	
19	
20	
21	
22	
23	**P value**
24	P<0.0001
25	
26	
27	
28	
29	
30	

**Ordinary one-way ANOVA**<br>Multiple comparisons					
1	Number of families	1			
2	Number of comparisons per family	6			
3	Alpha	0.05			
4					
5	**Tukey's multiple comparisons test**	**Mean Diff.**	**95.00% CI of diff.**	**Below threshold?**	**Summary**
6	Normal vs. VPCTN	0.1850	-2.002 to 2.372	No	ns
7	Normal vs. DENA	-8.000	-10.19 to -5.813	Yes	****
8	Normal vs. DENA/VPCTN	-3.000	-5.187 to -0.8133	Yes	**
9	VPCTN vs. DENA	-8.185	-10.37 to -5.998	Yes	****
10	VPCTN vs. DENA/VPCTN	-3.185	-5.372 to -0.9983	Yes	**
11	DENA vs. DENA/VPCTN	5.000	2.813 to 7.187	Yes	****
12					
13	**Test details**	**Mean 1**	**Mean 2**	**Mean Diff.**	**SE of diff.**
14	Normal vs. VPCTN	2.183	1.998	0.1850	0.7813
15	Normal vs. DENA	2.183	10.18	-8.000	0.7813
16	Normal vs. DENA/VPCTN	2.183	5.183	-3.000	0.7813
17	VPCTN vs. DENA	1.998	10.18	-8.185	0.7813
18	VPCTN vs. DENA/VPCTN	1.998	5.183	-3.185	0.7813
19	DENA vs. DENA/VPCTN	10.18	5.183	5.000	0.7813

1				
2				
3				
4				
5	**Adjusted P Value**			
6	0.9952	A-B		
7	<0.0001	A-C		
8	0.0052	A-D		
9	<0.0001	B-C		
10	0.0030	B-D		
11	<0.0001	C-D		
12				
13	**n1**	**n2**	**q**	**DF**
14	6	6	0.3349	20
15	6	6	14.48	20
16	6	6	5.430	20
17	6	6	14.82	20
18	6	6	5.765	20
19	6	6	9.051	20

Ordinary one-way ANOVA
ANOVA results

1	Table Analyzed	caspase-3 liver			
2	Data sets analyzed	A-D			
3					
4	**ANOVA summary**				
5	F	23.56			
6	P value	<0.0001			
7	P value summary	****			
8	Significant diff. among means (P < 0.05)?	Yes			
9	R squared	0.7794			
10					
11	**Brown-Forsythe test**				
12	F (DFn, DFd)	0.6700 (3, 20)			
13	P value	0.5804			
14	P value summary	ns			
15	Are SDs significantly different (P < 0.05)?	No			
16					
17	**Bartlett's test**				
18	Bartlett's statistic (corrected)	1.933			
19	P value	0.5864			
20	P value summary	ns			
21	Are SDs significantly different (P < 0.05)?	No			
22					
23	**ANOVA table**	**SS**	**DF**	**MS**	**F (DFn, DFd)**
24	Treatment (between columns)	0.01274	3	0.004247	F (3, 20) = 23.56
25	Residual (within columns)	0.003606	20	0.0001803	
26	Total	0.01635	23		
27					
28	**Data summary**				
29	Number of treatments (columns)	4			
30	Number of values (total)	24			

1	
2	
3	
4	
5	
6	
7	
8	
9	
10	
11	
12	
13	
14	
15	
16	
17	
18	
19	
20	
21	
22	
23	**P value**
24	P<0.0001
25	
26	
27	
28	
29	
30	

**Ordinary one-way ANOVA**<br>Multiple comparisons					
1	Number of families	1			
2	Number of comparisons per family	6			
3	Alpha	0.05			
4					
5	**Tukey's multiple comparisons test**	**Mean Diff.**	**95.00% CI of diff.**	**Below threshold?**	**Summary**
6	Normal vs. VPCTN	0.008120	-0.01358 to 0.02982	No	ns
7	Normal vs. DENA	0.05977	0.03807 to 0.08147	Yes	****
8	Normal vs. DENA/VPCTN	0.02796	0.006265 to 0.04966	Yes	**
9	VPCTN vs. DENA	0.05165	0.02995 to 0.07335	Yes	****
10	VPCTN vs. DENA/VPCTN	0.01984	-0.001855 to 0.04154	No	ns
11	DENA vs. DENA/VPCTN	-0.03181	-0.05351 to -0.01011	Yes	**
12					
13	**Test details**	**Mean 1**	**Mean 2**	**Mean Diff.**	**SE of diff.**
14	Normal vs. VPCTN	0.1028	0.09470	0.008120	0.007752
15	Normal vs. DENA	0.1028	0.04305	0.05977	0.007752
16	Normal vs. DENA/VPCTN	0.1028	0.07486	0.02796	0.007752
17	VPCTN vs. DENA	0.09470	0.04305	0.05165	0.007752
18	VPCTN vs. DENA/VPCTN	0.09470	0.07486	0.01984	0.007752
19	DENA vs. DENA/VPCTN	0.04305	0.07486	-0.03181	0.007752

1				
2				
3				
4				
5	**Adjusted P Value**			
6	0.7242	A-B		
7	<0.0001	A-C		
8	0.0088	A-D		
9	<0.0001	B-C		
10	0.0806	B-D		
11	0.0029	C-D		
12				
13	**n1**	**n2**	**q**	**DF**
14	6	6	1.481	20
15	6	6	10.90	20
16	6	6	5.101	20
17	6	6	9.423	20
18	6	6	3.620	20
19	6	6	5.803	20

Ordinary one-way ANOVA<br>ANOVA results					
1	Table Analyzed	ROS			
2	Data sets analyzed	A-D			
3					
4	**ANOVA summary**				
5	F	58.02			
6	P value	<0.0001			
7	P value summary	****			
8	Significant diff. among means (P < 0.05)?	Yes			
9	R squared	0.8969			
10					
11	**Brown-Forsythe test**				
12	F (DFn, DFd)	6.618 (3, 20)			
13	P value	0.0028			
14	P value summary	**			
15	Are SDs significantly different (P < 0.05)?	Yes			
16					
17	**Bartlett's test**				
18	Bartlett's statistic (corrected)	15.85			
19	P value	0.0012			
20	P value summary	**			
21	Are SDs significantly different (P < 0.05)?	Yes			
22					
23	**ANOVA table**	**SS**	**DF**	**MS**	**F (DFn, DFd)**
24	Treatment (between columns)	28041	3	9347	F (3, 20) = 58.02
25	Residual (within columns)	3222	20	161.1	
26	Total	31263	23		
27					
28	**Data summary**				
29	Number of treatments (columns)	4			
30	Number of values (total)	24			

1	
2	
3	
4	
5	
6	
7	
8	
9	
10	
11	
12	
13	
14	
15	
16	
17	
18	
19	
20	
21	
22	
23	**P value**
24	P<0.0001
25	
26	
27	
28	
29	
30	

Ordinary one-way ANOVA<br>Multiple comparisons					
1	Number of families	1			
2	Number of comparisons per family	6			
3	Alpha	0.05			
4					
5	**Tukey's multiple comparisons test**	**Mean Diff.**	**95.00% CI of diff.**	**Below threshold?**	**Summary**
6	Normal vs. VPCTN	2.437	-18.07 to 22.95	No	ns
7	Normal vs. DENA	-82.45	-103.0 to -61.93	Yes	****
8	Normal vs. DENA/VPCTN	-24.34	-44.85 to -3.828	Yes	*
9	VPCTN vs. DENA	-84.88	-105.4 to -64.37	Yes	****
10	VPCTN vs. DENA/VPCTN	-26.78	-47.29 to -6.265	Yes	**
11	DENA vs. DENA/VPCTN	58.11	37.60 to 78.62	Yes	****
12					
13	**Test details**	**Mean 1**	**Mean 2**	**Mean Diff.**	**SE of diff.**
14	Normal vs. VPCTN	22.43	20.00	2.437	7.328
15	Normal vs. DENA	22.43	104.9	-82.45	7.328
16	Normal vs. DENA/VPCTN	22.43	46.77	-24.34	7.328
17	VPCTN vs. DENA	20.00	104.9	-84.88	7.328
18	VPCTN vs. DENA/VPCTN	20.00	46.77	-26.78	7.328
19	DENA vs. DENA/VPCTN	104.9	46.77	58.11	7.328

1				
2				
3				
4				
5	**Adjusted P Value**			
6	0.9869	A-B		
7	<0.0001	A-C		
8	0.0165	A-D		
9	<0.0001	B-C		
10	0.0079	B-D		
11	<0.0001	C-D		
12				
13	**n1**	**n2**	**q**	**DF**
14	6	6	0.4702	20
15	6	6	15.91	20
16	6	6	4.697	20
17	6	6	16.38	20
18	6	6	5.167	20
19	6	6	11.21	20

	X	A	B
	Days	DENA	DENA/VPCTN
	X	Percentage	Percentage
1	0.000	100.000	100.000
2	40.000	91.667	
3	57.000	83.333	
4	66.000	75.000	
5	68.000		90.000
6	74.000	66.667	
7	78.000	58.333	
8	82.000	50.000	
9	84.000	50.000	90.000

	X	A	B
	Days	DENA	DENA/VPCTN
	X		
1	0.000	12	10
2	40.000	12	
3	57.000	11	
4	66.000	10	
5	68.000		10
6	74.000	9	
7	78.000	8	
8	82.000	7	
9	84.000	6	9

**Survival** Curve comparison		A	B
1	**Comparison of Survival Curves**		
2			
3	**Log-rank (Mantel-Cox) test**		
4	Chi square	3.730	
5	df	1	
6	P value	0.0534	
7	P value summary	ns	
8	Are the survival curves sig different?	No	
9			
10	**Median survival**		
11	DENA	83.00	
12	DENA/VPCTN	Undefined	
13			
14	**Hazard Ratio (Mantel-Haenszel)**	**A/B**	**B/A**
15	Ratio (and its reciprocal)	4.320	0.2315
16	95% CI of ratio	0.9785 to 19.08	0.05242 to 1.022
17			
18	**Hazard Ratio (logrank)**	**A/B**	**B/A**
19	Ratio (and its reciprocal)	6.170	0.1621
20	95% CI of ratio	1.402 to 27.15	0.03683 to 0.7132

**Survival**<br>Data summary		A<br>DENA	B<br>DENA/VPCTN
1	**Number of rows**	22	22
2	# of blank lines	10	12
3	# rows with impossible data	0	0
4	# censored subjects	6	9
5	# deaths/events	6	1
6			
7	**Median survival**	83	Undefined

Simple linear regression<br>Tabular results		A<br>% growth inh.
1	**Best-fit values**	
2	Slope	1.065
3	Y-intercept	8.475
4	X-intercept	-7.959
5	1/slope	0.9392
6		
7	**Std. Error**	
8	Slope	0.1577
9	Y-intercept	5.944
10		
11	**95% Confidence Intervals**	
12	Slope	0.6270 to 1.503
13	Y-intercept	-8.028 to 24.98
14	X-intercept	-35.64 to 5.972
15		
16	**Goodness of Fit**	
17	R squared	0.9193
18	Sy.x	10.60
19		
20	**Is slope significantly non-zero?**	
21	F	45.60
22	DFn, DFd	1, 4
23	P value	0.0025
24	Deviation from zero?	Significant
25		
26	**Equation**	Y = 1.065*X + 8.475
27		
28	**Data**	
29	Number of X values	6
30	Maximum number of Y replicates	1
31	Total number of values	6
32	Number of missing values	0

		X	A
		Conc. (Interpolated)	% growth inh. (Entered)
		X	
1		38.999	50.000

Ordinary one-way ANOVA<br>ANOVA results						
1	Table Analyzed	ALT				
2	Data sets analyzed	A-D				
3						
4	**ANOVA summary**					
5	F	116.6				
6	P value	<0.0001				
7	P value summary	****				
8	Significant diff. among means (P < 0.05)?	Yes				
9	R squared	0.9459				
10						
11	**Brown-Forsythe test**					
12	F (DFn, DFd)	4.324 (3, 20)				
13	P value	0.0167				
14	P value summary	*				
15	Are SDs significantly different (P < 0.05)?	Yes				
16						
17	**Bartlett's test**					
18	Bartlett's statistic (corrected)	26.91				
19	P value	<0.0001				
20	P value summary	****				
21	Are SDs significantly different (P < 0.05)?	Yes				
22						
23	**ANOVA table**	**SS**	**DF**	**MS**	**F (DFn, DFd)**	**P value**
24	Treatment (between columns)	185818	3	61939	F (3, 20) = 116.6	P<0.0001
25	Residual (within columns)	10624	20	531.2		
26	Total	196442	23			
27						
28	**Data summary**					
29	Number of treatments (columns)	4				
30	Number of values (total)	24				

Ordinary one-way ANOVA<br>Multiple comparisons					
1	Number of families	1			
2	Number of comparisons per family	6			
3	Alpha	0.05			
4					
5	**Tukey's multiple comparisons test**	**Mean Diff.**	**95.00% CI of diff.**	**Below threshold?**	**Summary**
6	Normal vs. VPCTN	-0.6633	-37.91 to 36.58	No	ns
7	Normal vs. DENA	-215.8	-253.1 to -178.6	Yes	****
8	Normal vs. DENA/VPCTN	-76.04	-113.3 to -38.80	Yes	****
9	VPCTN vs. DENA	-215.2	-252.4 to -177.9	Yes	****
10	VPCTN vs. DENA/VPCTN	-75.38	-112.6 to -38.13	Yes	****
11	DENA vs. DENA/VPCTN	139.8	102.5 to 177.0	Yes	****
12					
13	**Test details**	**Mean 1**	**Mean 2**	**Mean Diff.**	**SE of diff.**
14	Normal vs. VPCTN	28.99	29.65	-0.6633	13.31
15	Normal vs. DENA	28.99	244.8	-215.8	13.31
16	Normal vs. DENA/VPCTN	28.99	105.0	-76.04	13.31
17	VPCTN vs. DENA	29.65	244.8	-215.2	13.31
18	VPCTN vs. DENA/VPCTN	29.65	105.0	-75.38	13.31
19	DENA vs. DENA/VPCTN	244.8	105.0	139.8	13.31

1				
2				
3				
4				
5	**Adjusted P Value**			
6	>0.9999	A-B		
7	<0.0001	A-C		
8	<0.0001	A-D		
9	<0.0001	B-C		
10	<0.0001	B-D		
11	<0.0001	C-D		
12				
13	**n1**	**n2**	**q**	**DF**
14	6	6	0.07050	20
15	6	6	22.94	20
16	6	6	8.082	20
17	6	6	22.87	20
18	6	6	8.011	20
19	6	6	14.86	20

Ordinary one-way ANOVA
ANOVA results

1	Table Analyzed	AST				
2	Data sets analyzed	A-D				
3						
4	**ANOVA summary**					
5	F	191.6				
6	P value	<0.0001				
7	P value summary	****				
8	Significant diff. among means (P < 0.05)?	Yes				
9	R squared	0.9664				
10						
11	**Brown-Forsythe test**					
12	F (DFn, DFd)	2.357 (3, 20)				
13	P value	0.1023				
14	P value summary	ns				
15	Are SDs significantly different (P < 0.05)?	No				
16						
17	**Bartlett's test**					
18	Bartlett's statistic (corrected)	17.93				
19	P value	0.0005				
20	P value summary	***				
21	Are SDs significantly different (P < 0.05)?	Yes				
22						
23	**ANOVA table**	**SS**	**DF**	**MS**	**F (DFn, DFd)**	**P value**
24	Treatment (between columns)	447676	3	149225	F (3, 20) = 191.6	P<0.0001
25	Residual (within columns)	15574	20	778.7		
26	Total	463251	23			
27						
28	**Data summary**					
29	Number of treatments (columns)	4				
30	Number of values (total)	24				

Ordinary one-way ANOVA<br>Multiple comparisons					
1	Number of families	1			
2	Number of comparisons per family	6			
3	Alpha	0.05			
4					
5	**Tukey's multiple comparisons test**	**Mean Diff.**	**95.00% CI of diff.**	**Below threshold?**	**Summary**
6	Normal vs. VPCTN	2.550	-42.54 to 47.64	No	ns
7	Normal vs. DENA	-332.1	-377.2 to -287.0	Yes	****
8	Normal vs. DENA/VPCTN	-84.09	-129.2 to -39.00	Yes	***
9	VPCTN vs. DENA	-334.7	-379.8 to -289.6	Yes	****
10	VPCTN vs. DENA/VPCTN	-86.64	-131.7 to -41.55	Yes	***
11	DENA vs. DENA/VPCTN	248.1	203.0 to 293.1	Yes	****
12					
13	**Test details**	**Mean 1**	**Mean 2**	**Mean Diff.**	**SE of diff.**
14	Normal vs. VPCTN	58.84	56.29	2.550	16.11
15	Normal vs. DENA	58.84	391.0	-332.1	16.11
16	Normal vs. DENA/VPCTN	58.84	142.9	-84.09	16.11
17	VPCTN vs. DENA	56.29	391.0	-334.7	16.11
18	VPCTN vs. DENA/VPCTN	56.29	142.9	-86.64	16.11
19	DENA vs. DENA/VPCTN	391.0	142.9	248.1	16.11

1				
2				
3				
4				
5	**Adjusted P Value**			
6	0.9985	A-B		
7	<0.0001	A-C		
8	0.0002	A-D		
9	<0.0001	B-C		
10	0.0002	B-D		
11	<0.0001	C-D		
12				
13	**n1**	**n2**	**q**	**DF**
14	6	6	0.2238	20
15	6	6	29.15	20
16	6	6	7.381	20
17	6	6	29.38	20
18	6	6	7.605	20
19	6	6	21.77	20

Ordinary one-way ANOVA<br>ANOVA results						
1	Table Analyzed	GGT				
2	Data sets analyzed	A-D				
3						
4	**ANOVA summary**					
5	F	69.29				
6	P value	<0.0001				
7	P value summary	****				
8	Significant diff. among means (P < 0.05)?	Yes				
9	R squared	0.9122				
10						
11	**Brown-Forsythe test**					
12	F (DFn, DFd)	4.004 (3, 20)				
13	P value	0.0220				
14	P value summary	*				
15	Are SDs significantly different (P < 0.05)?	Yes				
16						
17	**Bartlett's test**					
18	Bartlett's statistic (corrected)	20.50				
19	P value	0.0001				
20	P value summary	***				
21	Are SDs significantly different (P < 0.05)?	Yes				
22						
23	**ANOVA table**	**SS**	**DF**	**MS**	**F (DFn, DFd)**	**P value**
24	Treatment (between columns)	17100	3	5700	F (3, 20) = 69.29	P<0.0001
25	Residual (within columns)	1645	20	82.26		
26	Total	18745	23			
27						
28	**Data summary**					
29	Number of treatments (columns)	4				
30	Number of values (total)	24				

Ordinary one-way ANOVA<br>Multiple comparisons					
1	Number of families	1			
2	Number of comparisons per family	6			
3	Alpha	0.05			
4					
5	**Tukey's multiple comparisons test**	**Mean Diff.**	**95.00% CI of diff.**	**Below threshold?**	**Summary**
6	Normal vs. VPCTN	-1.455	-16.11 to 13.20	No	ns
7	Normal vs. DENA	-62.74	-77.40 to -48.09	Yes	****
8	Normal vs. DENA/VPCTN	-40.89	-55.55 to -26.24	Yes	****
9	VPCTN vs. DENA	-61.29	-75.94 to -46.63	Yes	****
10	VPCTN vs. DENA/VPCTN	-39.44	-54.09 to -24.78	Yes	****
11	DENA vs. DENA/VPCTN	21.85	7.195 to 36.51	Yes	**
12					
13	**Test details**	**Mean 1**	**Mean 2**	**Mean Diff.**	**SE of diff.**
14	Normal vs. VPCTN	8.727	10.18	-1.455	5.236
15	Normal vs. DENA	8.727	71.47	-62.74	5.236
16	Normal vs. DENA/VPCTN	8.727	49.62	-40.89	5.236
17	VPCTN vs. DENA	10.18	71.47	-61.29	5.236
18	VPCTN vs. DENA/VPCTN	10.18	49.62	-39.44	5.236
19	DENA vs. DENA/VPCTN	71.47	49.62	21.85	5.236

1				
2				
3				
4				
5	**Adjusted P Value**			
6	0.9923	A-B		
7	<0.0001	A-C		
8	<0.0001	A-D		
9	<0.0001	B-C		
10	<0.0001	B-D		
11	0.0024	C-D		
12				
13	**n1**	**n2**	**q**	**DF**
14	6	6	0.3931	20
15	6	6	16.94	20
16	6	6	11.04	20
17	6	6	16.55	20
18	6	6	10.65	20
19	6	6	5.901	20

Mann-Whitney test		
1	Table Analyzed	Necroinflammation
2		
3	Column B	DENA/VPCTN
4	vs.	vs.
5	Column A	DENA
6		
7	**Mann Whitney test**	
8	P value	0.0022
9	Exact or approximate P value?	Exact
10	P value summary	**
11	Significantly different (P < 0.05)?	Yes
12	One- or two-tailed P value?	Two-tailed
13	Sum of ranks in column A,B	57 , 21
14	Mann-Whitney U	0
15		
16	**Difference between medians**	
17	Median of column A	9.500, n=6
18	Median of column B	3.000, n=6
19	Difference: Actual	-6.500
20	Difference: Hodges-Lehmann	-7.000

Ordinary one-way ANOVA<br>ANOVA results						
1	Table Analyzed	48h cell proliferation				
2	Data sets analyzed	A-F				
3						
4	**ANOVA summary**					
5	F	312.2				
6	P value	<0.0001				
7	P value summary	****				
8	Significant diff. among means (P < 0.05)?	Yes				
9	R squared	0.9924				
10						
11	**Brown-Forsythe test**					
12	F (DFn, DFd)					
13	P value					
14	P value summary					
15	Are SDs significantly different (P < 0.05)?					
16						
17	**Bartlett's test**					
18	Bartlett's statistic (corrected)					
19	P value					
20	P value summary					
21	Are SDs significantly different (P < 0.05)?					
22						
23	**ANOVA table**	**SS**	**DF**	**MS**	**F (DFn, DFd)**	**P value**
24	Treatment (between columns)	17366	5	3473	F (5, 12) = 312.2	P<0.0001
25	Residual (within columns)	133.5	12	11.13		
26	Total	17500	17			
27						
28	**Data summary**					
29	Number of treatments (columns)	6				
30	Number of values (total)	18				

**Ordinary one-way ANOVA**<br>Multiple comparisons					
1	Number of families	1			
2	Number of comparisons per family	15			
3	Alpha	0.05			
4					
5	**Tukey's multiple comparisons test**	**Mean Diff.**	**95.00% CI of diff.**	**Below threshold?**	**Summary**
6	0 vs. 5	13.86	4.712 to 23.01	Yes	**
7	0 vs. 10	25.39	16.24 to 34.54	Yes	****
8	0 vs. 20	47.80	38.65 to 56.95	Yes	****
9	0 vs. 40	71.51	62.36 to 80.66	Yes	****
10	0 vs. 80	87.37	78.22 to 96.52	Yes	****
11	5 vs. 10	11.53	2.380 to 20.68	Yes	*
12	5 vs. 20	33.94	24.79 to 43.09	Yes	****
13	5 vs. 40	57.65	48.50 to 66.80	Yes	****
14	5 vs. 80	73.51	64.36 to 82.66	Yes	****
15	10 vs. 20	22.41	13.27 to 31.56	Yes	****
16	10 vs. 40	46.12	36.98 to 55.27	Yes	****
17	10 vs. 80	61.98	52.83 to 71.13	Yes	****
18	20 vs. 40	23.71	14.56 to 32.86	Yes	****
19	20 vs. 80	39.57	30.42 to 48.72	Yes	****
20	40 vs. 80	15.86	6.710 to 25.01	Yes	***
21					
22	**Test details**	**Mean 1**	**Mean 2**	**Mean Diff.**	**SE of diff.**
23	0 vs. 5	100.0	86.14	13.86	2.723
24	0 vs. 10	100.0	74.61	25.39	2.723
25	0 vs. 20	100.0	52.20	47.80	2.723
26	0 vs. 40	100.0	28.49	71.51	2.723
27	0 vs. 80	100.0	12.63	87.37	2.723
28	5 vs. 10	86.14	74.61	11.53	2.723
29	5 vs. 20	86.14	52.20	33.94	2.723
30	5 vs. 40	86.14	28.49	57.65	2.723
31	5 vs. 80	86.14	12.63	73.51	2.723
32	10 vs. 20	74.61	52.20	22.41	2.723
33	10 vs. 40	74.61	28.49	46.12	2.723
34	10 vs. 80	74.61	12.63	61.98	2.723
35	20 vs. 40	52.20	28.49	23.71	2.723
36	20 vs. 80	52.20	12.63	39.57	2.723
37	40 vs. 80	28.49	12.63	15.86	2.723

1				
2				
3				
4				
5	**Adjusted P Value**			
6	0.0028	A-B		
7	<0.0001	A-C		
8	<0.0001	A-D		
9	<0.0001	A-E		
10	<0.0001	A-F		
11	0.0115	B-C		
12	<0.0001	B-D		
13	<0.0001	B-E		
14	<0.0001	B-F		
15	<0.0001	C-D		
16	<0.0001	C-E		
17	<0.0001	C-F		
18	<0.0001	D-E		
19	<0.0001	D-F		
20	0.0009	E-F		
21				
22	**n1**	**n2**	**q**	**DF**
23	3	3	7.197	12
24	3	3	13.18	12
25	3	3	24.82	12
26	3	3	37.13	12
27	3	3	45.37	12
28	3	3	5.986	12
29	3	3	17.62	12
30	3	3	29.94	12
31	3	3	38.17	12
32	3	3	11.64	12
33	3	3	23.95	12
34	3	3	32.19	12
35	3	3	12.31	12
36	3	3	20.55	12
37	3	3	8.235	12

**Ordinary one-way ANOVA**<br>ANOVA results						
1	Table Analyzed	72h cell proliferation				
2	Data sets analyzed	A-F				
3						
4	**ANOVA summary**					
5	F	546.0				
6	P value	<0.0001				
7	P value summary	****				
8	Significant diff. among means (P < 0.05)?	Yes				
9	R squared	0.9956				
10						
11	**Brown-Forsythe test**					
12	F (DFn, DFd)					
13	P value					
14	P value summary					
15	Are SDs significantly different (P < 0.05)?					
16						
17	**Bartlett's test**					
18	Bartlett's statistic (corrected)					
19	P value					
20	P value summary					
21	Are SDs significantly different (P < 0.05)?					
22						
23	**ANOVA table**	**SS**	**DF**	**MS**	**F (DFn, DFd)**	**P value**
24	Treatment (between columns)	17336	5	3467	F (5, 12) = 546.0	P<0.0001
25	Residual (within columns)	76.20	12	6.350		
26	Total	17412	17			
27						
28	**Data summary**					
29	Number of treatments (columns)	6				
30	Number of values (total)	18				

**Ordinary one-way ANOVA**<br>Multiple comparisons					
1	Number of families	1			
2	Number of comparisons per family	15			
3	Alpha	0.05			
4					
5	**Tukey's multiple comparisons test**	**Mean Diff.**	**95.00% CI of diff.**	**Below threshold?**	**Summary**
6	0 vs. 5	27.86	20.95 to 34.77	Yes	****
7	0 vs. 10	36.68	29.76 to 43.59	Yes	****
8	0 vs. 20	56.80	49.89 to 63.71	Yes	****
9	0 vs. 40	77.95	71.03 to 84.86	Yes	****
10	0 vs. 80	92.37	85.46 to 99.28	Yes	****
11	5 vs. 10	8.816	1.905 to 15.73	Yes	*
12	5 vs. 20	28.94	22.03 to 35.85	Yes	****
13	5 vs. 40	50.09	43.17 to 57.00	Yes	****
14	5 vs. 80	64.51	57.60 to 71.42	Yes	****
15	10 vs. 20	20.13	13.21 to 27.04	Yes	****
16	10 vs. 40	41.27	34.36 to 48.18	Yes	****
17	10 vs. 80	55.69	48.78 to 62.61	Yes	****
18	20 vs. 40	21.15	14.23 to 28.06	Yes	****
19	20 vs. 80	35.57	28.66 to 42.48	Yes	****
20	40 vs. 80	14.42	7.513 to 21.34	Yes	***
21					
22	**Test details**	**Mean 1**	**Mean 2**	**Mean Diff.**	**SE of diff.**
23	0 vs. 5	100.0	72.14	27.86	2.058
24	0 vs. 10	100.0	63.32	36.68	2.058
25	0 vs. 20	100.0	43.20	56.80	2.058
26	0 vs. 40	100.0	22.05	77.95	2.058
27	0 vs. 80	100.0	7.630	92.37	2.058
28	5 vs. 10	72.14	63.32	8.816	2.058
29	5 vs. 20	72.14	43.20	28.94	2.058
30	5 vs. 40	72.14	22.05	50.09	2.058
31	5 vs. 80	72.14	7.630	64.51	2.058
32	10 vs. 20	63.32	43.20	20.13	2.058
33	10 vs. 40	63.32	22.05	41.27	2.058
34	10 vs. 80	63.32	7.630	55.69	2.058
35	20 vs. 40	43.20	22.05	21.15	2.058
36	20 vs. 80	43.20	7.630	35.57	2.058
37	40 vs. 80	22.05	7.630	14.42	2.058

1				
2				
3				
4				
5	**Adjusted P Value**			
6	<0.0001	A-B		
7	<0.0001	A-C		
8	<0.0001	A-D		
9	<0.0001	A-E		
10	<0.0001	A-F		
11	0.0105	B-C		
12	<0.0001	B-D		
13	<0.0001	B-E		
14	<0.0001	B-F		
15	<0.0001	C-D		
16	<0.0001	C-E		
17	<0.0001	C-F		
18	<0.0001	D-E		
19	<0.0001	D-F		
20	0.0002	E-F		
21				
22	**n1**	**n2**	**q**	**DF**
23	3	3	19.15	12
24	3	3	25.21	12
25	3	3	39.04	12
26	3	3	53.58	12
27	3	3	63.49	12
28	3	3	6.060	12
29	3	3	19.89	12
30	3	3	34.43	12
31	3	3	44.34	12
32	3	3	13.83	12
33	3	3	28.37	12
34	3	3	38.28	12
35	3	3	14.53	12
36	3	3	24.45	12
37	3	3	9.914	12

Ordinary one-way ANOVA<br>ANOVA results						
1	Table Analyzed	24h cell proliferation				
2	Data sets analyzed	A-F				
3						
4	**ANOVA summary**					
5	F	468.8				
6	P value	<0.0001				
7	P value summary	****				
8	Significant diff. among means (P < 0.05)?	Yes				
9	R squared	0.9949				
10						
11	**Brown-Forsythe test**					
12	F (DFn, DFd)					
13	P value					
14	P value summary					
15	Are SDs significantly different (P < 0.05)?					
16						
17	**Bartlett's test**					
18	Bartlett's statistic (corrected)					
19	P value					
20	P value summary					
21	Are SDs significantly different (P < 0.05)?					
22						
23	**ANOVA table**	**SS**	**DF**	**MS**	**F (DFn, DFd)**	**P value**
24	Treatment (between columns)	16761	5	3352	F (5, 12) = 468.8	P<0.0001
25	Residual (within columns)	85.82	12	7.151		
26	Total	16847	17			
27						
28	**Data summary**					
29	Number of treatments (columns)	6				
30	Number of values (total)	18				

Ordinary one-way ANOVA<br>Multiple comparisons					
1	Number of families	1			
2	Number of comparisons per family	15			
3	Alpha	0.05			
4					
5	**Tukey's multiple comparisons test**	**Mean Diff.**	**95.00% CI of diff.**	**Below threshold?**	**Summary**
6	0 vs. 5	9.472	2.138 to 16.81	Yes	**
7	0 vs. 10	17.15	9.816 to 24.48	Yes	****
8	0 vs. 20	39.88	32.55 to 47.21	Yes	****
9	0 vs. 40	64.60	57.27 to 71.93	Yes	****
10	0 vs. 80	84.98	77.65 to 92.31	Yes	****
11	5 vs. 10	7.678	0.3435 to 15.01	Yes	*
12	5 vs. 20	30.41	23.07 to 37.74	Yes	****
13	5 vs. 40	55.13	47.79 to 62.46	Yes	****
14	5 vs. 80	75.51	68.17 to 82.84	Yes	****
15	10 vs. 20	22.73	15.40 to 30.06	Yes	****
16	10 vs. 40	47.45	40.12 to 54.78	Yes	****
17	10 vs. 80	67.83	60.50 to 75.16	Yes	****
18	20 vs. 40	24.72	17.39 to 32.05	Yes	****
19	20 vs. 80	45.10	37.77 to 52.43	Yes	****
20	40 vs. 80	20.38	13.05 to 27.71	Yes	****
21					
22	**Test details**	**Mean 1**	**Mean 2**	**Mean Diff.**	**SE of diff.**
23	0 vs. 5	100.0	90.53	9.472	2.183
24	0 vs. 10	100.0	82.85	17.15	2.183
25	0 vs. 20	100.0	60.12	39.88	2.183
26	0 vs. 40	100.0	35.40	64.60	2.183
27	0 vs. 80	100.0	15.02	84.98	2.183
28	5 vs. 10	90.53	82.85	7.678	2.183
29	5 vs. 20	90.53	60.12	30.41	2.183
30	5 vs. 40	90.53	35.40	55.13	2.183
31	5 vs. 80	90.53	15.02	75.51	2.183
32	10 vs. 20	82.85	60.12	22.73	2.183
33	10 vs. 40	82.85	35.40	47.45	2.183
34	10 vs. 80	82.85	15.02	67.83	2.183
35	20 vs. 40	60.12	35.40	24.72	2.183
36	20 vs. 80	60.12	15.02	45.10	2.183
37	40 vs. 80	35.40	15.02	20.38	2.183

1				
2				
3				
4				
5	**Adjusted P Value**			
6	0.0096	A-B		
7	<0.0001	A-C		
8	<0.0001	A-D		
9	<0.0001	A-E		
10	<0.0001	A-F		
11	0.0384	B-C		
12	<0.0001	B-D		
13	<0.0001	B-E		
14	<0.0001	B-F		
15	<0.0001	C-D		
16	<0.0001	C-E		
17	<0.0001	C-F		
18	<0.0001	D-E		
19	<0.0001	D-F		
20	<0.0001	E-F		
21				
22	**n1**	**n2**	**q**	**DF**
23	3	3	6.135	12
24	3	3	11.11	12
25	3	3	25.83	12
26	3	3	41.84	12
27	3	3	55.04	12
28	3	3	4.973	12
29	3	3	19.69	12
30	3	3	35.71	12
31	3	3	48.91	12
32	3	3	14.72	12
33	3	3	30.73	12
34	3	3	43.93	12
35	3	3	16.01	12
36	3	3	29.21	12
37	3	3	13.20	12

**Ordinary one-way ANOVA**<br>ANOVA results						
1	Table Analyzed	48h cell proliferation				
2	Data sets analyzed	A-F				
3						
4	**ANOVA summary**					
5	F	312.2				
6	P value	<0.0001				
7	P value summary	****				
8	Significant diff. among means (P < 0.05)?	Yes				
9	R squared	0.9924				
10						
11	**Brown-Forsythe test**					
12	F (DFn, DFd)					
13	P value					
14	P value summary					
15	Are SDs significantly different (P < 0.05)?					
16						
17	**Bartlett's test**					
18	Bartlett's statistic (corrected)					
19	P value					
20	P value summary					
21	Are SDs significantly different (P < 0.05)?					
22						
23	**ANOVA table**	**SS**	**DF**	**MS**	**F (DFn, DFd)**	**P value**
24	Treatment (between columns)	17366	5	3473	F (5, 12) = 312.2	P<0.0001
25	Residual (within columns)	133.5	12	11.13		
26	Total	17500	17			
27						
28	**Data summary**					
29	Number of treatments (columns)	6				
30	Number of values (total)	18				

Ordinary one-way ANOVA<br>Multiple comparisons					
1	Number of families	1			
2	Number of comparisons per family	15			
3	Alpha	0.05			
4					
5	**Tukey's multiple comparisons test**	**Mean Diff.**	**95.00% CI of diff.**	**Below threshold?**	**Summary**
6	0 vs. 5	13.86	4.712 to 23.01	Yes	**
7	0 vs. 10	25.39	16.24 to 34.54	Yes	****
8	0 vs. 20	47.80	38.65 to 56.95	Yes	****
9	0 vs. 40	71.51	62.36 to 80.66	Yes	****
10	0 vs. 80	87.37	78.22 to 96.52	Yes	****
11	5 vs. 10	11.53	2.380 to 20.68	Yes	*
12	5 vs. 20	33.94	24.79 to 43.09	Yes	****
13	5 vs. 40	57.65	48.50 to 66.80	Yes	****
14	5 vs. 80	73.51	64.36 to 82.66	Yes	****
15	10 vs. 20	22.41	13.27 to 31.56	Yes	****
16	10 vs. 40	46.12	36.98 to 55.27	Yes	****
17	10 vs. 80	61.98	52.83 to 71.13	Yes	****
18	20 vs. 40	23.71	14.56 to 32.86	Yes	****
19	20 vs. 80	39.57	30.42 to 48.72	Yes	****
20	40 vs. 80	15.86	6.710 to 25.01	Yes	***
21					
22	**Test details**	**Mean 1**	**Mean 2**	**Mean Diff.**	**SE of diff.**
23	0 vs. 5	100.0	86.14	13.86	2.723
24	0 vs. 10	100.0	74.61	25.39	2.723
25	0 vs. 20	100.0	52.20	47.80	2.723
26	0 vs. 40	100.0	28.49	71.51	2.723
27	0 vs. 80	100.0	12.63	87.37	2.723
28	5 vs. 10	86.14	74.61	11.53	2.723
29	5 vs. 20	86.14	52.20	33.94	2.723
30	5 vs. 40	86.14	28.49	57.65	2.723
31	5 vs. 80	86.14	12.63	73.51	2.723
32	10 vs. 20	74.61	52.20	22.41	2.723
33	10 vs. 40	74.61	28.49	46.12	2.723
34	10 vs. 80	74.61	12.63	61.98	2.723
35	20 vs. 40	52.20	28.49	23.71	2.723
36	20 vs. 80	52.20	12.63	39.57	2.723
37	40 vs. 80	28.49	12.63	15.86	2.723

1				
2				
3				
4				
5	**Adjusted P Value**			
6	0.0028	A-B		
7	<0.0001	A-C		
8	<0.0001	A-D		
9	<0.0001	A-E		
10	<0.0001	A-F		
11	0.0115	B-C		
12	<0.0001	B-D		
13	<0.0001	B-E		
14	<0.0001	B-F		
15	<0.0001	C-D		
16	<0.0001	C-E		
17	<0.0001	C-F		
18	<0.0001	D-E		
19	<0.0001	D-F		
20	0.0009	E-F		
21				
22	**n1**	**n2**	**q**	**DF**
23	3	3	7.197	12
24	3	3	13.18	12
25	3	3	24.82	12
26	3	3	37.13	12
27	3	3	45.37	12
28	3	3	5.986	12
29	3	3	17.62	12
30	3	3	29.94	12
31	3	3	38.17	12
32	3	3	11.64	12
33	3	3	23.95	12
34	3	3	32.19	12
35	3	3	12.31	12
36	3	3	20.55	12
37	3	3	8.235	12

Ordinary one-way ANOVA<br>ANOVA results						
1	Table Analyzed	72h cell proliferation				
2	Data sets analyzed	A-F				
3						
4	**ANOVA summary**					
5	F	546.0				
6	P value	<0.0001				
7	P value summary	****				
8	Significant diff. among means (P < 0.05)?	Yes				
9	R squared	0.9956				
10						
11	**Brown-Forsythe test**					
12	F (DFn, DFd)					
13	P value					
14	P value summary					
15	Are SDs significantly different (P < 0.05)?					
16						
17	**Bartlett's test**					
18	Bartlett's statistic (corrected)					
19	P value					
20	P value summary					
21	Are SDs significantly different (P < 0.05)?					
22						
23	**ANOVA table**	**SS**	**DF**	**MS**	**F (DFn, DFd)**	**P value**
24	Treatment (between columns)	17336	5	3467	F (5, 12) = 546.0	P<0.0001
25	Residual (within columns)	76.20	12	6.350		
26	Total	17412	17			
27						
28	**Data summary**					
29	Number of treatments (columns)	6				
30	Number of values (total)	18				

**Ordinary one-way ANOVA**<br>Multiple comparisons					
1	Number of families	1			
2	Number of comparisons per family	5			
3	Alpha	0.05			
4					
5	**Dunnett's multiple comparisons test**	**Mean Diff.**	**95.00% CI of diff.**	**Below threshold?**	**Summary**
6	0 vs. 5	27.86	21.89 to 33.83	Yes	****
7	0 vs. 10	36.68	30.71 to 42.65	Yes	****
8	0 vs. 20	56.80	50.83 to 62.77	Yes	****
9	0 vs. 40	77.95	71.98 to 83.92	Yes	****
10	0 vs. 80	92.37	86.40 to 98.34	Yes	****
11					
12	**Test details**	**Mean 1**	**Mean 2**	**Mean Diff.**	**SE of diff.**
13	0 vs. 5	100.0	72.14	27.86	2.058
14	0 vs. 10	100.0	63.32	36.68	2.058
15	0 vs. 20	100.0	43.20	56.80	2.058
16	0 vs. 40	100.0	22.05	77.95	2.058
17	0 vs. 80	100.0	7.630	92.37	2.058

1				
2				
3				
4				
5	**Adjusted P Value**	**A-?**		
6	<0.0001	B	5	
7	<0.0001	C	10	
8	<0.0001	D	20	
9	<0.0001	E	40	
10	<0.0001	F	80	
11				
12	**n1**	**n2**	**q**	**DF**
13	3	3	13.54	12
14	3	3	17.83	12
15	3	3	27.61	12
16	3	3	37.88	12
17	3	3	44.89	12

Ordinary one-way ANOVA<br>ANOVA results					
1	Table Analyzed	LDH media			
2	Data sets analyzed	A-F			
3					
4	**ANOVA summary**				
5	F	2.215			
6	P value	0.1203			
7	P value summary	ns			
8	Significant diff. among means (P < 0.05)?	No			
9	R squared	0.4799			
10					
11	**Brown-Forsythe test**				
12	F (DFn, DFd)	0.2098 (5, 12)			
13	P value	0.9519			
14	P value summary	ns			
15	Are SDs significantly different (P < 0.05)?	No			
16					
17	**Bartlett's test**				
18	Bartlett's statistic (corrected)				
19	P value				
20	P value summary				
21	Are SDs significantly different (P < 0.05)?				
22					
23	**ANOVA table**	**SS**	**DF**	**MS**	**F (DFn, DFd)**
24	Treatment (between columns)	55.03	5	11.01	F (5, 12) = 2.215
25	Residual (within columns)	59.64	12	4.970	
26	Total	114.7	17		
27					
28	**Data summary**				
29	Number of treatments (columns)	6			
30	Number of values (total)	18			

1	
2	
3	
4	
5	
6	
7	
8	
9	
10	
11	
12	
13	
14	
15	
16	
17	
18	
19	
20	
21	
22	
23	**P value**
24	P=0.1203
25	
26	
27	
28	
29	
30	

Ordinary one-way ANOVA<br>Multiple comparisons					
1	Number of families	1			
2	Number of comparisons per family	15			
3	Alpha	0.05			
4					
5	**Tukey's multiple comparisons test**	**Mean Diff.**	**95.00% CI of diff.**	**Below threshold?**	**Summary**
6	0 vs. 5	-0.3062	-6.420 to 5.808	No	ns
7	0 vs. 10	-0.09163	-6.206 to 6.022	No	ns
8	0 vs. 20	-1.961	-8.075 to 4.153	No	ns
9	0 vs. 40	-2.876	-8.991 to 3.238	No	ns
10	0 vs. 80	-4.779	-10.89 to 1.335	No	ns
11	5 vs. 10	0.2146	-5.900 to 6.329	No	ns
12	5 vs. 20	-1.655	-7.769 to 4.459	No	ns
13	5 vs. 40	-2.570	-8.684 to 3.544	No	ns
14	5 vs. 80	-4.473	-10.59 to 1.642	No	ns
15	10 vs. 20	-1.869	-7.984 to 4.245	No	ns
16	10 vs. 40	-2.785	-8.899 to 3.329	No	ns
17	10 vs. 80	-4.687	-10.80 to 1.427	No	ns
18	20 vs. 40	-0.9154	-7.029 to 5.199	No	ns
19	20 vs. 80	-2.818	-8.932 to 3.296	No	ns
20	40 vs. 80	-1.902	-8.016 to 4.212	No	ns
21					
22	**Test details**	**Mean 1**	**Mean 2**	**Mean Diff.**	**SE of diff.**
23	0 vs. 5	15.79	16.10	-0.3062	1.820
24	0 vs. 10	15.79	15.88	-0.09163	1.820
25	0 vs. 20	15.79	17.75	-1.961	1.820
26	0 vs. 40	15.79	18.67	-2.876	1.820
27	0 vs. 80	15.79	20.57	-4.779	1.820
28	5 vs. 10	16.10	15.88	0.2146	1.820
29	5 vs. 20	16.10	17.75	-1.655	1.820
30	5 vs. 40	16.10	18.67	-2.570	1.820
31	5 vs. 80	16.10	20.57	-4.473	1.820
32	10 vs. 20	15.88	17.75	-1.869	1.820
33	10 vs. 40	15.88	18.67	-2.785	1.820
34	10 vs. 80	15.88	20.57	-4.687	1.820
35	20 vs. 40	17.75	18.67	-0.9154	1.820
36	20 vs. 80	17.75	20.57	-2.818	1.820
37	40 vs. 80	18.67	20.57	-1.902	1.820

1				
2				
3				
4				
5	**Adjusted P Value**			
6	>0.9999	A-B		
7	>0.9999	A-C		
8	0.8812	A-D		
9	0.6248	A-E		
10	0.1641	A-F		
11	>0.9999	B-C		
12	0.9368	B-D		
13	0.7201	B-E		
14	0.2115	B-F		
15	0.8999	C-D		
16	0.6537	C-E		
17	0.1773	C-F		
18	0.9951	D-E		
19	0.6434	D-F		
20	0.8935	E-F		
21				
22	**n1**	**n2**	**q**	**DF**
23	3	3	0.2379	12
24	3	3	0.07119	12
25	3	3	1.524	12
26	3	3	2.235	12
27	3	3	3.713	12
28	3	3	0.1667	12
29	3	3	1.286	12
30	3	3	1.997	12
31	3	3	3.475	12
32	3	3	1.452	12
33	3	3	2.164	12
34	3	3	3.642	12
35	3	3	0.7112	12
36	3	3	2.189	12
37	3	3	1.478	12

**Ordinary one-way ANOVA**<br>ANOVA results					
1	Table Analyzed	CCND1 HepG2 24h			
2	Data sets analyzed	A-C			
3					
4	**ANOVA summary**				
5	F	27.76			
6	P value	0.0009			
7	P value summary	***			
8	Significant diff. among means (P < 0.05)?	Yes			
9	R squared	0.9025			
10					
11	**Brown-Forsythe test**				
12	F (DFn, DFd)	0.006750 (2, 6)			
13	P value	0.9933			
14	P value summary	ns			
15	Are SDs significantly different (P < 0.05)?	No			
16					
17	**Bartlett's test**				
18	Bartlett's statistic (corrected)				
19	P value				
20	P value summary				
21	Are SDs significantly different (P < 0.05)?				
22					
23	**ANOVA table**	**SS**	**DF**	**MS**	**F (DFn, DFd)**
24	Treatment (between columns)	1.268	2	0.6339	F (2, 6) = 27.76
25	Residual (within columns)	0.1370	6	0.02284	
26	Total	1.405	8		
27					
28	**Data summary**				
29	Number of treatments (columns)	3			
30	Number of values (total)	9			

1	
2	
3	
4	
5	
6	
7	
8	
9	
10	
11	
12	
13	
14	
15	
16	
17	
18	
19	
20	
21	
22	
23	**P value**
24	P=0.0009
25	
26	
27	
28	
29	
30	

**Ordinary one-way ANOVA**<br>Multiple comparisons					
1	Number of families	1			
2	Number of comparisons per family	3			
3	Alpha	0.05			
4					
5	**Tukey's multiple comparisons test**	**Mean Diff.**	**95.00% CI of diff.**	**Below threshold?**	**Summary**
6	0 vs. 10	0.4327	0.05408 to 0.8113	Yes	*
7	0 vs. 20	0.9188	0.5402 to 1.297	Yes	***
8	10 vs. 20	0.4861	0.1075 to 0.8647	Yes	*
9					
10	**Test details**	**Mean 1**	**Mean 2**	**Mean Diff.**	**SE of diff.**
11	0 vs. 10	3.437	3.005	0.4327	0.1234
12	0 vs. 20	3.437	2.519	0.9188	0.1234
13	10 vs. 20	3.005	2.519	0.4861	0.1234

1				
2				
3				
4				
5	**Adjusted P Value**			
6	0.0295	A-B		
7	0.0007	A-C		
8	0.0179	B-C		
9				
10	**n1**	**n2**	**q**	**DF**
11	3	3	4.959	6
12	3	3	10.53	6
13	3	3	5.572	6

Ordinary one-way ANOVA<br>ANOVA results					
1	Table Analyzed	caspase-3 HepG2 24h			
2	Data sets analyzed	A-C			
3					
4	**ANOVA summary**				
5	F	44.19			
6	P value	0.0003			
7	P value summary	***			
8	Significant diff. among means (P < 0.05)?	Yes			
9	R squared	0.9364			
10					
11	**Brown-Forsythe test**				
12	F (DFn, DFd)	0.004219 (2, 6)			
13	P value	0.9958			
14	P value summary	ns			
15	Are SDs significantly different (P < 0.05)?	No			
16					
17	**Bartlett's test**				
18	Bartlett's statistic (corrected)				
19	P value				
20	P value summary				
21	Are SDs significantly different (P < 0.05)?				
22					
23	**ANOVA table**	**SS**	**DF**	**MS**	**F (DFn, DFd)**
24	Treatment (between columns)	7.027	2	3.513	F (2, 6) = 44.19
25	Residual (within columns)	0.4770	6	0.07950	
26	Total	7.504	8		
27					
28	**Data summary**				
29	Number of treatments (columns)	3			
30	Number of values (total)	9			

1	
2	
3	
4	
5	
6	
7	
8	
9	
10	
11	
12	
13	
14	
15	
16	
17	
18	
19	
20	
21	
22	
23	**P value**
24	P=0.0003
25	
26	
27	
28	
29	
30	

**Ordinary one-way ANOVA**<br>Multiple comparisons					
1	Number of families	1			
2	Number of comparisons per family	3			
3	Alpha	0.05			
4					
5	**Tukey's multiple comparisons test**	**Mean Diff.**	**95.00% CI of diff.**	**Below threshold?**	**Summary**
6	0 vs. 10	-0.9869	-1.693 to -0.2805	Yes	*
7	0 vs. 20	-2.162	-2.868 to -1.455	Yes	***
8	10 vs. 20	-1.175	-1.881 to -0.4684	Yes	**
9					
10	**Test details**	**Mean 1**	**Mean 2**	**Mean Diff.**	**SE of diff.**
11	0 vs. 10	3.563	4.550	-0.9869	0.2302
12	0 vs. 20	3.563	5.725	-2.162	0.2302
13	10 vs. 20	4.550	5.725	-1.175	0.2302

1				
2				
3				
4				
5	**Adjusted P Value**			
6	0.0122	A-B		
7	0.0002	A-C		
8	0.0053	B-C		
9				
10	**n1**	**n2**	**q**	**DF**
11	3	3	6.062	6
12	3	3	13.28	6
13	3	3	7.216	6

Ordinary one-way ANOVA<br>ANOVA results						
1	Table Analyzed	IL-6				
2	Data sets analyzed	A-D				
3						
4	**ANOVA summary**					
5	F	96.96				
6	P value	<0.0001				
7	P value summary	****				
8	Significant diff. among means (P < 0.05)?	Yes				
9	R squared	0.9357				
10						
11	**Brown-Forsythe test**					
12	F (DFn, DFd)	3.591 (3, 20)				
13	P value	0.0318				
14	P value summary	*				
15	Are SDs significantly different (P < 0.05)?	Yes				
16						
17	**Bartlett's test**					
18	Bartlett's statistic (corrected)	23.09				
19	P value	<0.0001				
20	P value summary	****				
21	Are SDs significantly different (P < 0.05)?	Yes				
22						
23	**ANOVA table**	**SS**	**DF**	**MS**	**F (DFn, DFd)**	**P value**
24	Treatment (between columns)	1268	3	422.7	F (3, 20) = 96.96	P<0.0001
25	Residual (within columns)	87.20	20	4.360		
26	Total	1355	23			
27						
28	**Data summary**					
29	Number of treatments (columns)	4				
30	Number of values (total)	24				

**Ordinary one-way ANOVA**<br>Multiple comparisons					
1	Number of families	1			
2	Number of comparisons per family	6			
3	Alpha	0.05			
4					
5	**Tukey's multiple comparisons test**	**Mean Diff.**	**95.00% CI of diff.**	**Below threshold?**	**Summary**
6	Normal vs. VPCTN	0.5782	-2.796 to 3.953	No	ns
7	Normal vs. DENA	-17.51	-20.88 to -14.13	Yes	****
8	Normal vs. DENA/VPCTN	-6.000	-9.374 to -2.626	Yes	***
9	VPCTN vs. DENA	-18.08	-21.46 to -14.71	Yes	****
10	VPCTN vs. DENA/VPCTN	-6.578	-9.952 to -3.204	Yes	***
11	DENA vs. DENA/VPCTN	11.51	8.132 to 14.88	Yes	****
12					
13	**Test details**	**Mean 1**	**Mean 2**	**Mean Diff.**	**SE of diff.**
14	Normal vs. VPCTN	5.616	5.038	0.5782	1.206
15	Normal vs. DENA	5.616	23.12	-17.51	1.206
16	Normal vs. DENA/VPCTN	5.616	11.62	-6.000	1.206
17	VPCTN vs. DENA	5.038	23.12	-18.08	1.206
18	VPCTN vs. DENA/VPCTN	5.038	11.62	-6.578	1.206
19	DENA vs. DENA/VPCTN	23.12	11.62	11.51	1.206

1				
2				
3				
4				
5	**Adjusted P Value**			
6	0.9627	A-B		
7	<0.0001	A-C		
8	0.0004	A-D		
9	<0.0001	B-C		
10	0.0001	B-D		
11	<0.0001	C-D		
12				
13	**n1**	**n2**	**q**	**DF**
14	6	6	0.6783	20
15	6	6	20.54	20
16	6	6	7.038	20
17	6	6	21.21	20
18	6	6	7.717	20
19	6	6	13.50	20

Ordinary one-way ANOVA
ANOVA results

1	Table Analyzed	VEGF				
2	Data sets analyzed	A-D				
3						
4	**ANOVA summary**					
5	F	45.67				
6	P value	<0.0001				
7	P value summary	****				
8	Significant diff. among means (P < 0.05)?	Yes				
9	R squared	0.8726				
10						
11	**Brown-Forsythe test**					
12	F (DFn, DFd)	3.294 (3, 20)				
13	P value	0.0417				
14	P value summary	*				
15	Are SDs significantly different (P < 0.05)?	Yes				
16						
17	**Bartlett's test**					
18	Bartlett's statistic (corrected)	21.16				
19	P value	<0.0001				
20	P value summary	****				
21	Are SDs significantly different (P < 0.05)?	Yes				
22						
23	**ANOVA table**	**SS**	**DF**	**MS**	**F (DFn, [illegible]**	**P value**
24	Treatment (between columns)	183554	3	61185	F (3, 20)	P<0.000[illegible]
25	Residual (within columns)	26796	20	1340		
26	Total	210350	23			
27						
28	**Data summary**					
29	Number of treatments (columns)	4				
30	Number of values (total)	24				

Ordinary one-way ANOVA<br>Multiple comparisons					
1	Number of families	1			
2	Number of comparisons per family	6			
3	Alpha	0.05			
4					
5	**Tukey's multiple comparisons test**	**Mean Diff.**	**95.00% CI of diff.**	**Below threshold?**	**Summary**
6	Normal vs. VPCTN	0.1475	-59.00 to 59.30	No	ns
7	Normal vs. DENA	-214.0	-273.1 to -154.9	Yes	****
8	Normal vs. DENA/VPCTN	-63.93	-123.1 to -4.782	Yes	*
9	VPCTN vs. DENA	-214.1	-273.3 to -155.0	Yes	****
10	VPCTN vs. DENA/VPCTN	-64.08	-123.2 to -4.929	Yes	*
11	DENA vs. DENA/VPCTN	150.1	90.92 to 209.2	Yes	****
12					
13	**Test details**	**Mean 1**	**Mean 2**	**Mean Diff.**	**SE of diff.**
14	Normal vs. VPCTN	78.16	78.01	0.1475	21.13
15	Normal vs. DENA	78.16	292.2	-214.0	21.13
16	Normal vs. DENA/VPCTN	78.16	142.1	-63.93	21.13
17	VPCTN vs. DENA	78.01	292.2	-214.1	21.13
18	VPCTN vs. DENA/VPCTN	78.01	142.1	-64.08	21.13
19	DENA vs. DENA/VPCTN	292.2	142.1	150.1	21.13

1				
2				
3				
4				
5	**Adjusted P Value**			
6	>0.9999	A-B		
7	<0.0001	A-C		
8	0.0312	A-D		
9	<0.0001	B-C		
10	0.0308	B-D		
11	<0.0001	C-D		
12				
13	**n1**	**n2**	**q**	**DF**
14	6	6	0.009869	20
15	6	6	14.32	20
16	6	6	4.278	20
17	6	6	14.33	20
18	6	6	4.288	20
19	6	6	10.04	20

Ordinary one-way ANOVA
ANOVA results

1	Table Analyzed	AFP				
2	Data sets analyzed	A-D				
3						
4	**ANOVA summary**					
5	F	36.77				
6	P value	<0.0001				
7	P value summary	****				
8	Significant diff. among means (P < 0.05)?	Yes				
9	R squared	0.8465				
10						
11	**Brown-Forsythe test**					
12	F (DFn, DFd)	9.919 (3, 20)				
13	P value	0.0003				
14	P value summary	***				
15	Are SDs significantly different (P < 0.05)?	Yes				
16						
17	**Bartlett's test**					
18	Bartlett's statistic (corrected)	12.68				
19	P value	0.0054				
20	P value summary	**				
21	Are SDs significantly different (P < 0.05)?	Yes				
22						
23	**ANOVA table**	**SS**	**DF**	**MS**	**F (DFn, DFd)**	**P value**
24	Treatment (between columns)	56.21	3	18.74	F (3, 20) = 36.77	P<0.0001
25	Residual (within columns)	10.19	20	0.5095		
26	Total	66.40	23			
27						
28	**Data summary**					
29	Number of treatments (columns)	4				
30	Number of values (total)	24				

**Ordinary one-way ANOVA**<br>Multiple comparisons					
1	Number of families	1			
2	Number of comparisons per family	6			
3	Alpha	0.05			
4					
5	**Tukey's multiple comparisons test**	**Mean Diff.**	**95.00% CI of diff.**	**Below threshold?**	**Summary**
6	Normal vs. VPCTN	0.07501	-1.078 to 1.229	No	ns
7	Normal vs. DENA	-3.648	-4.801 to -2.494	Yes	****
8	Normal vs. DENA/VPCTN	-1.835	-2.989 to -0.6818	Yes	**
9	VPCTN vs. DENA	-3.723	-4.876 to -2.569	Yes	****
10	VPCTN vs. DENA/VPCTN	-1.910	-3.064 to -0.7569	Yes	***
11	DENA vs. DENA/VPCTN	1.812	0.6589 to 2.966	Yes	**
12					
13	**Test details**	**Mean 1**	**Mean 2**	**Mean Diff.**	**SE of diff.**
14	Normal vs. VPCTN	1.436	1.361	0.07501	0.4121
15	Normal vs. DENA	1.436	5.083	-3.648	0.4121
16	Normal vs. DENA/VPCTN	1.436	3.271	-1.835	0.4121
17	VPCTN vs. DENA	1.361	5.083	-3.723	0.4121
18	VPCTN vs. DENA/VPCTN	1.361	3.271	-1.910	0.4121
19	DENA vs. DENA/VPCTN	5.083	3.271	1.812	0.4121

1				
2				
3				
4				
5	**Adjusted P Value**			
6	0.9978	A-B		
7	<0.0001	A-C		
8	0.0013	A-D		
9	<0.0001	B-C		
10	0.0008	B-D		
11	0.0015	C-D		
12				
13	**n1**	**n2**	**q**	**DF**
14	6	6	0.2574	20
15	6	6	12.52	20
16	6	6	6.298	20
17	6	6	12.77	20
18	6	6	6.555	20
19	6	6	6.219	20

Ordinary one-way ANOVA<br>ANOVA results						
1	Table Analyzed	liver weight index				
2	Data sets analyzed	A-D				
3						
4	**ANOVA summary**					
5	F	19.60				
6	P value	<0.0001				
7	P value summary	****				
8	Significant diff. among means (P < 0.05)?	Yes				
9	R squared	0.7462				
10						
11	**Brown-Forsythe test**					
12	F (DFn, DFd)	4.343 (3, 20)				
13	P value	0.0164				
14	P value summary	*				
15	Are SDs significantly different (P < 0.05)?	Yes				
16						
17	**Bartlett's test**					
18	Bartlett's statistic (corrected)	12.15				
19	P value	0.0069				
20	P value summary	**				
21	Are SDs significantly different (P < 0.05)?	Yes				
22						
23	**ANOVA table**	**SS**	**DF**	**MS**	**F (DFn, DFd)**	**P value**
24	Treatment (between columns)	0.0004743	3	0.0001581	F (3, 20) = 19.60	P<0.0001
25	Residual (within columns)	0.0001613	20	8.067e-006		
26	Total	0.0006356	23			
27						
28	**Data summary**					
29	Number of treatments (columns)	4				
30	Number of values (total)	24				

Ordinary one-way ANOVA<br>Multiple comparisons					
1	Number of families	1			
2	Number of comparisons per family	6			
3	Alpha	0.05			
4					
5	**Tukey's multiple comparisons t**	**Mean Diff.**	**95.00% CI of diff.**	**Below threshold?**	**Summary**
6	Normal vs. VPCTN	-0.001035	-0.005624 to 0.003555	No	ns
7	Normal vs. DENA	-0.01131	-0.01590 to -0.006724	Yes	****
8	Normal vs. DENA/VPCTN	-0.005148	-0.009738 to -0.0005584	Yes	*
9	VPCTN vs. DENA	-0.01028	-0.01487 to -0.005690	Yes	****
10	VPCTN vs. DENA/VPCTN	-0.004113	-0.008703 to 0.0004765	No	ns
11	DENA vs. DENA/VPCTN	0.006166	0.001576 to 0.01076	Yes	**
12					
13	**Test details**	**Mean 1**	**Mean 2**	**Mean Diff.**	**SE of diff.**
14	Normal vs. VPCTN	0.01350	0.01454	-0.001035	0.001640
15	Normal vs. DENA	0.01350	0.02482	-0.01131	0.001640
16	Normal vs. DENA/VPCTN	0.01350	0.01865	-0.005148	0.001640
17	VPCTN vs. DENA	0.01454	0.02482	-0.01028	0.001640
18	VPCTN vs. DENA/VPCTN	0.01454	0.01865	-0.004113	0.001640
19	DENA vs. DENA/VPCTN	0.02482	0.01865	0.006166	0.001640

1				
2				
3				
4				
5	**Adjusted P Value**			
6	0.9208	A-B		
7	<0.0001	A-C		
8	0.0245	A-D		
9	<0.0001	B-C		
10	0.0891	B-D		
11	0.0062	C-D		
12				
13	**n1**	**n2**	**q**	**DF**
14	6	6	0.8925	20
15	6	6	9.758	20
16	6	6	4.440	20
17	6	6	8.865	20
18	6	6	3.547	20
19	6	6	5.318	20

Ordinary one-way ANOVA<br>ANOVA results						
1	Table Analyzed	liver weight				
2	Data sets analyzed	A-D				
3						
4	**ANOVA summary**					
5	F	11.92				
6	P value	0.0001				
7	P value summary	***				
8	Significant diff. among means (P < 0.05)?	Yes				
9	R squared	0.6412				
10						
11	**Brown-Forsythe test**					
12	F (DFn, DFd)	1.140 (3, 20)				
13	P value	0.3571				
14	P value summary	ns				
15	Are SDs significantly different (P < 0.05)?	No				
16						
17	**Bartlett's test**					
18	Bartlett's statistic (corrected)	4.178				
19	P value	0.2429				
20	P value summary	ns				
21	Are SDs significantly different (P < 0.05)?	No				
22						
23	**ANOVA table**	**SS**	**DF**	**MS**	**F (DFn, DFd)**	**P value**
24	Treatment (between columns)	18.57	3	6.189	F (3, 20) = 11.92	P=0.0001
25	Residual (within columns)	10.39	20	0.5194		
26	Total	28.95	23			
27						
28	**Data summary**					
29	Number of treatments (columns)	4				
30	Number of values (total)	24				

**Ordinary one-way ANOVA** Multiple comparisons					
1	Number of families	1			
2	Number of comparisons per family	6			
3	Alpha	0.05			
4					
5	**Tukey's multiple comparisons test**	**Mean Diff.**	**95.00% CI of diff.**	**Below threshold?**	**Summary**
6	Normal vs. VPCTN	-0.2044	-1.369 to 0.9603	No	ns
7	Normal vs. DENA	-2.246	-3.411 to -1.082	Yes	***
8	Normal vs. DENA/VPCTN	-0.9206	-2.085 to 0.2440	No	ns
9	VPCTN vs. DENA	-2.042	-3.207 to -0.8775	Yes	***
10	VPCTN vs. DENA/VPCTN	-0.7163	-1.881 to 0.4484	No	ns
11	DENA vs. DENA/VPCTN	1.326	0.1613 to 2.490	Yes	*
12					
13	**Test details**	**Mean 1**	**Mean 2**	**Mean Diff.**	**SE of diff.**
14	Normal vs. VPCTN	4.439	4.644	-0.2044	0.4161
15	Normal vs. DENA	4.439	6.686	-2.246	0.4161
16	Normal vs. DENA/VPCTN	4.439	5.360	-0.9206	0.4161
17	VPCTN vs. DENA	4.644	6.686	-2.042	0.4161
18	VPCTN vs. DENA/VPCTN	4.644	5.360	-0.7163	0.4161
19	DENA vs. DENA/VPCTN	6.686	5.360	1.326	0.4161

1				
2				
3				
4				
5	**Adjusted P Value**			
6	0.9602	A-B		
7	0.0002	A-C		
8	0.1540	A-D		
9	0.0005	B-C		
10	0.3392	B-D		
11	0.0221	C-D		
12				
13	**n1**	**n2**	**q**	**DF**
14	6	6	0.6946	20
15	6	6	7.635	20
16	6	6	3.129	20
17	6	6	6.941	20
18	6	6	2.434	20
19	6	6	4.506	20

Ordinary one-way ANOVA<br>ANOVA results						
1	Table Analyzed	body weight				
2	Data sets analyzed	A-D				
3						
4	**ANOVA summary**					
5	F	8.307				
6	P value	0.0009				
7	P value summary	***				
8	Significant diff. among means (P < 0.05)?	Yes				
9	R squared	0.5548				
10						
11	**Brown-Forsythe test**					
12	F (DFn, DFd)	0.9314 (3, 20)				
13	P value	0.4438				
14	P value summary	ns				
15	Are SDs significantly different (P < 0.05)?	No				
16						
17	**Bartlett's test**					
18	Bartlett's statistic (corrected)	1.476				
19	P value	0.6879				
20	P value summary	ns				
21	Are SDs significantly different (P < 0.05)?	No				
22						
23	**ANOVA table**	**SS**	**DF**	**MS**	**F (DFn, DFd)**	**P value**
24	Treatment (between columns)	13064	3	4355	F (3, 20) = 8.307	P=0.0009
25	Residual (within columns)	10484	20	524.2		
26	Total	23548	23			
27						
28	**Data summary**					
29	Number of treatments (columns)	4				
30	Number of values (total)	24				

**Ordinary one-way ANOVA** Multiple comparisons					
1	Number of families	1			
2	Number of comparisons per family	6			
3	Alpha	0.05			
4					
5	**Tukey's multiple comparisons t**	**Mean Diff.**	**95.00% CI of diff.**	**Below threshold?**	**Summary**
6	Normal vs. VPCTN	7.058	-29.94 to 44.06	No	ns
7	Normal vs. DENA	57.35	20.35 to 94.34	Yes	**
8	Normal vs. DENA/VPCTN	38.65	1.656 to 75.65	Yes	*
9	VPCTN vs. DENA	50.29	13.29 to 87.29	Yes	**
10	VPCTN vs. DENA/VPCTN	31.60	-5.401 to 68.60	No	ns
11	DENA vs. DENA/VPCTN	-18.69	-55.69 to 18.31	No	ns
12					
13	**Test details**	**Mean 1**	**Mean 2**	**Mean Diff.**	**SE of diff.**
14	Normal vs. VPCTN	328.0	320.9	7.058	13.22
15	Normal vs. DENA	328.0	270.6	57.35	13.22
16	Normal vs. DENA/VPCTN	328.0	289.3	38.65	13.22
17	VPCTN vs. DENA	320.9	270.6	50.29	13.22
18	VPCTN vs. DENA/VPCTN	320.9	289.3	31.60	13.22
19	DENA vs. DENA/VPCTN	270.6	289.3	-18.69	13.22

1				
2				
3				
4				
5	**Adjusted P Value**			
6	0.9498	A-B		
7	0.0017	A-C		
8	0.0386	A-D		
9	0.0056	B-C		
10	0.1114	B-D		
11	0.5056	C-D		
12				
13	**n1**	**n2**	**q**	**DF**
14	6	6	0.7551	20
15	6	6	6.135	20
16	6	6	4.135	20
17	6	6	5.380	20
18	6	6	3.380	20
19	6	6	2.000	20

Ordinary one-way ANOVA<br>ANOVA results						
1	Table Analyzed	p-STAT3 OD				
2	Data sets analyzed	A-D				
3						
4	**ANOVA summary**					
5	F	17.50				
6	P value	<0.0001				
7	P value summary	****				
8	Significant diff. among means (P < 0.05)?	Yes				
9	R squared	0.7241				
10						
11	**Brown-Forsythe test**					
12	F (DFn, DFd)	1.932 (3, 20)				
13	P value	0.1569				
14	P value summary	ns				
15	Are SDs significantly different (P < 0.05)?	No				
16						
17	**Bartlett's test**					
18	Bartlett's statistic (corrected)	17.57				
19	P value	0.0005				
20	P value summary	***				
21	Are SDs significantly different (P < 0.05)?	Yes				
22						
23	**ANOVA table**	**SS**	**DF**	**MS**	**F (DFn, DFd)**	**P value**
24	Treatment (between columns)	0.1933	3	0.06443	F (3, 20) = 17.50	P<0.0001
25	Residual (within columns)	0.07364	20	0.003682		
26	Total	0.2669	23			
27						
28	**Data summary**					
29	Number of treatments (columns)	4				
30	Number of values (total)	24				

**Ordinary one-way ANOVA**<br>Multiple comparisons					
1	Number of families	1			
2	Number of comparisons per family	6			
3	Alpha	0.05			
4					
5	**Tukey's multiple comparisons test**	**Mean Diff.**	**95.00% CI of diff.**	**Below threshold?**	**Summary**
6	Normal vs. VPCTN	0.01418	-0.08388 to 0.1122	No	ns
7	Normal vs. DENA	-0.2120	-0.3100 to -0.1139	Yes	****
8	Normal vs. DENA/VPCTN	-0.07833	-0.1764 to 0.01972	No	ns
9	VPCTN vs. DENA	-0.2262	-0.3242 to -0.1281	Yes	****
10	VPCTN vs. DENA/VPCTN	-0.09251	-0.1906 to 0.005546	No	ns
11	DENA vs. DENA/VPCTN	0.1337	0.03560 to 0.2317	Yes	**
12					
13	**Test details**	**Mean 1**	**Mean 2**	**Mean Diff.**	**SE of diff.**
14	Normal vs. VPCTN	0.1639	0.1497	0.01418	0.03503
15	Normal vs. DENA	0.1639	0.3759	-0.2120	0.03503
16	Normal vs. DENA/VPCTN	0.1639	0.2422	-0.07833	0.03503
17	VPCTN vs. DENA	0.1497	0.3759	-0.2262	0.03503
18	VPCTN vs. DENA/VPCTN	0.1497	0.2422	-0.09251	0.03503
19	DENA vs. DENA/VPCTN	0.3759	0.2422	0.1337	0.03503

1				
2				
3				
4				
5	**Adjusted P Value**			
6	0.9770	A-B		
7	<0.0001	A-C		
8	0.1477	A-D		
9	<0.0001	B-C		
10	0.0688	B-D		
11	0.0055	C-D		
12				
13	**n1**	**n2**	**q**	**DF**
14	6	6	0.5723	20
15	6	6	8.558	20
16	6	6	3.162	20
17	6	6	9.130	20
18	6	6	3.734	20
19	6	6	5.395	20

	Ordinary one-way ANOVA<br>ANOVA results					
1	Table Analyzed	VEGF mRI				
2	Data sets analyzed	A-D				
3						
4	**ANOVA summary**					
5	F	90.45				
6	P value	<0.0001				
7	P value summary	****				
8	Significant diff. among means (P < 0.05)?	Yes				
9	R squared	0.9314				
10						
11	**Brown-Forsythe test**					
12	F (DFn, DFd)					
13	P value					
14	P value summary					
15	Are SDs significantly different (P < 0.05)?					
16						
17	**Bartlett's test**					
18	Bartlett's statistic (corrected)	18.42				
19	P value	0.0004				
20	P value summary	***				
21	Are SDs significantly different (P < 0.05)?	Yes				
22						
23	**ANOVA table**	**SS**	**DF**	**MS**	**F (DFn, DFd)**	**P value**
24	Treatment (between columns)	97.01	3	32.34	F (3, 20) = 90.45	P<0.0001
25	Residual (within columns)	7.149	20	0.3575		
26	Total	104.2	23			
27						
28	**Data summary**					
29	Number of treatments (columns)	4				
30	Number of values (total)	24				

Ordinary one-way ANOVA<br>Multiple comparisons					
1	Number of families	1			
2	Number of comparisons per family	6			
3	Alpha	0.05			
4					
5	**Tukey's multiple comparisons t**	**Mean Diff.**	**95.00% CI of diff.**	**Below threshold?**	**Summary**
6	Normal vs. VPCTN	0.06112	-0.9050 to 1.027	No	ns
7	Normal vs. DENA	-4.893	-5.859 to -3.927	Yes	****
8	Normal vs. DENA/VPCTN	-1.690	-2.656 to -0.7238	Yes	***
9	VPCTN vs. DENA	-4.954	-5.920 to -3.988	Yes	****
10	VPCTN vs. DENA/VPCTN	-1.751	-2.717 to -0.7850	Yes	***
11	DENA vs. DENA/VPCTN	3.203	2.237 to 4.169	Yes	****
12					
13	**Test details**	**Mean 1**	**Mean 2**	**Mean Diff.**	**SE of diff.**
14	Normal vs. VPCTN	1.000	0.9389	0.06112	0.3452
15	Normal vs. DENA	1.000	5.893	-4.893	0.3452
16	Normal vs. DENA/VPCTN	1.000	2.690	-1.690	0.3452
17	VPCTN vs. DENA	0.9389	5.893	-4.954	0.3452
18	VPCTN vs. DENA/VPCTN	0.9389	2.690	-1.751	0.3452
19	DENA vs. DENA/VPCTN	5.893	2.690	3.203	0.3452

1				
2				
3				
4				
5	**Adjusted P Value**			
6	0.9980	A-B		
7	<0.0001	A-C		
8	0.0005	A-D		
9	<0.0001	B-C		
10	0.0003	B-D		
11	<0.0001	C-D		
12				
13	**n1**	**n2**	**q**	**DF**
14	6	6	0.2504	20
15	6	6	20.05	20
16	6	6	6.924	20
17	6	6	20.30	20
18	6	6	7.174	20
19	6	6	13.12	20

Ordinary one-way ANOVA<br>ANOVA results						
1	Table Analyzed	Bax				
2	Data sets analyzed	A-D				
3						
4	**ANOVA summary**					
5	F	22.79				
6	P value	<0.0001				
7	P value summary	****				
8	Significant diff. among means (P < 0.05)?	Yes				
9	R squared	0.7737				
10						
11	**Brown-Forsythe test**					
12	F (DFn, DFd)	0.1113 (3, 20)				
13	P value	0.9525				
14	P value summary	ns				
15	Are SDs significantly different (P < 0.05)?	No				
16						
17	**Bartlett's test**					
18	Bartlett's statistic (corrected)	1.166				
19	P value	0.7611				
20	P value summary	ns				
21	Are SDs significantly different (P < 0.05)?	No				
22						
23	**ANOVA table**	**SS**	**DF**	**MS**	**F (DFn, DFd)**	**P value**
24	Treatment (between columns)	1.351	3	0.4502	F (3, 20) = 22.79	P<0.0001
25	Residual (within columns)	0.3951	20	0.01975		
26	Total	1.746	23			
27						
28	**Data summary**					
29	Number of treatments (columns)	4				
30	Number of values (total)	24				

Ordinary one-way ANOVA<br>Multiple comparisons					
1	Number of families	1			
2	Number of comparisons per family	6			
3	Alpha	0.05			
4					
5	**Tukey's multiple comparisons t**	**Mean Diff.**	**95.00% CI of diff.**	**Below threshold?**	**Summary**
6	Normal vs. VPCTN	-0.007830	-0.2350 to 0.2193	No	ns
7	Normal vs. DENA	0.5643	0.3372 to 0.7914	Yes	****
8	Normal vs. DENA/VPCTN	0.2999	0.07275 to 0.5270	Yes	**
9	VPCTN vs. DENA	0.5721	0.3450 to 0.7993	Yes	****
10	VPCTN vs. DENA/VPCTN	0.3077	0.08058 to 0.5348	Yes	**
11	DENA vs. DENA/VPCTN	-0.2644	-0.4916 to -0.03730	Yes	*
12					
13	**Test details**	**Mean 1**	**Mean 2**	**Mean Diff.**	**SE of diff.**
14	Normal vs. VPCTN	1.015	1.022	-0.007830	0.08115
15	Normal vs. DENA	1.015	0.4503	0.5643	0.08115
16	Normal vs. DENA/VPCTN	1.015	0.7147	0.2999	0.08115
17	VPCTN vs. DENA	1.022	0.4503	0.5721	0.08115
18	VPCTN vs. DENA/VPCTN	1.022	0.7147	0.3077	0.08115
19	DENA vs. DENA/VPCTN	0.4503	0.7147	-0.2644	0.08115

1				
2				
3				
4				
5	**Adjusted P Value**			
6	0.9997	A-B		
7	<0.0001	A-C		
8	0.0072	A-D		
9	<0.0001	B-C		
10	0.0058	B-D		
11	0.0189	C-D		
12				
13	**n1**	**n2**	**q**	**DF**
14	6	6	0.1365	20
15	6	6	9.835	20
16	6	6	5.226	20
17	6	6	9.971	20
18	6	6	5.363	20
19	6	6	4.608	20

Ordinary one-way ANOVA
ANOVA results

1	Table Analyzed	SOD				
2	Data sets analyzed	A-D				
3						
4	**ANOVA summary**					
5	F	43.87				
6	P value	<0.0001				
7	P value summary	****				
8	Significant diff. among means (P < 0.05)?	Yes				
9	R squared	0.8681				
10						
11	**Brown-Forsythe test**					
12	F (DFn, DFd)	0.8354 (3, 20)				
13	P value	0.4902				
14	P value summary	ns				
15	Are SDs significantly different (P < 0.05)?	No				
16						
17	**Bartlett's test**					
18	Bartlett's statistic (corrected)	2.159				
19	P value	0.5401				
20	P value summary	ns				
21	Are SDs significantly different (P < 0.05)?	No				
22						
23	**ANOVA table**	**SS**	**DF**	**MS**	**F (DFn, DFd)**	**P value**
24	Treatment (between columns)	296.4	3	98.78	F (3, 20) = 43.87	P<0.0001
25	Residual (within columns)	45.03	20	2.252		
26	Total	341.4	23			
27						
28	**Data summary**					
29	Number of treatments (columns)	4				
30	Number of values (total)	24				

Ordinary one-way ANOVA<br>Multiple comparisons					
1	Number of families	1			
2	Number of comparisons per family	6			
3	Alpha	0.05			
4					
5	**Tukey's multiple comparisons test**	**Mean Diff.**	**95.00% CI of diff.**	**Below threshold?**	**Summary**
6	Normal vs. VPCTN	-0.4385	-2.863 to 1.986	No	ns
7	Normal vs. DENA	8.279	5.854 to 10.70	Yes	****
8	Normal vs. DENA/VPCTN	3.854	1.429 to 6.279	Yes	**
9	VPCTN vs. DENA	8.717	6.292 to 11.14	Yes	****
10	VPCTN vs. DENA/VPCTN	4.292	1.868 to 6.717	Yes	***
11	DENA vs. DENA/VPCTN	-4.425	-6.850 to -2.000	Yes	***
12					
13	**Test details**	**Mean 1**	**Mean 2**	**Mean Diff.**	**SE of diff.**
14	Normal vs. VPCTN	11.36	11.80	-0.4385	0.8663
15	Normal vs. DENA	11.36	3.078	8.279	0.8663
16	Normal vs. DENA/VPCTN	11.36	7.503	3.854	0.8663
17	VPCTN vs. DENA	11.80	3.078	8.717	0.8663
18	VPCTN vs. DENA/VPCTN	11.80	7.503	4.292	0.8663
19	DENA vs. DENA/VPCTN	3.078	7.503	-4.425	0.8663

1				
2				
3				
4				
5	**Adjusted P Value**			
6	0.9567	A-B		
7	<0.0001	A-C		
8	0.0013	A-D		
9	<0.0001	B-C		
10	0.0004	B-D		
11	0.0003	C-D		
12				
13	**n1**	**n2**	**q**	**DF**
14	6	6	0.7158	20
15	6	6	13.51	20
16	6	6	6.291	20
17	6	6	14.23	20
18	6	6	7.007	20
19	6	6	7.223	20

Ordinary one-way ANOVA<br>ANOVA results						
1	Table Analyzed	NFkB P65 binding activity				
2	Data sets analyzed	A-D				
3						
4	**ANOVA summary**					
5	F	82.88				
6	P value	<0.0001				
7	P value summary	****				
8	Significant diff. among means (P < 0.05)?	Yes				
9	R squared	0.9256				
10						
11	**Brown-Forsythe test**					
12	F (DFn, DFd)					
13	P value					
14	P value summary					
15	Are SDs significantly different (P < 0.05)?					
16						
17	**Bartlett's test**					
18	Bartlett's statistic (corrected)	21.63				
19	P value	<0.0001				
20	P value summary	****				
21	Are SDs significantly different (P < 0.05)?	Yes				
22						
23	**ANOVA table**	**SS**	**DF**	**MS**	**F (DFn, DFd)**	**P value**
24	Treatment (between columns)	72.82	3	24.27	F (3, 20) = 82.88	P<0.0001
25	Residual (within columns)	5.858	20	0.2929		
26	Total	78.68	23			
27						
28	**Data summary**					
29	Number of treatments (columns)	4				
30	Number of values (total)	24				

**Ordinary one-way ANOVA**<br>Multiple comparisons					
1	Number of families	1			
2	Number of comparisons per family	6			
3	Alpha	0.05			
4					
5	**Tukey's multiple comparisons test**	**Mean Diff.**	**95.00% CI of diff.**	**Below threshold?**	**Summary**
6	Normal vs. VPCTN	0.08900	-0.7855 to 0.9635	No	ns
7	Normal vs. DENA	-4.120	-4.995 to -3.245	Yes	****
8	Normal vs. DENA/VPCTN	-2.217	-3.091 to -1.342	Yes	****
9	VPCTN vs. DENA	-4.209	-5.084 to -3.334	Yes	****
10	VPCTN vs. DENA/VPCTN	-2.306	-3.180 to -1.431	Yes	****
11	DENA vs. DENA/VPCTN	1.904	1.029 to 2.778	Yes	****
12					
13	**Test details**	**Mean 1**	**Mean 2**	**Mean Diff.**	**SE of diff.**
14	Normal vs. VPCTN	1.000	0.9110	0.08900	0.3125
15	Normal vs. DENA	1.000	5.120	-4.120	0.3125
16	Normal vs. DENA/VPCTN	1.000	3.217	-2.217	0.3125
17	VPCTN vs. DENA	0.9110	5.120	-4.209	0.3125
18	VPCTN vs. DENA/VPCTN	0.9110	3.217	-2.306	0.3125
19	DENA vs. DENA/VPCTN	5.120	3.217	1.904	0.3125

1				
2				
3				
4				
5	**Adjusted P Value**			
6	0.9917	A-B		
7	<0.0001	A-C		
8	<0.0001	A-D		
9	<0.0001	B-C		
10	<0.0001	B-D		
11	<0.0001	C-D		
12				
13	**n1**	**n2**	**q**	**DF**
14	6	6	0.4028	20
15	6	6	18.65	20
16	6	6	10.03	20
17	6	6	19.05	20
18	6	6	10.43	20
19	6	6	8.615	20

Ordinary one-way ANOVA
ANOVA results

1	Table Analyzed	STAT3 mRNA				
2	Data sets analyzed	A-D				
3						
4	**ANOVA summary**					
5	F	120.2				
6	P value	<0.0001				
7	P value summary	****				
8	Significant diff. among means (P < 0.05)?	Yes				
9	R squared	0.9474				
10						
11	**Brown-Forsythe test**					
12	F (DFn, DFd)					
13	P value					
14	P value summary					
15	Are SDs significantly different (P < 0.05)?					
16						
17	**Bartlett's test**					
18	Bartlett's statistic (corrected)	19.65				
19	P value	0.0002				
20	P value summary	***				
21	Are SDs significantly different (P < 0.05)?	Yes				
22						
23	**ANOVA table**	**SS**	**DF**	**MS**	**F (DFn, DFd)**	**P value**
24	Treatment (between columns)	114.6	3	38.21	F (3, 20) = 120.2	P<0.0001
25	Residual (within columns)	6.358	20	0.3179		
26	Total	121.0	23			
27						
28	**Data summary**					
29	Number of treatments (columns)	4				
30	Number of values (total)	24				

**Ordinary one-way ANOVA**<br>Multiple comparisons					
1	Number of families	1			
2	Number of comparisons per family	6			
3	Alpha	0.05			
4					
5	**Tukey's multiple comparisons test**	**Mean Diff.**	**95.00% CI of diff.**	**Below threshold?**	**Summary**
6	Normal vs. VPCTN	0.09700	-0.8141 to 1.008	No	ns
7	Normal vs. DENA	-5.170	-6.081 to -4.259	Yes	****
8	Normal vs. DENA/VPCTN	-2.814	-3.725 to -1.903	Yes	****
9	VPCTN vs. DENA	-5.267	-6.178 to -4.356	Yes	****
10	VPCTN vs. DENA/VPCTN	-2.911	-3.822 to -2.000	Yes	****
11	DENA vs. DENA/VPCTN	2.356	1.445 to 3.267	Yes	****
12					
13	**Test details**	**Mean 1**	**Mean 2**	**Mean Diff.**	**SE of diff.**
14	Normal vs. VPCTN	1.000	0.9030	0.09700	0.3255
15	Normal vs. DENA	1.000	6.170	-5.170	0.3255
16	Normal vs. DENA/VPCTN	1.000	3.814	-2.814	0.3255
17	VPCTN vs. DENA	0.9030	6.170	-5.267	0.3255
18	VPCTN vs. DENA/VPCTN	0.9030	3.814	-2.911	0.3255
19	DENA vs. DENA/VPCTN	6.170	3.814	2.356	0.3255

1				
2				
3				
4				
5	**Adjusted P Value**			
6	0.9905	A-B		
7	<0.0001	A-C		
8	<0.0001	A-D		
9	<0.0001	B-C		
10	<0.0001	B-D		
11	<0.0001	C-D		
12				
13	**n1**	**n2**	**q**	**DF**
14	6	6	0.4214	20
15	6	6	22.46	20
16	6	6	12.22	20
17	6	6	22.88	20
18	6	6	12.65	20
19	6	6	10.24	20

Nonlin fit<br>Table of results		A<br>% growth inh.
1	**Comparison of Fits**	
2	Null hypothesis	Yintercept = 0
3	Alternative hypothesis	Yintercept unconstrained
4	P value	<0.0001
5	Conclusion (alpha = 0.05)	Reject null hypothesis
6	Preferred model	Yintercept unconstrained
7	F (DFn, DFd)	138.9 (1, 16)
8		
9	**Yintercept unconstrained**	
10	**Best-fit values**	
11	Yintercept	68.29
12	Slope	-10.30
13	**95% CI (asymptotic)**	
14	Yintercept	56.01 to 80.57
15	Slope	-16.28 to -4.332
16	**Goodness of Fit**	
17	Degrees of Freedom	16
18	R squared	0.4553
19	Sum of Squares	9270
20	Sy.x	24.07
21		
22	**Yintercept = 0**	
23	**Best-fit values**	
24	Yintercept	= 0.000
25	Slope	-3.562
26	**95% CI (asymptotic)**	
27	Slope	-21.13 to 14.01
28	**Goodness of Fit**	
29	Degrees of Freedom	17
30	R squared	-4.274
31	Sum of Squares	89750
32	Sy.x	72.66
33	**Constraints**	
34	Yintercept	Yintercept = 0
35		
36	**Number of points**	
37	# of X values	18
38	# Y values analyzed	18

Nonlin fit<br>Table of results		A<br>% growth inh.
1	**Comparison of Fits**	
2	Null hypothesis	Yintercept = 0
3	Alternative hypothesis	Yintercept unconstrained
4	P value	<0.0001
5	Conclusion (alpha = 0.05)	Reject null hypothesis
6	Preferred model	Yintercept unconstrained
7	F (DFn, DFd)	29.97 (1, 16)
8		
9	**Yintercept unconstrained**	
10	**Best-fit values**	
11	Yintercept	31.68
12	Slope	10.30
13	**95% CI (asymptotic)**	
14	Yintercept	19.41 to 43.95
15	Slope	4.329 to 16.26
16	**Goodness of Fit**	
17	Degrees of Freedom	16
18	R squared	0.4554
19	Sum of Squares	9249
20	Sy.x	24.04
21		
22	**Yintercept = 0**	
23	**Best-fit values**	
24	Yintercept	= 0.000
25	Slope	13.42
26	**95% CI (asymptotic)**	
27	Slope	3.863 to 22.99
28	**Goodness of Fit**	
29	Degrees of Freedom	17
30	R squared	-0.5646
31	Sum of Squares	26573
32	Sy.x	39.54
33	**Constraints**	
34	Yintercept	Yintercept = 0
35		
36	**Number of points**	
37	# of X values	18
38	# Y values analyzed	18

Ordinary one-way ANOVA<br>ANOVA results						
1	Table Analyzed	caspase-1 HepG2 24h				
2	Data sets analyzed	A-C				
3						
4	**ANOVA summary**					
5	F	3.190				
6	P value	0.1139				
7	P value summary	ns				
8	Significant diff. among means (P < 0.05)?	No				
9	R squared	0.5153				
10						
11	**Brown-Forsythe test**					
12	F (DFn, DFd)	0.01102 (2, 6)				
13	P value	0.9891				
14	P value summary	ns				
15	Are SDs significantly different (P < 0.05)?	No				
16						
17	**Bartlett's test**					
18	Bartlett's statistic (corrected)					
19	P value					
20	P value summary					
21	Are SDs significantly different (P < 0.05)?					
22						
23	**ANOVA table**	**SS**	**DF**	**MS**	**F (DFn, DFd)**	**P value**
24	Treatment (between columns)	0.09554	2	0.04777	F (2, 6) = 3.190	P=0.1139
25	Residual (within columns)	0.08986	6	0.01498		
26	Total	0.1854	8			
27						
28	**Data summary**					
29	Number of treatments (columns)	3				
30	Number of values (total)	9				

**Ordinary one-way ANOVA**<br>Multiple comparisons					
1	Number of families	1			
2	Number of comparisons per family	3			
3	Alpha	0.05			
4					
5	**Tukey's multiple comparisons test**	**Mean Diff.**	**95.00% CI of diff.**	**Below threshold?**	**Summary**
6	0 vs. 10	-0.07695	-0.3835 to 0.2296	No	ns
7	0 vs. 20	-0.2466	-0.5532 to 0.05996	No	ns
8	10 vs. 20	-0.1697	-0.4763 to 0.1369	No	ns
9					
10	**Test details**	**Mean 1**	**Mean 2**	**Mean Diff.**	**SE of diff.**
11	0 vs. 10	1.000	1.077	-0.07695	0.09992
12	0 vs. 20	1.000	1.247	-0.2466	0.09992
13	10 vs. 20	1.077	1.247	-0.1697	0.09992

1				
2				
3				
4				
5	**Adjusted P Value**			
6	0.7335	A-B		
7	0.1063	A-C		
8	0.2807	B-C		
9				
10	**n1**	**n2**	**q**	**DF**
11	3	3	1.089	6
12	3	3	3.491	6
13	3	3	2.402	6

Ordinary one-way ANOVA<br>ANOVA results						
1	Table Analyzed	GSH				
2	Data sets analyzed	A-D				
3						
4	**ANOVA summary**					
5	F	109.5				
6	P value	<0.0001				
7	P value summary	****				
8	Significant diff. among means (P < 0.05)?	Yes				
9	R squared	0.9426				
10						
11	**Brown-Forsythe test**					
12	F (DFn, DFd)	1.345 (3, 20)				
13	P value	0.2882				
14	P value summary	ns				
15	Are SDs significantly different (P < 0.05)?	No				
16						
17	**Bartlett's test**					
18	Bartlett's statistic (corrected)	3.377				
19	P value	0.3371				
20	P value summary	ns				
21	Are SDs significantly different (P < 0.05)?	No				
22						
23	**ANOVA table**	**SS**	**DF**	**MS**	**F (DFn, DFd)**	**P value**
24	Treatment (between columns)	535.1	3	178.4	F (3, 20) = 109.5	P<0.0001
25	Residual (within columns)	32.58	20	1.629		
26	Total	567.6	23			
27						
28	**Data summary**					
29	Number of treatments (columns)	4				
30	Number of values (total)	24				

**Ordinary one-way ANOVA** Multiple comparisons					
1	Number of families	1			
2	Number of comparisons per family	6			
3	Alpha	0.05			
4					
5	**Tukey's multiple comparisons test**	**Mean Diff.**	**95.00% CI of diff.**	**Below threshold?**	**Summary**
6	Normal vs. VPCTN	-0.6621	-2.725 to 1.400	No	ns
7	Normal vs. DENA	10.82	8.760 to 12.88	Yes	****
8	Normal vs. DENA/VPCTN	6.222	4.159 to 8.284	Yes	****
9	VPCTN vs. DENA	11.48	9.422 to 13.55	Yes	****
10	VPCTN vs. DENA/VPCTN	6.884	4.821 to 8.946	Yes	****
11	DENA vs. DENA/VPCTN	-4.600	-6.663 to -2.538	Yes	****
12					
13	**Test details**	**Mean 1**	**Mean 2**	**Mean Diff.**	**SE of diff.**
14	Normal vs. VPCTN	18.53	19.19	-0.6621	0.7369
15	Normal vs. DENA	18.53	7.705	10.82	0.7369
16	Normal vs. DENA/VPCTN	18.53	12.31	6.222	0.7369
17	VPCTN vs. DENA	19.19	7.705	11.48	0.7369
18	VPCTN vs. DENA/VPCTN	19.19	12.31	6.884	0.7369
19	DENA vs. DENA/VPCTN	7.705	12.31	-4.600	0.7369

1				
2				
3				
4				
5	**Adjusted P Value**			
6	0.8057	A-B		
7	<0.0001	A-C		
8	<0.0001	A-D		
9	<0.0001	B-C		
10	<0.0001	B-D		
11	<0.0001	C-D		
12				
13	**n1**	**n2**	**q**	**DF**
14	6	6	1.271	20
15	6	6	20.77	20
16	6	6	11.94	20
17	6	6	22.04	20
18	6	6	13.21	20
19	6	6	8.829	20

Ordinary one-way ANOVA<br>ANOVA results						
1	Table Analyzed	MDA				
2	Data sets analyzed	A-D				
3						
4	**ANOVA summary**					
5	F	61.82				
6	P value	<0.0001				
7	P value summary	****				
8	Significant diff. among means (P < 0.05)?	Yes				
9	R squared	0.9027				
10						
11	**Brown-Forsythe test**					
12	F (DFn, DFd)	3.188 (3, 20)				
13	P value	0.0460				
14	P value summary	*				
15	Are SDs significantly different (P < 0.05)?	Yes				
16						
17	**Bartlett's test**					
18	Bartlett's statistic (corrected)	12.14				
19	P value	0.0069				
20	P value summary	**				
21	Are SDs significantly different (P < 0.05)?	Yes				
22						
23	**ANOVA table**	**SS**	**DF**	**MS**	**F (DFn, DFd)**	**P value**
24	Treatment (between columns)	851.5	3	283.8	F (3, 20) = 61.82	P<0.0001
25	Residual (within columns)	91.83	20	4.591		
26	Total	943.3	23			
27						
28	**Data summary**					
29	Number of treatments (columns)	4				
30	Number of values (total)	24				

**Ordinary one-way ANOVA**<br>Multiple comparisons					
1	Number of families	1			
2	Number of comparisons per family	6			
3	Alpha	0.05			
4					
5	**Tukey's multiple comparisons test**	**Mean Diff.**	**95.00% CI of diff.**	**Below threshold?**	**Summary**
6	Normal vs. VPCTN	0.2246	-3.238 to 3.687	No	ns
7	Normal vs. DENA	-14.43	-17.89 to -10.96	Yes	****
8	Normal vs. DENA/VPCTN	-5.880	-9.342 to -2.417	Yes	***
9	VPCTN vs. DENA	-14.65	-18.11 to -11.19	Yes	****
10	VPCTN vs. DENA/VPCTN	-6.104	-9.567 to -2.642	Yes	***
11	DENA vs. DENA/VPCTN	8.546	5.084 to 12.01	Yes	****
12					
13	**Test details**	**Mean 1**	**Mean 2**	**Mean Diff.**	**SE of diff.**
14	Normal vs. VPCTN	11.97	11.74	0.2246	1.237
15	Normal vs. DENA	11.97	26.39	-14.43	1.237
16	Normal vs. DENA/VPCTN	11.97	17.85	-5.880	1.237
17	VPCTN vs. DENA	11.74	26.39	-14.65	1.237
18	VPCTN vs. DENA/VPCTN	11.74	17.85	-6.104	1.237
19	DENA vs. DENA/VPCTN	26.39	17.85	8.546	1.237

1				
2				
3				
4				
5	**Adjusted P Value**			
6	0.9978	A-B		
7	<0.0001	A-C		
8	0.0006	A-D		
9	<0.0001	B-C		
10	0.0004	B-D		
11	<0.0001	C-D		
12				
13	**n1**	**n2**	**q**	**DF**
14	6	6	0.2568	20
15	6	6	16.49	20
16	6	6	6.722	20
17	6	6	16.75	20
18	6	6	6.978	20
19	6	6	9.770	20

Ordinary one-way ANOVA<br>ANOVA results						
1	Table Analyzed	MCP-1				
2	Data sets analyzed	A-D				
3						
4	**ANOVA summary**					
5	F	30.99				
6	P value	<0.0001				
7	P value summary	****				
8	Significant diff. among means (P < 0.05)?	Yes				
9	R squared	0.8229				
10						
11	**Brown-Forsythe test**					
12	F (DFn, DFd)	1.812 (3, 20)				
13	P value	0.1774				
14	P value summary	ns				
15	Are SDs significantly different (P < 0.05)?	No				
16						
17	**Bartlett's test**					
18	Bartlett's statistic (corrected)	17.68				
19	P value	0.0005				
20	P value summary	***				
21	Are SDs significantly different (P < 0.05)?	Yes				
22						
23	**ANOVA table**	**SS**	**DF**	**MS**	**F (DFn, DFd)**	**P value**
24	Treatment (between columns)	32.50	3	10.83	F (3, 20) = 30.99	P<0.0001
25	Residual (within columns)	6.993	20	0.3496		
26	Total	39.49	23			
27						
28	**Data summary**					
29	Number of treatments (columns)	4				
30	Number of values (total)	24				

Ordinary one-way ANOVA<br>Multiple comparisons					
1	Number of families	1			
2	Number of comparisons per family	6			
3	Alpha	0.05			
4					
5	**Tukey's multiple comparisons test**	**Mean Diff.**	**95.00% CI of diff.**	**Below threshold?**	**Summary**
6	Normal vs. VPCTN	-0.01622	-0.9717 to 0.9393	No	ns
7	Normal vs. DENA	-2.759	-3.714 to -1.803	Yes	****
8	Normal vs. DENA/VPCTN	-1.630	-2.586 to -0.6747	Yes	***
9	VPCTN vs. DENA	-2.742	-3.698 to -1.787	Yes	****
10	VPCTN vs. DENA/VPCTN	-1.614	-2.570 to -0.6585	Yes	***
11	DENA vs. DENA/VPCTN	1.128	0.1729 to 2.084	Yes	*
12					
13	**Test details**	**Mean 1**	**Mean 2**	**Mean Diff.**	**SE of diff.**
14	Normal vs. VPCTN	2.136	2.152	-0.01622	0.3414
15	Normal vs. DENA	2.136	4.894	-2.759	0.3414
16	Normal vs. DENA/VPCTN	2.136	3.766	-1.630	0.3414
17	VPCTN vs. DENA	2.152	4.894	-2.742	0.3414
18	VPCTN vs. DENA/VPCTN	2.152	3.766	-1.614	0.3414
19	DENA vs. DENA/VPCTN	4.894	3.766	1.128	0.3414

1				
2				
3				
4				
5	**Adjusted P Value**			
6	>0.9999	A-B		
7	<0.0001	A-C		
8	0.0006	A-D		
9	<0.0001	B-C		
10	0.0007	B-D		
11	0.0171	C-D		
12				
13	**n1**	**n2**	**q**	**DF**
14	6	6	0.06718	20
15	6	6	11.43	20
16	6	6	6.753	20
17	6	6	11.36	20
18	6	6	6.686	20
19	6	6	4.675	20

Ordinary one-way ANOVA<br>ANOVA results						
1	Table Analyzed	ICAM-1				
2	Data sets analyzed	A-D				
3						
4	**ANOVA summary**					
5	F	131.7				
6	P value	<0.0001				
7	P value summary	****				
8	Significant diff. among means (P < 0.05)?	Yes				
9	R squared	0.9518				
10						
11	**Brown-Forsythe test**					
12	F (DFn, DFd)	6.004 (3, 20)				
13	P value	0.0043				
14	P value summary	**				
15	Are SDs significantly different (P < 0.05)?	Yes				
16						
17	**Bartlett's test**					
18	Bartlett's statistic (corrected)	27.79				
19	P value	<0.0001				
20	P value summary	****				
21	Are SDs significantly different (P < 0.05)?	Yes				
22						
23	**ANOVA table**	**SS**	**DF**	**MS**	**F (DFn, DFd)**	**P value**
24	Treatment (between columns)	3519	3	1173	F (3, 20) = 131.7	P<0.0001
25	Residual (within columns)	178.1	20	8.906		
26	Total	3697	23			
27						
28	**Data summary**					
29	Number of treatments (columns)	4				
30	Number of values (total)	24				

**Ordinary one-way ANOVA**<br>Multiple comparisons					
1	Number of families	1			
2	Number of comparisons per family	6			
3	Alpha	0.05			
4					
5	**Tukey's multiple comparisons test**	**Mean Diff.**	**95.00% CI of diff.**	**Below threshold?**	**Summary**
6	Normal vs. VPCTN	-0.08848	-4.911 to 4.734	No	ns
7	Normal vs. DENA	-29.70	-34.53 to -24.88	Yes	****
8	Normal vs. DENA/VPCTN	-10.18	-15.00 to -5.356	Yes	****
9	VPCTN vs. DENA	-29.62	-34.44 to -24.79	Yes	****
10	VPCTN vs. DENA/VPCTN	-10.09	-14.91 to -5.267	Yes	****
11	DENA vs. DENA/VPCTN	19.53	14.70 to 24.35	Yes	****
12					
13	**Test details**	**Mean 1**	**Mean 2**	**Mean Diff.**	**SE of diff.**
14	Normal vs. VPCTN	5.006	5.094	-0.08848	1.723
15	Normal vs. DENA	5.006	34.71	-29.70	1.723
16	Normal vs. DENA/VPCTN	5.006	15.18	-10.18	1.723
17	VPCTN vs. DENA	5.094	34.71	-29.62	1.723
18	VPCTN vs. DENA/VPCTN	5.094	15.18	-10.09	1.723
19	DENA vs. DENA/VPCTN	34.71	15.18	19.53	1.723

1				
2				
3				
4				
5	**Adjusted P Value**			
6	>0.9999	A-B		
7	<0.0001	A-C		
8	<0.0001	A-D		
9	<0.0001	B-C		
10	<0.0001	B-D		
11	<0.0001	C-D		
12				
13	**n1**	**n2**	**q**	**DF**
14	6	6	0.07263	20
15	6	6	24.38	20
16	6	6	8.354	20
17	6	6	24.31	20
18	6	6	8.282	20
19	6	6	16.03	20

Ordinary one-way ANOVA<br>ANOVA results						
1	Table Analyzed	HIF-1 alpha				
2	Data sets analyzed	A-D				
3						
4	**ANOVA summary**					
5	F	218.7				
6	P value	<0.0001				
7	P value summary	****				
8	Significant diff. among means (P < 0.05)?	Yes				
9	R squared	0.9704				
10						
11	**Brown-Forsythe test**					
12	F (DFn, DFd)	8.717 (3, 20)				
13	P value	0.0007				
14	P value summary	***				
15	Are SDs significantly different (P < 0.05)?	Yes				
16						
17	**Bartlett's test**					
18	Bartlett's statistic (corrected)	55.18				
19	P value	<0.0001				
20	P value summary	****				
21	Are SDs significantly different (P < 0.05)?	Yes				
22						
23	**ANOVA table**	**SS**	**DF**	**MS**	**F (DFn, DFd)**	**P value**
24	Treatment (between columns)	1873551	3	624517	F (3, 20) = 218.7	P<0.0001
25	Residual (within columns)	57108	20	2855		
26	Total	1930659	23			
27						
28	**Data summary**					
29	Number of treatments (columns)	4				
30	Number of values (total)	24				

**Ordinary one-way ANOVA**<br>Multiple comparisons					
1	Number of families	1			
2	Number of comparisons per family	6			
3	Alpha	0.05			
4					
5	**Tukey's multiple comparisons test**	**Mean Diff.**	**95.00% CI of diff.**	**Below threshold?**	**Summary**
6	Normal vs. VPCTN	4.262	-82.09 to 90.61	No	ns
7	Normal vs. DENA	-673.7	-760.1 to -587.4	Yes	****
8	Normal vs. DENA/VPCTN	-121.5	-207.9 to -35.16	Yes	**
9	VPCTN vs. DENA	-678.0	-764.3 to -591.6	Yes	****
10	VPCTN vs. DENA/VPCTN	-125.8	-212.1 to -39.42	Yes	**
11	DENA vs. DENA/VPCTN	552.2	465.8 to 638.5	Yes	****
12					
13	**Test details**	**Mean 1**	**Mean 2**	**Mean Diff.**	**SE of diff.**
14	Normal vs. VPCTN	35.84	31.58	4.262	30.85
15	Normal vs. DENA	35.84	709.5	-673.7	30.85
16	Normal vs. DENA/VPCTN	35.84	157.4	-121.5	30.85
17	VPCTN vs. DENA	31.58	709.5	-678.0	30.85
18	VPCTN vs. DENA/VPCTN	31.58	157.4	-125.8	30.85
19	DENA vs. DENA/VPCTN	709.5	157.4	552.2	30.85

1				
2				
3				
4				
5	**Adjusted P Value**			
6	0.9990	A-B		
7	<0.0001	A-C		
8	0.0042	A-D		
9	<0.0001	B-C		
10	0.0030	B-D		
11	<0.0001	C-D		
12				
13	**n1**	**n2**	**q**	**DF**
14	6	6	0.1953	20
15	6	6	30.88	20
16	6	6	5.570	20
17	6	6	31.08	20
18	6	6	5.765	20
19	6	6	25.31	20

Ordinary one-way ANOVA<br>ANOVA results						
1	Table Analyzed	p-STAT3 normalized abs				
2	Data sets analyzed	A-D				
3						
4	**ANOVA summary**					
5	F	17.50				
6	P value	<0.0001				
7	P value summary	****				
8	Significant diff. among means (P < 0.05)?	Yes				
9	R squared	0.7241				
10						
11	**Brown-Forsythe test**					
12	F (DFn, DFd)	1.932 (3, 20)				
13	P value	0.1569				
14	P value summary	ns				
15	Are SDs significantly different (P < 0.05)?	No				
16						
17	**Bartlett's test**					
18	Bartlett's statistic (corrected)	17.57				
19	P value	0.0005				
20	P value summary	***				
21	Are SDs significantly different (P < 0.05)?	Yes				
22						
23	**ANOVA table**	**SS**	**DF**	**MS**	**F (DFn, DFd)**	**P value**
24	Treatment (between columns)	7.197	3	2.399	F (3, 20) = 17.50	P<0.0001
25	Residual (within columns)	2.742	20	0.1371		
26	Total	9.940	23			
27						
28	**Data summary**					
29	Number of treatments (columns)	4				
30	Number of values (total)	24				

**Ordinary one-way ANOVA**<br>Multiple comparisons					
1	Number of families	1			
2	Number of comparisons per family	6			
3	Alpha	0.05			
4					
5	**Tukey's multiple comparisons test**	**Mean Diff.**	**95.00% CI of diff.**	**Below threshold?**	**Summary**
6	Normal vs. VPCTN	0.08652	-0.5118 to 0.6849	No	ns
7	Normal vs. DENA	-1.294	-1.892 to -0.6953	Yes	****
8	Normal vs. DENA/VPCTN	-0.4780	-1.076 to 0.1204	No	ns
9	VPCTN vs. DENA	-1.380	-1.979 to -0.7818	Yes	****
10	VPCTN vs. DENA/VPCTN	-0.5645	-1.163 to 0.03384	No	ns
11	DENA vs. DENA/VPCTN	0.8156	0.2173 to 1.414	Yes	**
12					
13	**Test details**	**Mean 1**	**Mean 2**	**Mean Diff.**	**SE of diff.**
14	Normal vs. VPCTN	1.000	0.9135	0.08652	0.2138
15	Normal vs. DENA	1.000	2.294	-1.294	0.2138
16	Normal vs. DENA/VPCTN	1.000	1.478	-0.4780	0.2138
17	VPCTN vs. DENA	0.9135	2.294	-1.380	0.2138
18	VPCTN vs. DENA/VPCTN	0.9135	1.478	-0.5645	0.2138
19	DENA vs. DENA/VPCTN	2.294	1.478	0.8156	0.2138

1				
2				
3				
4				
5	**Adjusted P Value**			
6	0.9770	A-B		
7	<0.0001	A-C		
8	0.1477	A-D		
9	<0.0001	B-C		
10	0.0688	B-D		
11	0.0055	C-D		
12				
13	**n1**	**n2**	**q**	**DF**
14	6	6	0.5723	20
15	6	6	8.558	20
16	6	6	3.162	20
17	6	6	9.130	20
18	6	6	3.734	20
19	6	6	5.395	20

**Ordinary one-way ANOVA**<br>ANOVA results					
1	Table Analyzed	Capase-3 IHC			
2	Data sets analyzed	A-D			
3					
4	**ANOVA summary**				
5	F	37.69			
6	P value	<0.0001			
7	P value summary	****			
8	Significant diff. among means (P < 0.05)?	Yes			
9	R squared	0.8497			
10					
11	**Brown-Forsythe test**				
12	F (DFn, DFd)	1.288 (3, 20)			
13	P value	0.3057			
14	P value summary	ns			
15	Are SDs significantly different (P < 0.05)?	No			
16					
17	**Bartlett's test**				
18	Bartlett's statistic (corrected)	6.094			
19	P value	0.1071			
20	P value summary	ns			
21	Are SDs significantly different (P < 0.05)?	No			
22					
23	**ANOVA table**	**SS**	**DF**	**MS**	**F (DFn, DFd)**
24	Treatment (between columns)	121.7	3	40.58	F (3, 20) = 37.69
25	Residual (within columns)	21.54	20	1.077	
26	Total	143.3	23		
27					
28	**Data summary**				
29	Number of treatments (columns)	4			
30	Number of values (total)	24			

1	
2	
3	
4	
5	
6	
7	
8	
9	
10	
11	
12	
13	
14	
15	
16	
17	
18	
19	
20	
21	
22	
23	**P value**
24	P<0.0001
25	
26	
27	
28	
29	
30	

Ordinary one-way ANOVA<br>Multiple comparisons					
1	Number of families	1			
2	Number of comparisons per family	6			
3	Alpha	0.05			
4					
5	**Tukey's multiple comparisons test**	**Mean Diff.**	**95.00% CI of diff.**	**Below threshold?**	**Summary**
6	Normal vs. VPCTN	-0.2667	-1.944 to 1.410	No	ns
7	Normal vs. DENA	1.450	-0.2269 to 3.127	No	ns
8	Normal vs. DENA/VPCTN	-4.583	-6.260 to -2.906	Yes	****
9	VPCTN vs. DENA	1.717	0.03977 to 3.394	Yes	*
10	VPCTN vs. DENA/VPCTN	-4.317	-5.994 to -2.640	Yes	****
11	DENA vs. DENA/VPCTN	-6.033	-7.710 to -4.356	Yes	****
12					
13	**Test details**	**Mean 1**	**Mean 2**	**Mean Diff.**	**SE of diff.**
14	Normal vs. VPCTN	4.100	4.367	-0.2667	0.5991
15	Normal vs. DENA	4.100	2.650	1.450	0.5991
16	Normal vs. DENA/VPCTN	4.100	8.683	-4.583	0.5991
17	VPCTN vs. DENA	4.367	2.650	1.717	0.5991
18	VPCTN vs. DENA/VPCTN	4.367	8.683	-4.317	0.5991
19	DENA vs. DENA/VPCTN	2.650	8.683	-6.033	0.5991

1				
2				
3				
4				
5	**Adjusted P Value**			
6	0.9698	A-B		
7	0.1053	A-C		
8	<0.0001	A-D		
9	0.0436	B-C		
10	<0.0001	B-D		
11	<0.0001	C-D		
12				
13	**n1**	**n2**	**q**	**DF**
14	6	6	0.6295	20
15	6	6	3.423	20
16	6	6	10.82	20
17	6	6	4.052	20
18	6	6	10.19	20
19	6	6	14.24	20

**Ordinary one-way ANOVA**<br>ANOVA results					
1	Table Analyzed	Ki-67 IHC			
2	Data sets analyzed	A-D			
3					
4	**ANOVA summary**				
5	F	47.83			
6	P value	<0.0001			
7	P value summary	****			
8	Significant diff. among means (P < 0.05)?	Yes			
9	R squared	0.8777			
10					
11	**Brown-Forsythe test**				
12	F (DFn, DFd)	5.407 (3, 20)			
13	P value	0.0069			
14	P value summary	**			
15	Are SDs significantly different (P < 0.05)?	Yes			
16					
17	**Bartlett's test**				
18	Bartlett's statistic (corrected)	12.44			
19	P value	0.0060			
20	P value summary	**			
21	Are SDs significantly different (P < 0.05)?	Yes			
22					
23	**ANOVA table**	**SS**	**DF**	**MS**	**F (DFn, DFd)**
24	Treatment (between columns)	262.8	3	87.59	F (3, 20) = 47.83
25	Residual (within columns)	36.62	20	1.831	
26	Total	299.4	23		
27					
28	**Data summary**				
29	Number of treatments (columns)	4			
30	Number of values (total)	24			

1	
2	
3	
4	
5	
6	
7	
8	
9	
10	
11	
12	
13	
14	
15	
16	
17	
18	
19	
20	
21	
22	
23	**P value**
24	P<0.0001
25	
26	
27	
28	
29	
30	

Ordinary one-way ANOVA<br>Multiple comparisons					
1	Number of families	1			
2	Number of comparisons per family	6			
3	Alpha	0.05			
4					
5	**Tukey's multiple comparisons test**	**Mean Diff.**	**95.00% CI of diff.**	**Below threshold?**	**Summary**
6	Normal vs. VPCTN	0.1850	-2.002 to 2.372	No	ns
7	Normal vs. DENA	-8.000	-10.19 to -5.813	Yes	****
8	Normal vs. DENA/VPCTN	-3.000	-5.187 to -0.8133	Yes	**
9	VPCTN vs. DENA	-8.185	-10.37 to -5.998	Yes	****
10	VPCTN vs. DENA/VPCTN	-3.185	-5.372 to -0.9983	Yes	**
11	DENA vs. DENA/VPCTN	5.000	2.813 to 7.187	Yes	****
12					
13	**Test details**	**Mean 1**	**Mean 2**	**Mean Diff.**	**SE of diff.**
14	Normal vs. VPCTN	2.183	1.998	0.1850	0.7813
15	Normal vs. DENA	2.183	10.18	-8.000	0.7813
16	Normal vs. DENA/VPCTN	2.183	5.183	-3.000	0.7813
17	VPCTN vs. DENA	1.998	10.18	-8.185	0.7813
18	VPCTN vs. DENA/VPCTN	1.998	5.183	-3.185	0.7813
19	DENA vs. DENA/VPCTN	10.18	5.183	5.000	0.7813

1				
2				
3				
4				
5	**Adjusted P Value**			
6	0.9952	A-B		
7	<0.0001	A-C		
8	0.0052	A-D		
9	<0.0001	B-C		
10	0.0030	B-D		
11	<0.0001	C-D		
12				
13	**n1**	**n2**	**q**	**DF**
14	6	6	0.3349	20
15	6	6	14.48	20
16	6	6	5.430	20
17	6	6	14.82	20
18	6	6	5.765	20
19	6	6	9.051	20

Ordinary one-way ANOVA
ANOVA results

1	Table Analyzed	fibrosis SR			
2	Data sets analyzed	A-D			
3					
4	**ANOVA summary**				
5	F	10.68			
6	P value	0.0002			
7	P value summary	***			
8	Significant diff. among means (P < 0.05)?	Yes			
9	R squared	0.6157			
10					
11	**Brown-Forsythe test**				
12	F (DFn, DFd)	1.584 (3, 20)			
13	P value	0.2245			
14	P value summary	ns			
15	Are SDs significantly different (P < 0.05)?	No			
16					
17	**Bartlett's test**				
18	Bartlett's statistic (corrected)	2.576			
19	P value	0.4617			
20	P value summary	ns			
21	Are SDs significantly different (P < 0.05)?	No			
22					
23	**ANOVA table**	**SS**	**DF**	**MS**	**F (DFn, DFd)**
24	Treatment (between columns)	48.77	3	16.26	F (3, 20) = 10.68
25	Residual (within columns)	30.44	20	1.522	
26	Total	79.20	23		
27					
28	**Data summary**				
29	Number of treatments (columns)	4			
30	Number of values (total)	24			

1	
2	
3	
4	
5	
6	
7	
8	
9	
10	
11	
12	
13	
14	
15	
16	
17	
18	
19	
20	
21	
22	
23	**P value**
24	P=0.0002
25	
26	
27	
28	
29	
30	

**Ordinary one-way ANOVA**<br>Multiple comparisons					
1	Number of families	1			
2	Number of comparisons per family	6			
3	Alpha	0.05			
4					
5	**Tukey's multiple comparisons test**	**Mean Diff.**	**95.00% CI of diff.**	**Below threshold?**	**Summary**
6	Normal vs. VPCTN	-0.1646	-2.158 to 1.829	No	ns
7	Normal vs. DENA	-3.569	-5.563 to -1.576	Yes	***
8	Normal vs. DENA/VPCTN	-1.346	-3.340 to 0.6474	No	ns
9	VPCTN vs. DENA	-3.405	-5.398 to -1.411	Yes	***
10	VPCTN vs. DENA/VPCTN	-1.182	-3.175 to 0.8120	No	ns
11	DENA vs. DENA/VPCTN	2.223	0.2298 to 4.217	Yes	*
12					
13	**Test details**	**Mean 1**	**Mean 2**	**Mean Diff.**	**SE of diff.**
14	Normal vs. VPCTN	4.491	4.656	-0.1646	0.7122
15	Normal vs. DENA	4.491	8.060	-3.569	0.7122
16	Normal vs. DENA/VPCTN	4.491	5.837	-1.346	0.7122
17	VPCTN vs. DENA	4.656	8.060	-3.405	0.7122
18	VPCTN vs. DENA/VPCTN	4.656	5.837	-1.182	0.7122
19	DENA vs. DENA/VPCTN	8.060	5.837	2.223	0.7122

1				
2				
3				
4				
5	**Adjusted P Value**			
6	0.9955	A-B		
7	0.0004	A-C		
8	0.2637	A-D		
9	0.0006	B-C		
10	0.3703	B-D		
11	0.0254	C-D		
12				
13	**n1**	**n2**	**q**	**DF**
14	6	6	0.3268	20
15	6	6	7.087	20
16	6	6	2.673	20
17	6	6	6.761	20
18	6	6	2.346	20
19	6	6	4.415	20

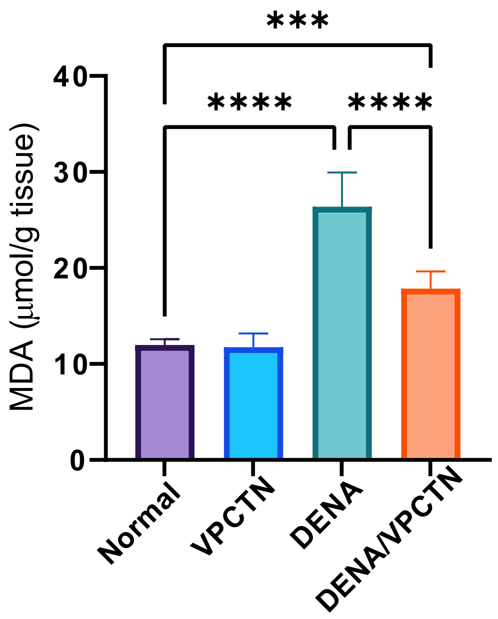

***
****
****
MDA (µmol/g tissue)
40
30
20
10
0
Normal
VPCTN
DENA
DENA/VPCTN

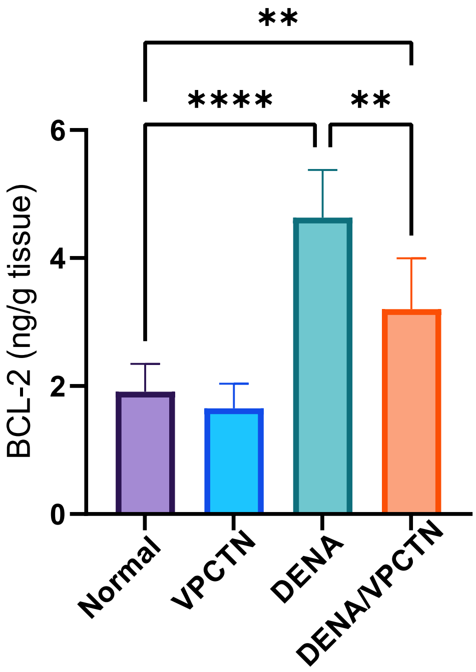

**
****
**
BCL-2 (ng/g tissue)
0
2
4
6
Normal
VPCTN
DENA
DENA/VPCTN

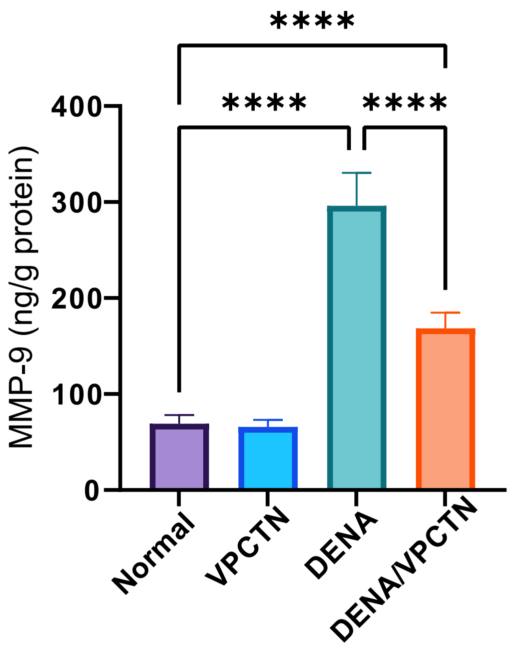

****
****
****
400
300
200
100
0
MMP-9 (ng/g protein)
Normal
VPCTN
DENA
DENA/VPCTN

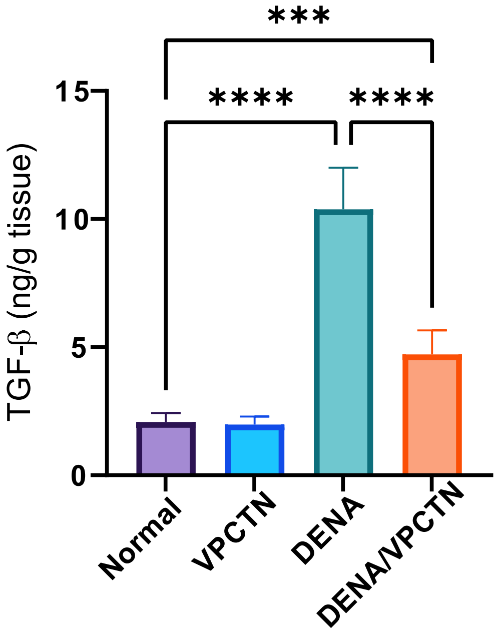

***
****
****
15
10
5
0
TGF-β (ng/g tissue)
Normal
VPCTN
DENA
DENA/VPCTN

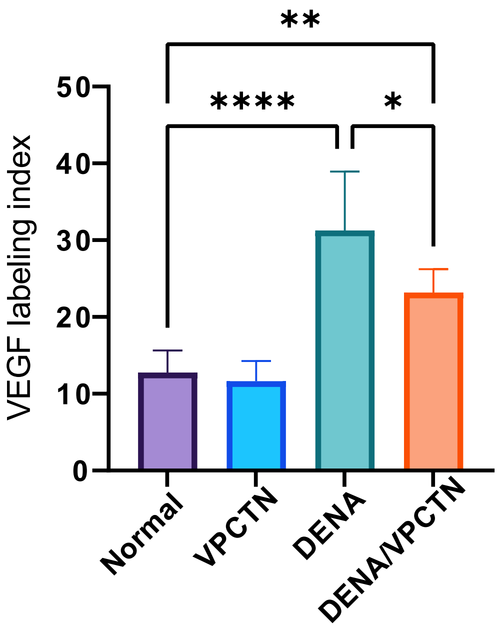

**
****
*
VEGF labeling index
50
40
30
20
10
0
Normal
VPCTN
DENA
DENA/VPCTN

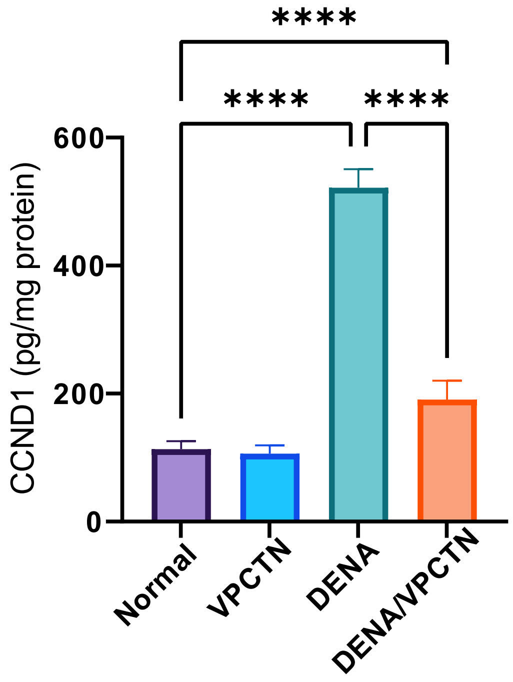

****
****
****
600
400
200
0
CCND1 (pg/mg protein)
Normal
VPCTN
DENA
DENA/VPCTN

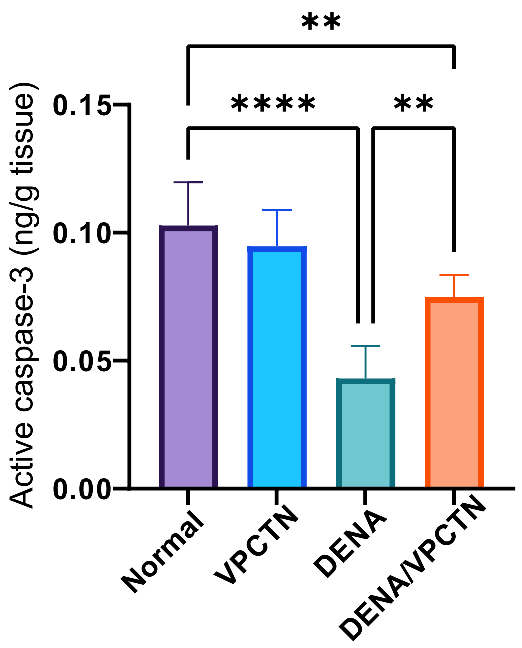

**
****
**
0.15
0.10
0.05
0.00
Active caspase-3 (ng/g tissue)
Normal
VPCTN
DENA
DENA/VPCTN

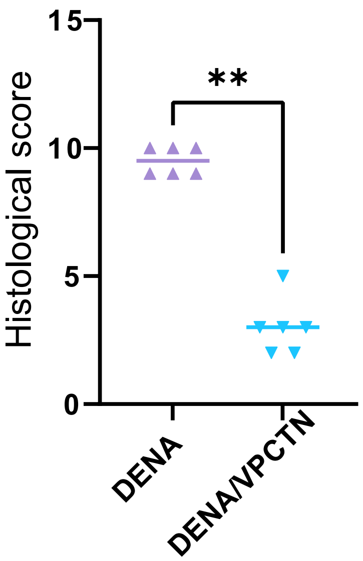
Histological score
15
10
5
0
**
DENA
DENA/VPCTN

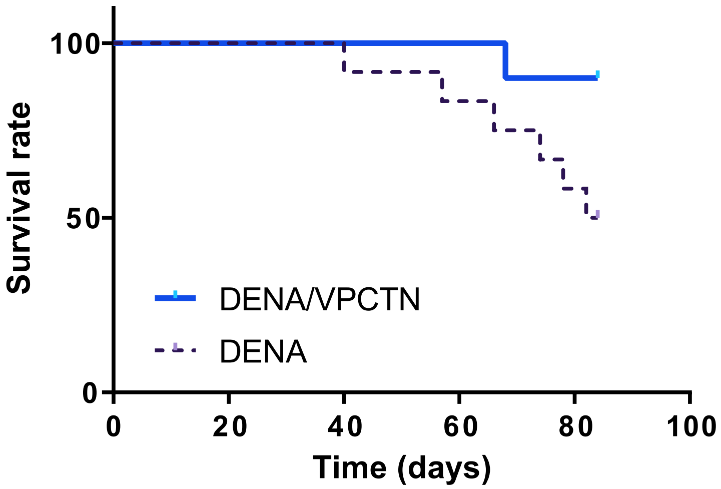

Hazard Ratio (logrank) = 6.2
P = 0.05
95% CI of ratio 1.402 to 27.15

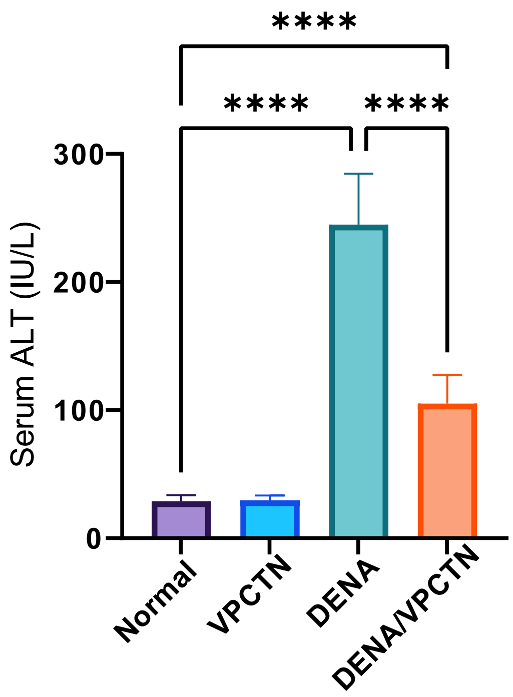
****
****
****
300
200
100
0
Serum ALT (IU/L)
Normal
VPCTN
DENA
DENA/VPCTN

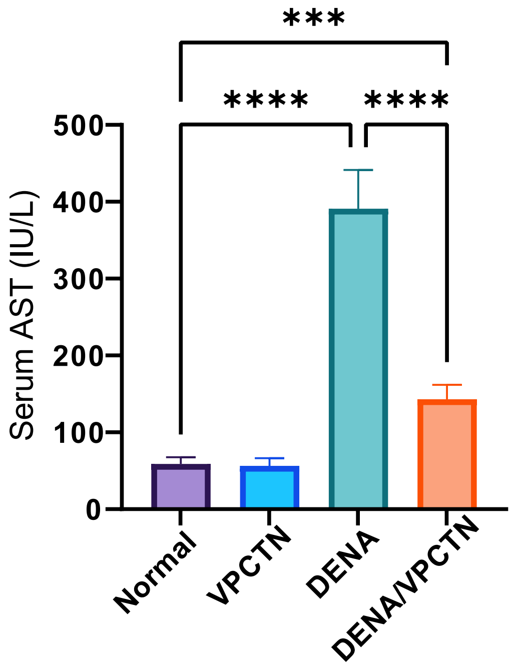
***
****
****
500
400
300
200
100
0
Serum AST (IU/L)
Normal
VPCTN
DENA
DENA/VPCTN

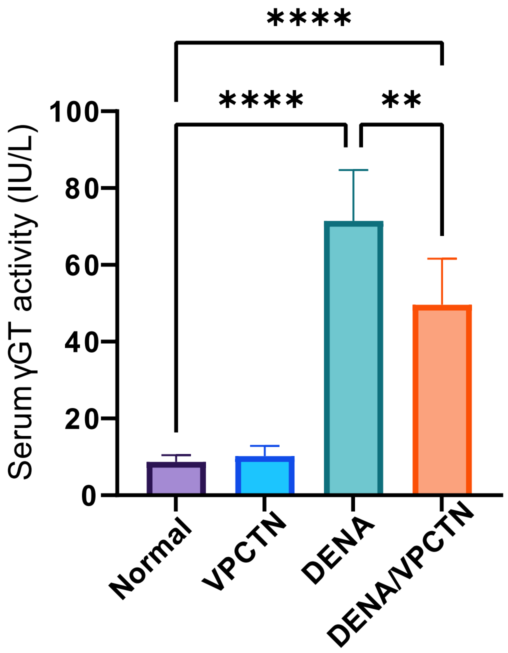

****
****
**
100
80
60
40
20
0
Serum γGT activity (IU/L)
Normal
VPCTN
DENA
DENA/VPCTN

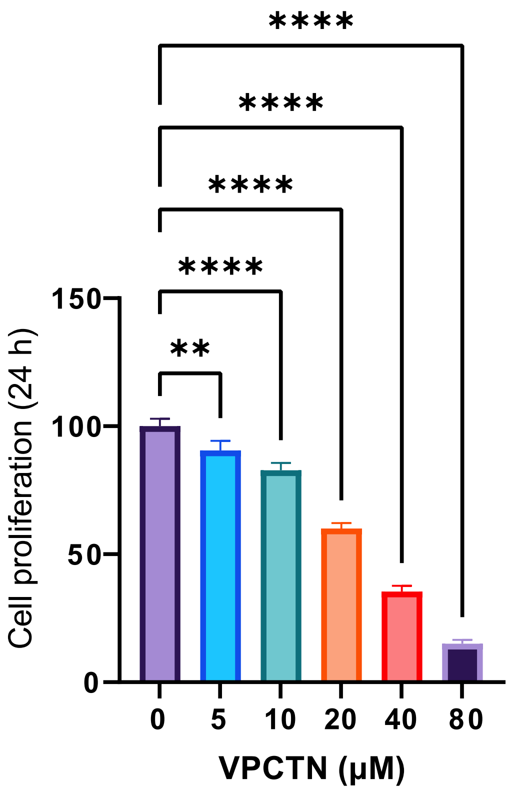

****
****
****
****
**
150
100
50
0
Cell proliferation (24 h)
0
5
10
20
40
80
VPCTN (μM)

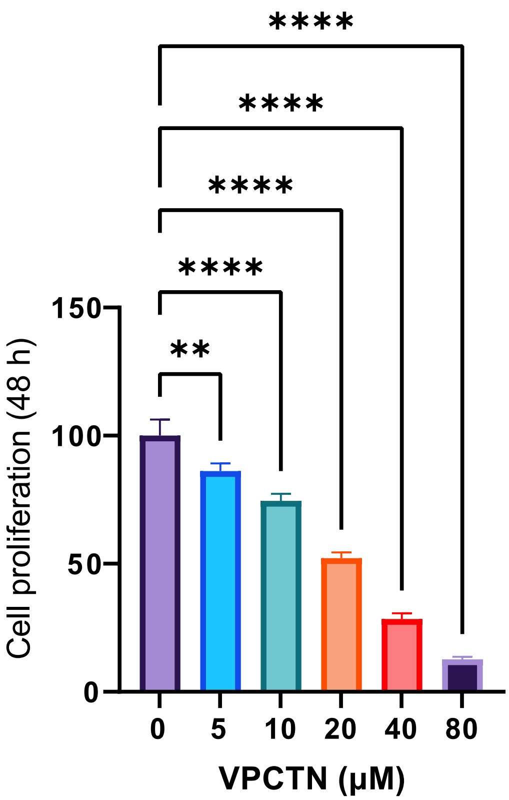
****
****
****
****
**
150
100
50
0
Cell proliferation (48 h)
0
5
10
20
40
80
VPCTN (μM)

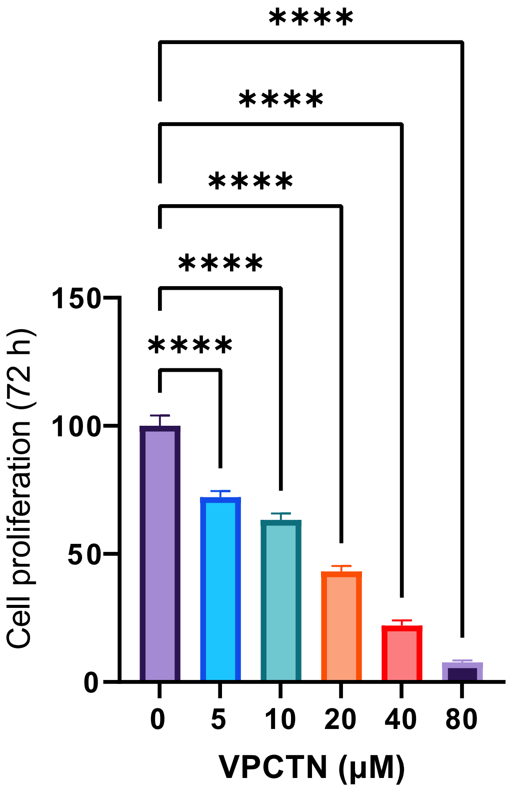

****
****
****
****
****
150
100
50
0
Cell proliferation (72 h)
0
5
10
20
40
80
VPCTN (μM)

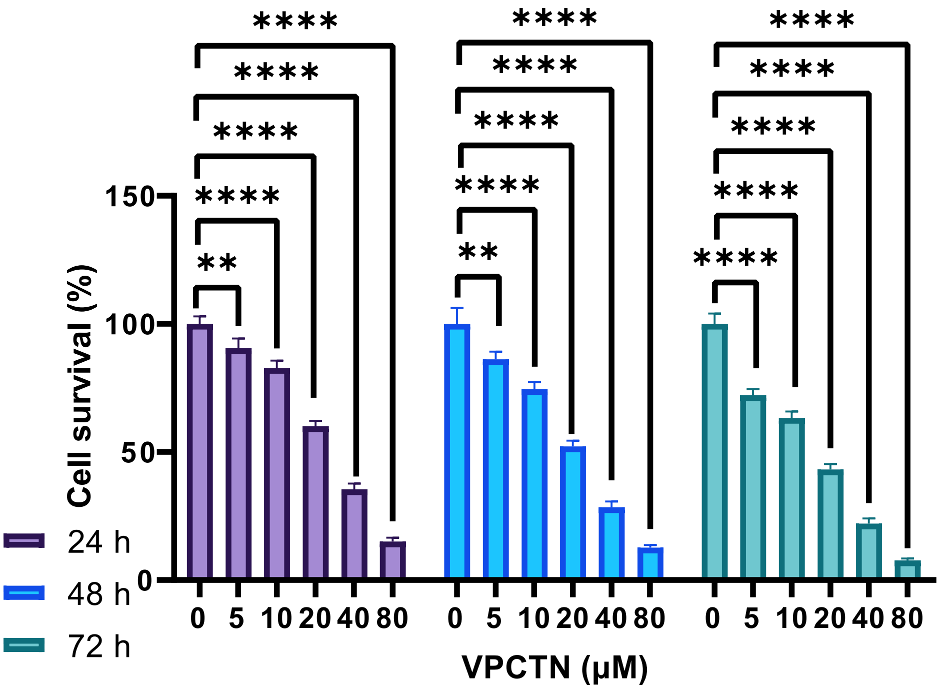

****
****
****
****
**
****
****
****
****
**
****
****
****
****
****
150
100
50
0
Cell survival (%)
24 h
48 h
72 h
0
5
10
20
40
80
0
5
10
20
40
80
0
5
10
20
40
80
VPCTN (µM)

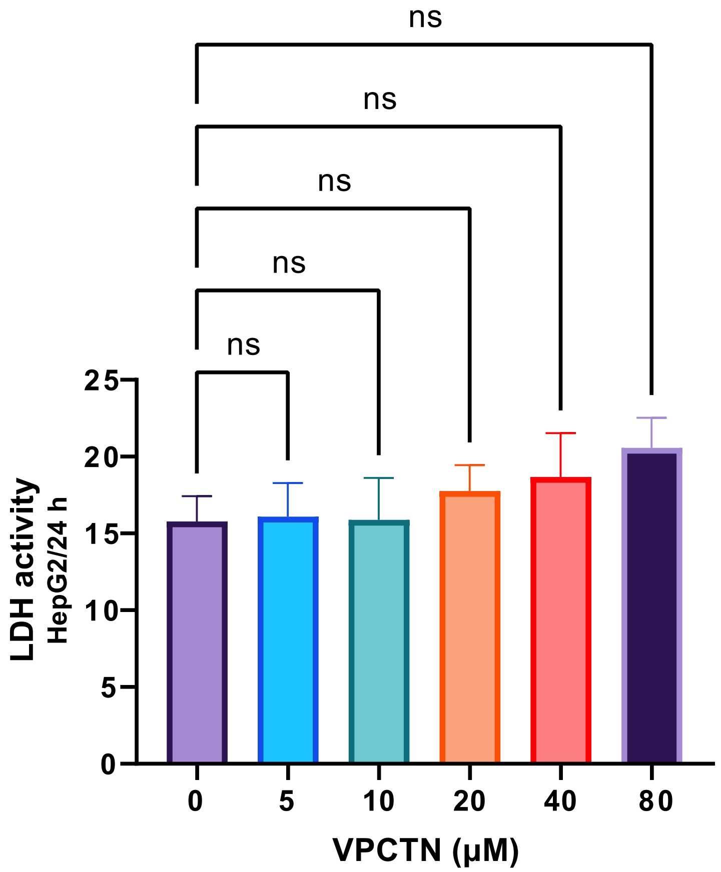

ns
ns
ns
ns
ns
25
20
15
10
5
0
LDH activity
HepG2/24 h
0
5
10
20
40
80
VPCTN (μM)

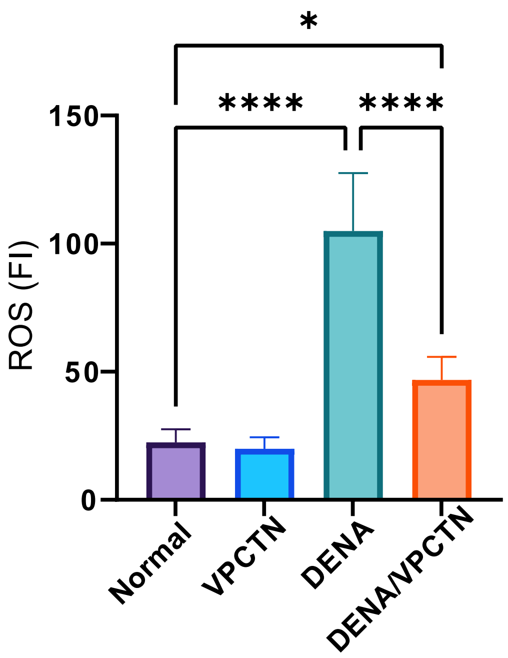

*
****
****
150
100
50
0
ROS (FI)
Normal
VPCTN
DENA
DENA/VPCTN

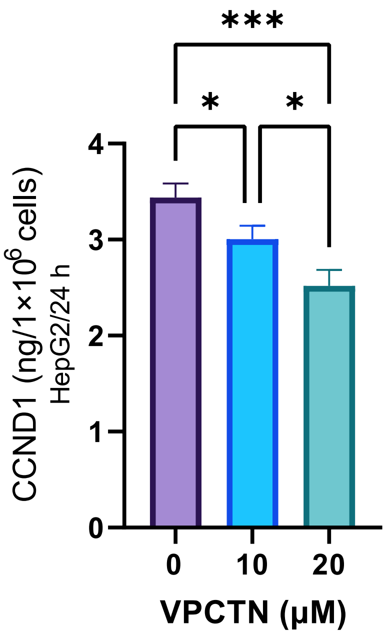

***
*
*
4
3
2
1
0
CCND1 (ng/1×10^6 cells)
HepG2/24 h
0
10
20
VPCTN (μM)

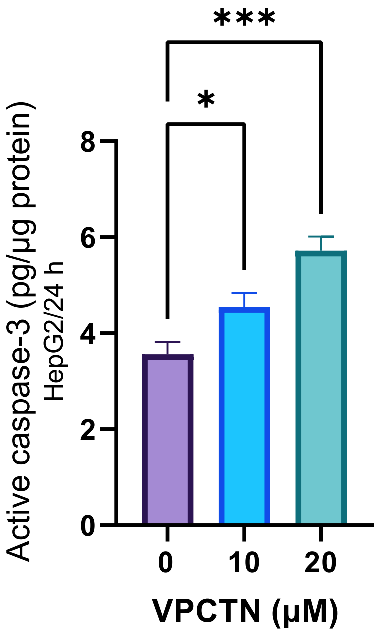

***
*
Active caspase-3 (pg/µg protein)
HepG2/24 h
8
6
4
2
0
0
10
20
VPCTN (µM)

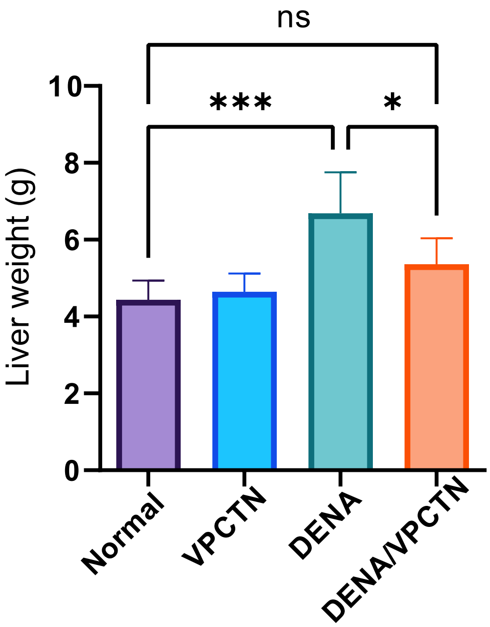
ns
***
*
10
8
6
4
2
0
Liver weight (g)
Normal
VPCTN
DENA
DENA/VPCTN

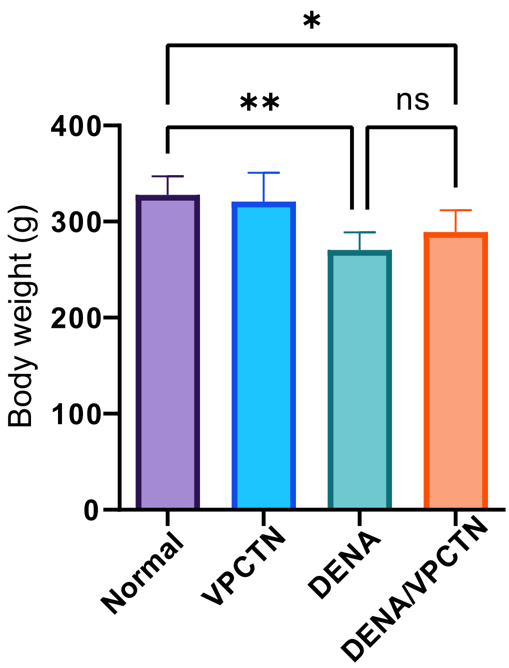

*
**
ns
400
300
200
100
0
Body weight (g)
Normal
VPCTN
DENA
DENA/VPCTN

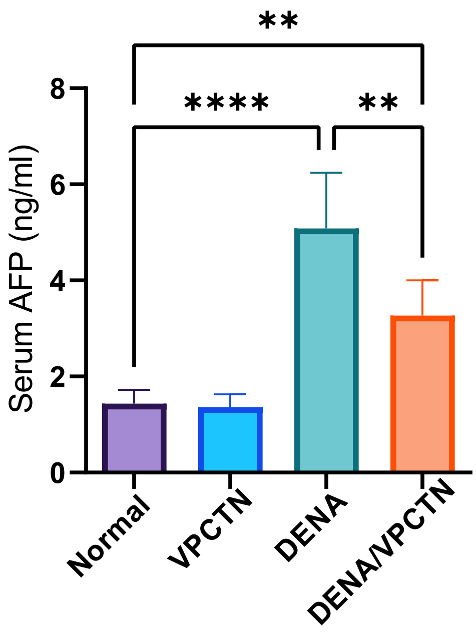

**
****
**
Serum AFP (ng/ml)
8
6
4
2
0
Normal
VPCTN
DENA
DENA/VPCTN

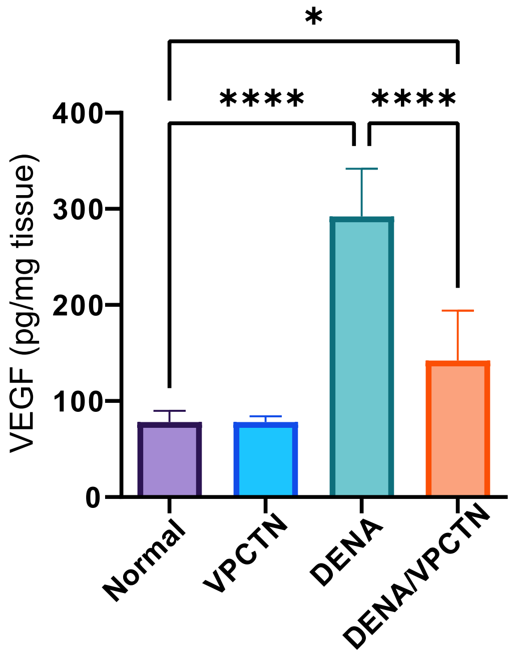
*
****
****
400
300
200
100
0
VEGF (pg/mg tissue)
Normal
VPCTN
DENA
DENA/VPCTN

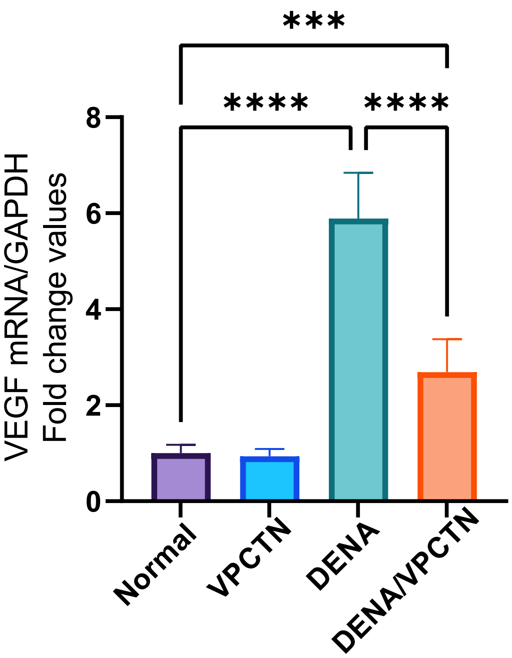

***
****
****
8
6
4
2
0
VEGF mRNA/GAPDH
Fold change values
Normal
VPCTN
DENA
DENA/VPCTN

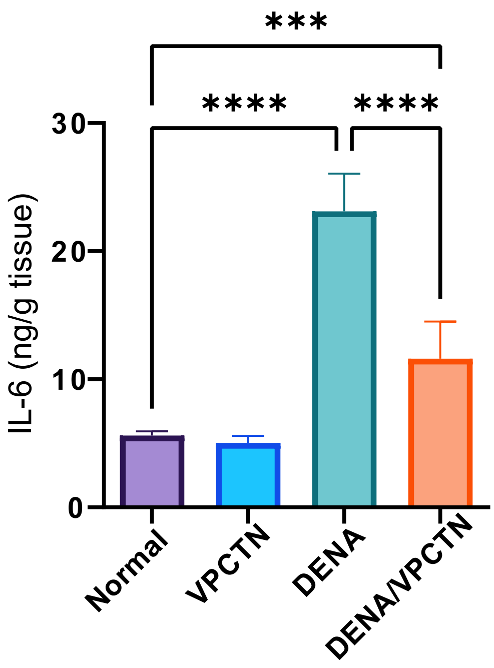

***
****
****
30
20
10
0
IL-6 (ng/g tissue)
Normal
VPCTN
DENA
DENA/VPCTN

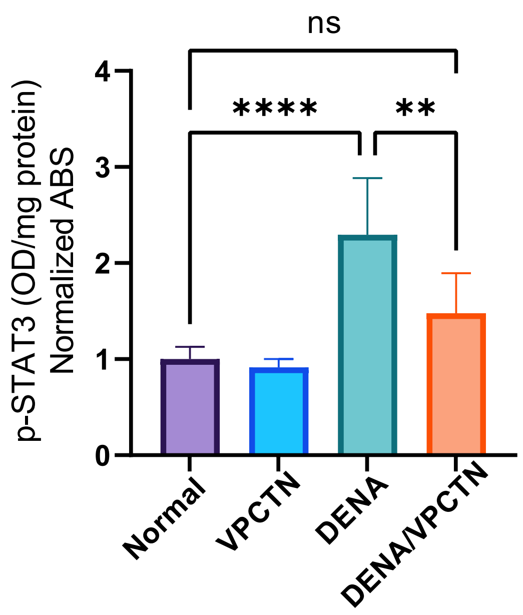
ns
****
**
4
3
2
1
0
p-STAT3 (OD/mg protein)
Normalized ABS
Normal
VPCTN
DENA
DENA/VPCTN

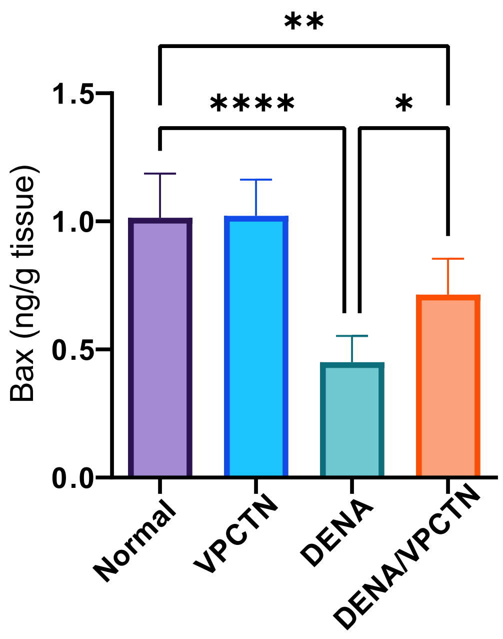

**
****
*
1.5
1.0
0.5
0.0
Bax (ng/g tissue)
Normal
VPCTN
DENA
DENA/VPCTN

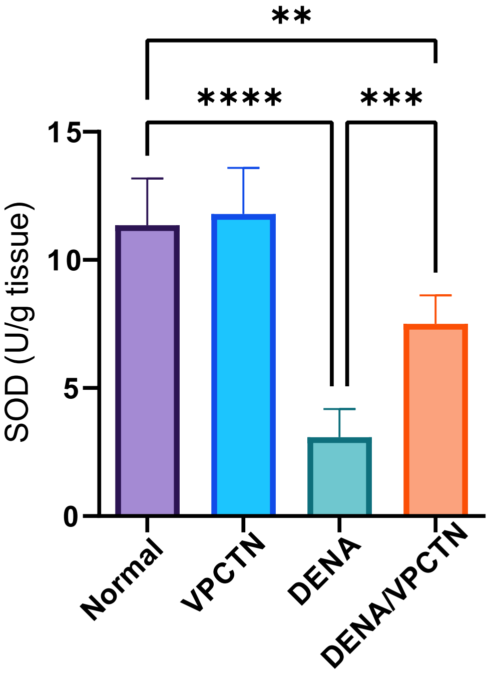
**
****
***
15
10
5
0
SOD (U/g tissue)
Normal
VPCTN
DENA
DENA/VPCTN

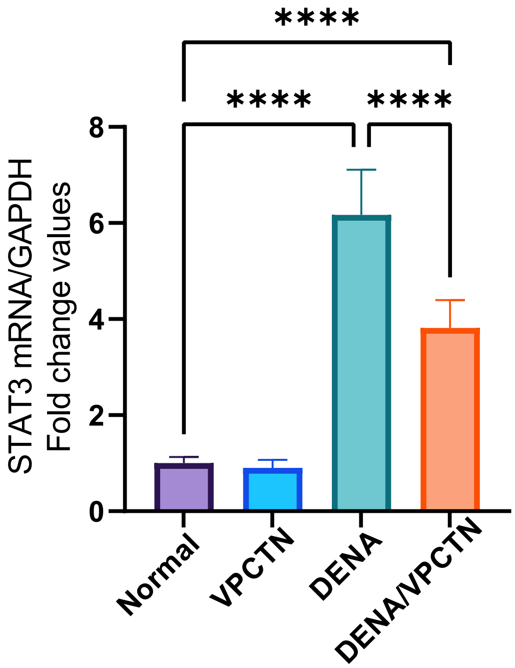
****
****
****
STAT3 mRNA/GAPDH
Fold change values
8
6
4
2
0
Normal
VPCTN
DENA
DENA/VPCTN

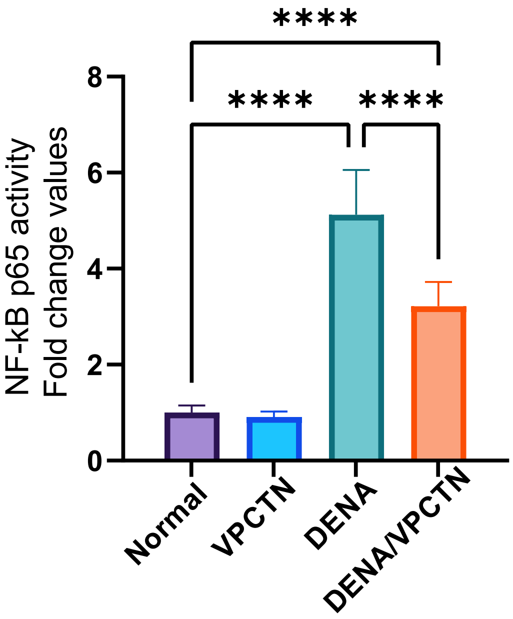
****
****
****
NF-kB p65 activity
Fold change values
8
6
4
2
0
Normal
VPCTN
DENA
DENA/VPCTN

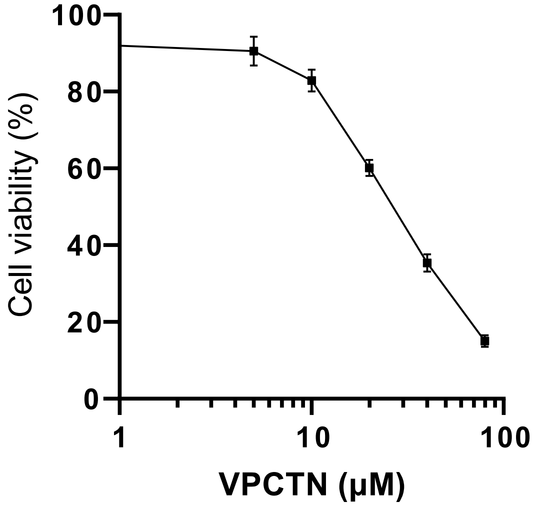

100
80
60
40
20
0
Cell viability (%)
1
10
100
VPCTN (μM)

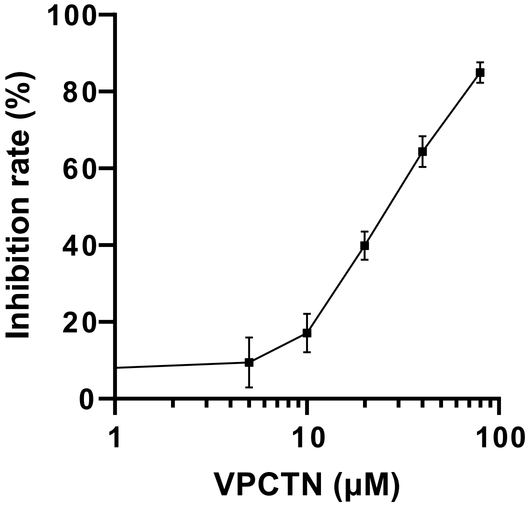

100
80
60
40
20
0
Inhibition rate (%)
1
10
100
VPCTN (μM)

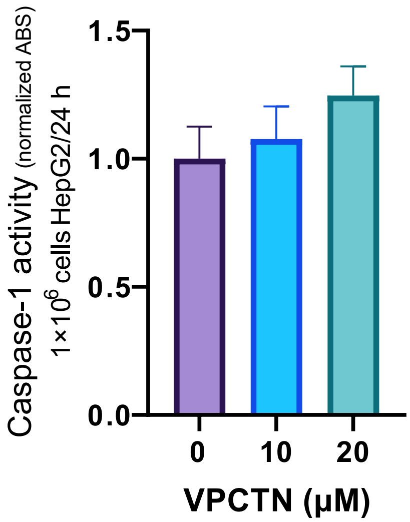
Caspase-1 activity (normalized ABS)
1×10^6 cells HepG2/24 h
1.5
1.0
0.5
0.0
0
10
20
VPCTN (μM)

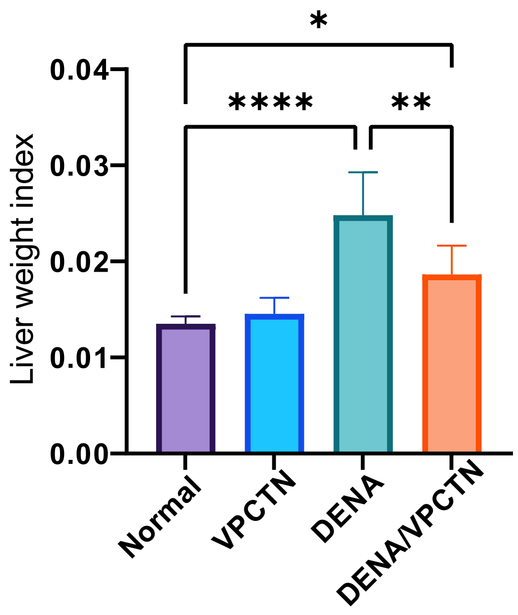

*
****
**
0.04
0.03
0.02
0.01
0.00
Liver weight index
Normal
VPCTN
DENA
DENA/VPCTN

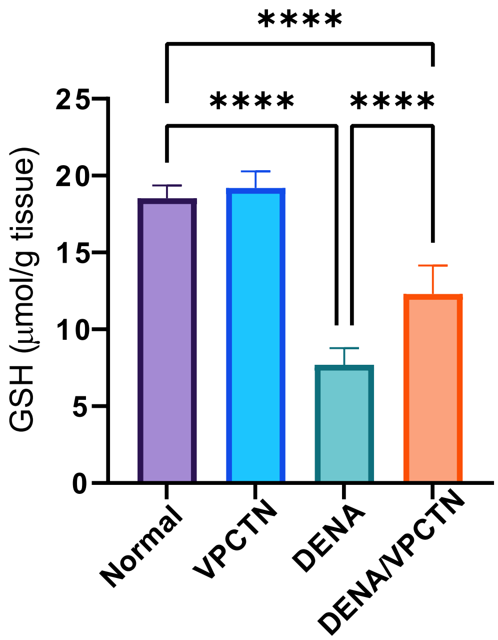
****
****
****
GSH (µmol/g tissue)
25
20
15
10
5
0
Normal
VPCTN
DENA
DENA/VPCTN

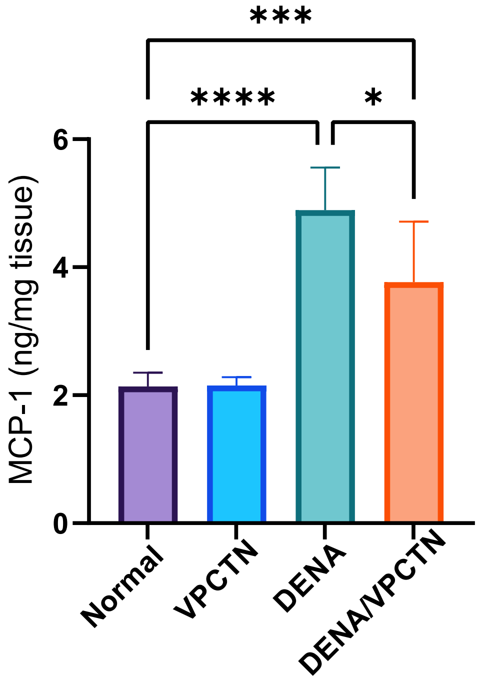

***
****
*
MCP-1 (ng/mg tissue)
6
4
2
0
Normal
VPCTN
DENA
DENA/VPCTN

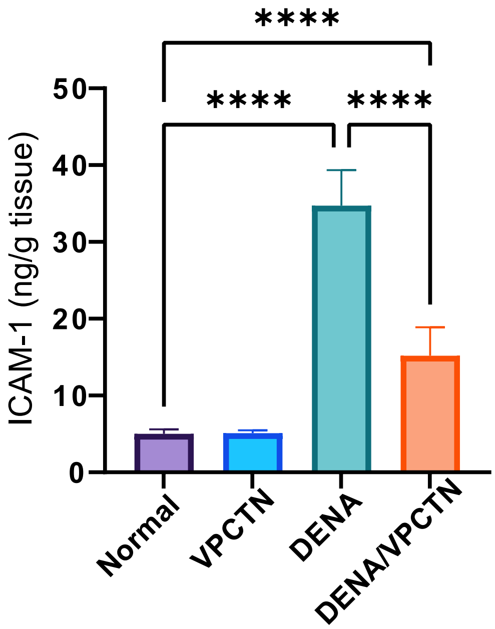
****
****
****
50
40
30
20
10
0
ICAM-1 (ng/g tissue)
Normal
VPCTN
DENA
DENA/VPCTN

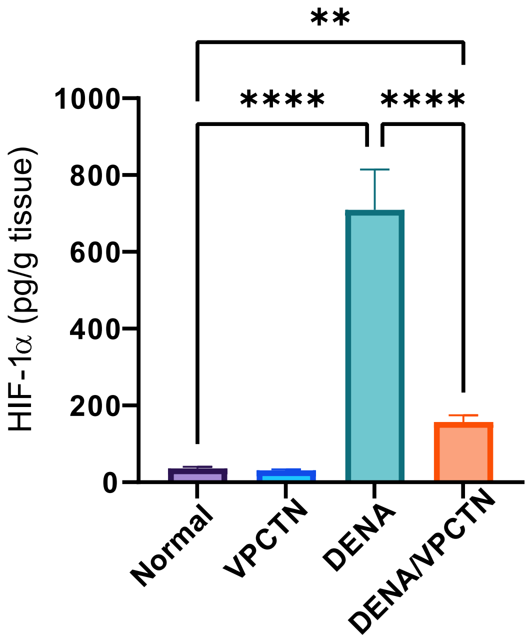

**
****
****
1000
800
600
400
200
0
HIF-1α (pg/g tissue)
Normal
VPCTN
DENA
DENA/VPCTN

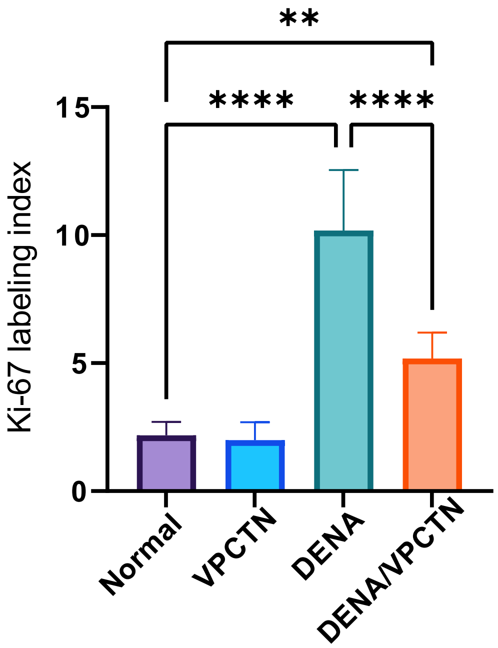
**
****
****
Ki-67 labeling index
15
10
5
0
Normal
VPCTN
DENA
DENA/VPCTN

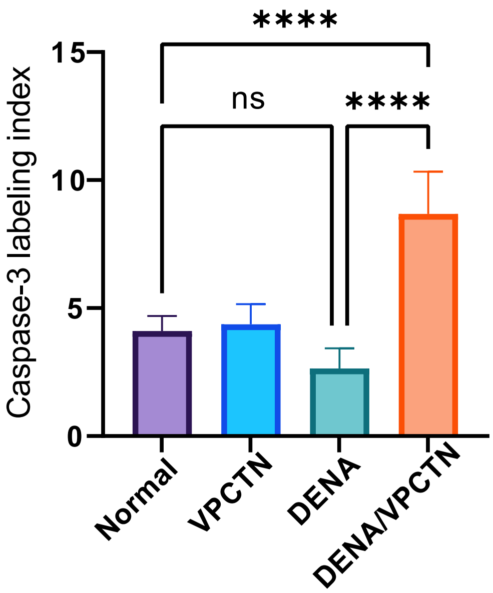

****
ns
****
15
10
5
0
Caspase-3 labeling index
Normal
VPCTN
DENA
DENA/VPCTN

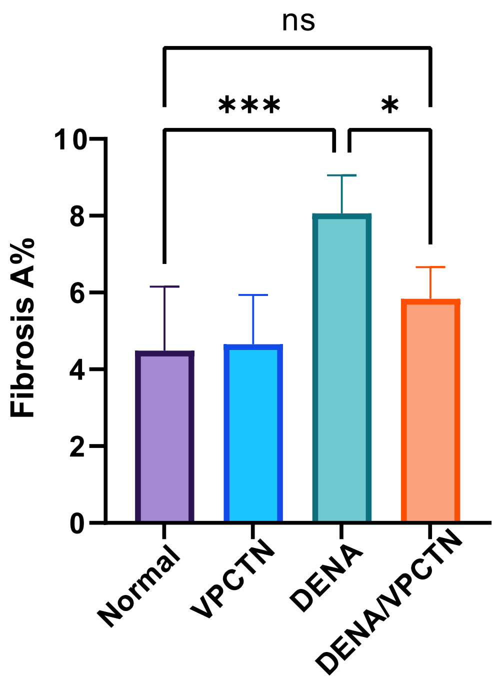
ns
***
*
Fibrosis A%
10
8
6
4
2
0
Normal
VPCTN
DENA
DENA/VPCTN

Ct values of 6 samples in each group

Calibrator Group:

- 28.1, 27.85, 28.05, 27.8, 28.12, 27.92

VPCTN Group:

- 28.209, 27.939, 28.179, 27.989, 28.169, 27.969

DENA Group:

- 26.246, 24.546, 26.146, 24.596, 26.196, 24.746

DENA/VPCTN Group:

- 27.171, 26.071, 27.021, 26.171, 27.121, 26.221
- **Ct value of reference gene = 18**
- **Calibrator Group ΔCt:**
  - 28.1−18=10.1
  - 27.85−18=9.85
  - 28.05−18=10.05
  - 27.8−18=9.8
  - 28.12−18=10.12
  - 27.92−18=9.92

Average ΔCt (Calibrator): 9.9733

- **VPCTN Group ΔCt:**
  - 28.209−18=10.209
  - 27.939−18=9.939
  - 28.179−18=10.179
  - 27.989−18=9.989
  - 28.169−18=10.169
  - 27.969−18=9.969

Average ΔCt (VPCTN): 10.0757

- **DENA Group ΔCt:**
  - 26.246−18=8.246
  - 24.546−18=6.546

- 26.146−18=8.146
- 24.596−18=6.596
- 26.196−18=8.196
- 24.746−18=6.746

Average ΔCt (DENA): 7.4133

- **DENA/VPCTN Group ΔCt:**
    - 27.171−18=9.171
    - 26.071−18=8.071
    - 27.021−18=9.021
    - 26.171−18=8.171
    - 27.121−18=9.121
    - 26.221−18=8.221

Average ΔCt (DENA/VPCTN): 8.463

- **Calibrator Group:**
    - Mean ΔCt: 9.9733
    - SD: 0.1353
- **VPCTN Group:**
    - Mean ΔCt: 10.0757
    - SD: 0.1223
- **DENA Group:**
    - Mean ΔCt: 7.4127
    - SD: 0.8612
- **DENA/VPCTN Group:**
    - Mean ΔCt: 8.6293
    - SD: 0.5248
